# Airborne Lidar Measurements of XCO_2_ in Synoptically Active Environment and Associated Comparisons With Numerical Simulations

**DOI:** 10.1029/2021JD035664

**Published:** 2022-08-17

**Authors:** Samantha Walley, Sandip Pal, Joel F. Campbell, Jeremy Dobler, Emily Bell, Brad Weir, Sha Feng, Thomas Lauvaux, David Baker, Nathan Blume, Wayne Erxleben, Tai‐Fang Fan, Bing Lin, Doug McGregor, Michael D. Obland, Chris O'Dell, Kenneth J. Davis

**Affiliations:** ^1^ Department of Geosciences Atmospheric Science Division Texas Tech University Lubbock TX USA; ^2^ NASA Langley Research Center (LaRC) Hampton VA USA; ^3^ Spectral Sensor Solutions LLC Fort Wayne IN USA; ^4^ Colorado State University Fort Collins CO USA; ^5^ Universities Space Research Association Columbia MD USA; ^6^ NASA Goddard Space Flight Center Greenbelt MD USA; ^7^ Atmospheric Sciences and Global Change Division Pacific Northwest National Laboratory Richland WA USA; ^8^ Department of Meteorology and Atmospheric Science The Pennsylvania State University University Park PA USA; ^9^ LSCE ‐ IPSL CEA Saclay Saclay France; ^10^ L3 Harris Technologies Melbourne FL USA; ^11^ Science System and Application, Inc Hampton VA USA; ^12^ Earth and Environmental Systems Institute The Pennsylvania State University University Park PA USA

**Keywords:** airborne atmospheric measurements, column average CO_2_ dry air mole fraction, cold front, greenhouse gases, mid‐latitude cyclone

## Abstract

Frontal boundaries have been shown to cause large changes in CO_2_ mole‐fractions, but clouds and the complex vertical structure of fronts make these gradients difficult to observe. It remains unclear how the column average CO_2_ dry air mole‐fraction (XCO_2_) changes spatially across fronts, and how well airborne lidar observations, data assimilation systems, and numerical models without assimilation capture XCO_2_ frontal contrasts (ΔXCO_2,_ i.e., warm minus cold sector average of XCO_2_). We demonstrated the potential of airborne Multifunctional Fiber Laser Lidar (MFLL) measurements in heterogeneous weather conditions (i.e., frontal environment) to investigate the ΔXCO_2_ during four seasonal field campaigns of the Atmospheric Carbon and Transport‐America (ACT‐America) mission. Most frontal cases in summer (winter) reveal higher (lower) XCO_2_ in the warm (cold) sector than in the cold (warm) sector. During the transitional seasons (spring and fall), no clear signal in ΔXCO_2_ was observed. Intercomparison among the MFLL, assimilated fields from NASA's Global Modeling and Assimilation Office (GMAO), and simulations from the Weather Research and Forecasting‐—Chemistry (WRF‐Chem) showed that (a) all products had a similar sign of ΔXCO_2_ though with different levels of agreement in ΔXCO_2_ magnitudes among seasons; (b) ΔXCO_2_ in summer decreases with altitude; and (c) significant challenges remain in observing and simulating XCO_2_ frontal contrasts. A linear regression analyses between ΔXCO_2_ for MFLL versus GMAO, and MFLL versus WRF‐Chem for summer‐2016 cases yielded a correlation coefficient of 0.95 and 0.88, respectively. The reported ΔXCO_2_ variability among four seasons provide guidance to the spatial structures of XCO_2_ transport errors in models and satellite measurements of XCO_2_ in synoptically‐active weather systems.

## Key Points

- First airborne observations of column average CO_2_ dry‐air mole‐fraction (XCO_2_) changes across fronts observed during ACT‐A are reported
- XCO_2_ frontal structures compare reasonably well with Weather Research and Forecasting—Chemistry and an in situ data driven assimilation system
- Results reveal that the differences across models and data were generally much smaller than the magnitude of XCO_2_ frontal contrasts

## 1 Introduction

Greenhouse gases play an essential role in governing Earth's radiation budget while atmospheric CO_2_ has been rising at an increasing rate (e.g., 1.50 ppm/year for 1990–1999, 1.97 ppm/year for 2000–2009, 2.40 ppm/year for 2010–2019; Peters et al., 2020). Very recently, the daily average of atmospheric CO_2_ concentrations at Mauna Loa observatory in Hawaii was recorded as high as 421.21 ppm (NOAA‐GML). Human activities, such as burning fossil fuels and deforestation, disturb the natural balance between CO_2_ sources and sinks and are causing this increase (Salam & Noguchi, 2005; Schneider et al., 2021), making quantification of CO_2_ sources and sinks essential to long term climate monitoring (Barnes et al., 2016; Masarie et al., 2014). Terrestrial ecosystem carbon fluxes are particularly uncertain, and earth system models differ vastly in their simulations of projected terrestrial CO_2_ uptake in a rapidly changing climate (e.g., Keenan & Williams, 2018).

An improved understanding of the spatiotemporal changes in atmospheric CO_2_ due to weather systems will strengthen our ability to infer uptake and release of CO_2_ from terrestrial ecosystems and the ocean through inverse methods. High‐resolution observations of CO_2_ vertical and spatial variability across different spatial scales (e.g., local, synoptic, global) help resolve estimates of surface fluxes at global and regional scales (e.g., Chevallier et al., 2010). Barnes et al. (2016) attributed the recent changes in northern high latitude seasonal cycle CO_2_ amplitude to changes in midlatitude surface fluxes but lacked observations of isentropic transport of CO_2_ across latitudes. Schuh et al. (2019) reported that large‐scale transport uncertainty resulted in a 1.7 PgC/year bias present in the inverse estimation in both high latitude bands (45°N–90°N and 45°S–90°S).

As air masses transport over different ecosystems, the CO_2_ spatial variability is primarily affected by underlying fluxes (Lan et al., 2017; Sweeney et al., 2015) and atmospheric boundary layer (ABL) features (Lee et al., 2015, 2018; Pal et al., 2017). The impact of synoptic air mass transport on CO_2_ vertical and horizontal distributions remained unclear as annual average vertical variability was presented without consideration of airmass differences (Sweeney et al., 2015). Synoptic‐scale processes modulate fluxes via cloud‐radiation feedback (Chan et al., 2004), and precipitation‐soil moisture–atmosphere feedback (Humphrey et al., 2021). However, a significant gap remains in our understanding of the impact of synoptic‐scale weather systems and associated vertical exchanges due to convection on CO_2_ transport.

Previously, Hurwitz et al. (2004) and Lee et al. (2012) used NOAA's tall tower measurements to investigate the effects of frontal passages on the CO_2_ variability in the lower‐most part of the ABL, noted rapid changes in ABL‐CO_2_, and speculated about the presence of large CO_2_ gradients in the troposphere. Using airborne in situ measurements, Pal, Davis, Lauvaux, et al. (2020) showed how the CO_2_ distributions in the ABL, and free troposphere (FT) vary spatially across individual frontal systems during summer and also reported the presence of an enhanced band of CO_2_ in the ABL in the vicinity of frontal boundaries. However, it remained unclear how CO_2_ distributions in the entire lower troposphere change in presence of synoptic scale events (e.g., frontal passage, squall lines).

High‐resolution (e.g., 1 km spatially) airborne lidar measurements of XCO_2_ across frontal boundaries would illustrate the CO_2_ spatial variability in the entire lower troposphere and can potentially complement in situ CO_2_ observations, and aid in satellite validation (e.g., Campbell et al., 2020). Bell et al. (2020) provided examples of the accuracy and ability of the Orbiting Carbon Observatory ‐ 2 (OCO‐2) to observe XCO_2_ spatial variability in fair weather via comparison with both in situ data assimilated into a curtain by an atmospheric model and observations from the airborne Multifunctional Fiber Laser Lidar (MFLL).

Satellite measurements provide a valuable tool for analyzing global CO_2_ measurements. However, because OCO‐2 makes passive measurements and depends on reflected sunlight, clouds inhibit this process, yielding high uncertainties in the measurements obtained in cloudy regimes (O'Dell et al., 2018). The Total Carbon Column Observing Network (TCCON) provides temporal variability of XCO_2_ with respect to a synoptic scale weather passage (Wunch et al., 2011); however, TCCON measurements also contain gaps and can become erroneous in presence of clouds. Additionally, due to the sparse network, the horizontal XCO_2_ spatial variability remains unresolved (Chevallier et al., 2011; O'Dell et al., 2012). On the other hand, OCO‐2 derived fields of XCO_2_ are also not adequate to obtain samples across frontal boundaries due to excessive cloudiness often present near frontal boundaries (Bell et al., 2020; O'Dell et al., 2018; Wunch et al., 2017).

High spatially resolved information of XCO_2_ variability in the presence of synoptic‐scale weather patterns are available from the airborne lidar observations collected during the Atmospheric Carbon and Transport—America (ACT‐America) field campaigns (Davis et al., 2021; Pal & Davis, 2021). The primary benefit of the airborne lidar measurements over satellite or TCCON measurements are the lidar's potential to obtain measurements through thin or scattered clouds, and the fact that the aircraft often flew below high clouds. Aircraft measurements sampled XCO_2_ variability across frontal boundaries and provide adequate measurements in warm and cold sectors within a brief time (∼1 hr; Bell et al., 2020; Campbell et al., 2020).

**Figure 1:**
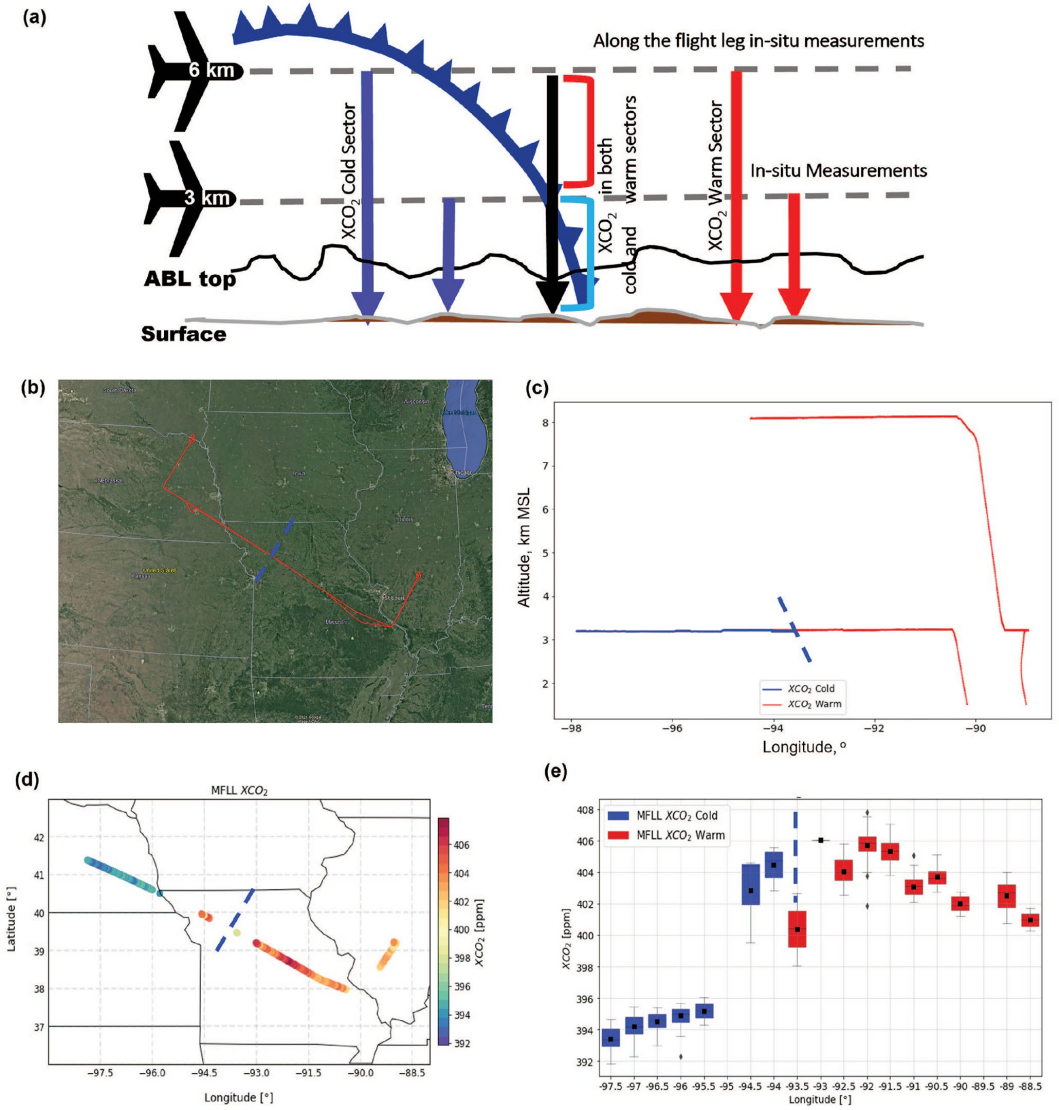
Schematic of a frontal research flight (RF) showing multifunctional fiber laser lidar (MFLL) measurements onboard C‐130 from two altitudes (3 and 6 km MSL) sampling XCO_2_ in both the warm (red arrows) and cold (blue arrows) sectors of the front. The region near the frontal boundary (slanted navy curve with triangles) indicates XCO_2_ measurements referring to both warm and cold sector columns in vertical. In situ measurements of CO_2_ were also performed along those legs. Black and gray curved lines mark the atmospheric boundary Layer (ABL) top and surface, respectively (a); C‐130 track (red line overlaid on the map) during the RF on 12 August 2016 (b); Longitude‐versus‐altitude view of the frontal flags used to indicate warm (red) and cold (blue) sectors along C‐130 tracks (c); XCO_2_ (3 km MSL column) spatial variability across the front (d); 0.5⁰ longitude segmented box and whisker plot across the frontal boundary where the blue and red boxes represent XCO_2_ in the cold and warm sector, respectively (e); The black square and horizontal line inside the boxes indicate the mean and median, respectively. The end of the bars represents points that lie within 1.5 interquartile ranges, with the black diamonds representing values that fall outside of that range. The dashed blue line on each panel marks the frontal boundary.

The key aims of this work are to investigate (a) the impact of mid‐latitude cyclones on XCO_2_ spatial variability, in particular, XCO_2_ frontal contrasts (i.e., ΔXCO_2_), over the eastern US in four seasons; (b) how well the global and mesoscale transport model simulations capture ΔXCO_2_; and (c) how model‐data mismatches (MDMs) vary between frontal and fair weather conditions. These investigations help obtain the typical spatial distribution of XCO_2_ variability in the atmosphere during frontal passages (Figure 1). Since for the first time, we collected systematic MFLL measurements across frontal boundaries from different altitudes, it remains beneficial to demonstrate the potential of MFLL measurements in heterogeneous water vapor environment (i.e., warm moist sector vs. dry cold sector), typically involved during the passage of mid‐latitude cyclones. We also explored XCO_2_ retrieved by WRF‐Chem simulations, that is not optimized by ACT‐America measurements but is fed NOAA CarbonTracker fluxes and boundary conditions (Peters et al., 2007; with updates documented at https://carbontracker.noaa.gov). Finally, we also used XCO_2_ variability estimated using a different transport model that is optimized using ACT‐America in situ measurements from NASA's GMAO to investigate ΔXCO_2_.

The remaining parts of the paper are organized as follows. Sections 2 introduces the datasets, instruments and model simulation used while Section 3 presents the details of the methodology to estimate XCO_2_ frontal contrasts, enhancement, and MDMs in all the relevant metrics where we provided an example case to illustrate the MDM framework. Results pertaining to observed XCO_2_ frontal contrasts, MDMs, and frontal enhancement are reported in Section 4. Followed by an extended discussion in Section 5, and brief conclusions and outlook are finally provided in Section 6.

## 2 Data Set, Instruments, and Models

### 2.1 ACT‐America

Within the ACT‐America project, five field campaigns were carried out (summer‐2016, winter‐2017, fall‐2017, spring‐2018, and summer‐2019) over three eastern US regions: Mid‐Atlantic (MA), Midwest (MW), and South (SO) (Davis et al., 2021); during the summer‐2019 campaign, the MFLL was not deployed. During most research flights (RFs), two aircraft were flown (B‐200 and C‐130) to obtain reactive trace gases (CO, O_3_), greenhouse gases (CO_2_, CH_4_) and meteorological measurements during fair weather and frontal weather conditions (Davis et al., 2018; Pal & Davis, 2021; Wei et al., 2021). The C‐130 aircraft flew at several different altitudes, often in the upper and lower FT, and acquired MFLL measurements of XCO_2_ (Lin et al., 2020). The B‐200, housing a suite of similar in situ instruments, flew in the ABL and mid FT, typically collocated along a portion of the C‐130 ground tracks so that both CO_2_ and XCO_2_ observations along with the meteorological measurements are collected along similar flight paths across the fronts.

During the first four campaigns, 38 frontal RFs were conducted, but some of the high‐altitude flights (e.g., 4.5, 5.5 and 8 km MSL) were eliminated for this study due to the lack of XCO_2_ measurements for extensive cloud cover beneath the MFLL platform at the lower altitudes, mostly in the warm sector of the frontal systems (e.g., see Figure 1c for frontal flags along 8 km MSL altitude during a RF on 12 Aug 2016). All the frontal RFs available through ACT‐America are described in Table S1 (see Supporting Information S1). One should note that the C‐130 flight altitudes varied for the selected cases (2.5–8 km MSL, Table S1 in Supporting Information S1). To achieve the goals of this work, we required XCO_2_ samples in both warm and cold sectors to investigate XCO_2_ frontal contrasts. Many high‐altitude flights sampled only one of the two frontal sectors. Additionally, some frontal RFs were made in such a way that MFLL observations were not useful due to the changing altitudes of the MFLL platform (C‐130). Though it is possible to obtain XCO_2_ for the altitudes between cloud top and MFLL platform (i.e., partial columns without the entire depth of the atmosphere from MFLL platform to ground), we did not consider those measurements to be appropriate to fulfill our research goals. Previously, Lin et al. (2020) discussed the flagging and retrieval of XCO_2_ in those partial columns. To the best of the authors' knowledge, there exist no systematic XCO_2_ measurements across frontal boundaries in the literature.

Finally, 27 cases were selected for the investigation of the XCO_2_ frontal contrasts (henceforth, ΔXCO_2_). The ideal C‐130 flight pattern for frontal RFs included 2 level flight legs of C‐130 across a frontal boundary, varying in altitude from lower to upper free troposphere (Figure 1a) which illustrates that quantification of ΔXCO_2_ using MFLL‐XCO_2_ measurements remains complicated by the slanted shape of the frontal boundary. Thus, we considered the XCO_2_ measurements far from the frontal boundary to compute warm and cold sector averages of XCO_2_ to estimate the ΔXCO_2_ and analyzed the XCO_2_ measurements near the vicinity of frontal boundary separately to understand the front relative XCO_2_ features (an enhancement in XCO_2_ spatial structures, see Section 3.3).

### 2.2 Multifunctional Fiber Laser Lidar (MFLL)

The MFLL was developed by Harris Corp. and NASA Langley Research Center (Dobler et al., 2013). The MFLL uses the Integrated Path Differential Absorption (IPDA) technique to measure the differential absorption optical depth (DOAD) of the online and offline wavelengths at the CO_2_ absorption line centered at 1,571.112 nm (Campbell et al., 2020). Optical depths from the MFLL data and meteorological variables from the Modern‐Era Retrospective analysis for Research and Applications, Version 2 (MERRA‐2; Gelaro et al., 2017) reanalysis data were then used to retrieve XCO_2_ (Campbell et al., 2020). Additional details on the XCO_2_ retrieval, its spectroscopic model, etc. are available elsewhere (e.g., Bell et al., 2020; Campbell et al., 2020; Gordon et al., 2017) while MFLL weighting function and Lite files are available in Lin et al. (2022a, 2022b).

During the XCO_2_ retrievals, a number of calibration and corrections were applied including (a) filtering lidar signals collected for more than 5° pitch/roll angles, (b) cloud‐screening via discriminating the signals between ground and intermediate scatterers (i.e., clouds) using the matched filter technique, (c) altitude dependent bias correction using CO_2_ profiles obtained at cloud‐free conditions and modeled XCO_2_ values obtained using in situ measurements of meteorological parameters and CO_2_ concentrations (Campbell et al., 2020). The MFLL‐XCO_2_ measurements were found to have a signal‐to‐noise ratio (SNR) of 120, 330, 950 and 1,600 for averaging times of 0.1, 1, 10 and 60 s, corresponding to a precision of 3.4, 1.2, 0.43, and 0.26 ppm, respectively. The XCO_2_ measurement precision was estimated by the standard deviation of the measured XCO_2_ samples with the stated averaging (i.e., 0.1, 1, 10, and 60 s) under spatially homogeneous conditions while the SNR was estimated via calculating mean XCO_2_ divided by the standard deviation of XCO_2_ during certain flight length.

The results pertaining to the measurement precision and SNR were obtained via dedicated MFLL flights over the Gulf of Mexico under relatively homogeneous conditions in presence of onshore flow. For instance, the mean CO_2_ concentrations (using in situ measurements on C‐130 aircraft) along two different ABL legs across a distance of more than 200 km over water were found to be 405.15 and 405.17 ppm while the corresponding standard deviations were 0.09 and 0.08 ppm, respectively. Additionally, on a one‐hour time scale, the average drift was below 0.1 ppm. Campbell et al. (2020) provided some comprehensive details on calibration measurement performance of MFLL under different atmospheric conditions.

### 2.3 The GMAO Curtain

NASA's GMAO produces fields of XCO_2_ through the assimilation of in situ CO_2_ measurements collected using both the C‐130 and B‐200 aircraft, referred to here as curtains (Bell et al., 2020). The GMAO product has 72 vertical levels from the surface to 0.01 hPa with a 0.5⁰ by 0.625⁰ spatial resolution is output every 3 hr and recently has been used as part of the calibration and validation of MFLL retrievals at 8 km (Campbell et al., 2020). The GMAO product is used here as a proxy for in situ data as it fills in data where it is missing using modeled correlations. It uses fluxes calibrated to in situ data (Weir et al., 2021) that are similar to the input fluxes used by WRF‐Chem. Using a similar approach introduced in Bell et al. (2020), we used XCO_2_ retrieved from the GMAO assimilated curtains (henceforth, GMAO‐XCO_2_) to explore ΔXCO_2_.

### 2.4 The WRF‐Chem Model

The Weather Research and Forecasting‐—Chemistry (WRF‐Chem) model derived XCO_2_ field was compared to the MFLL observations to evaluate the XCO_2_ variability in this modeling system (Feng, Lauvaux, Davis, et al., 2019; Skamarock et al., 2008). The CO_2_ fluxes included in WRF‐Chem were obtained from NOAA CarbonTracker (CT) v2017 for summer‐2016 and Near Real Time v2019‐2 (CT‐NRT.v 2019‐2) for the other three campaigns beyond 2016. CO_2_ was included as a passive tracer in WRF‐Chem (Lauvaux et al., 2012). In general, CT assimilates observations at the rate of each measurement (from hourly to weekly depending on types of available observations) and at resolution of eco‐regions for land and large ocean regions, and weekly scaling factors for each flux regions. CT outputs are available at 3‐hourly temporal resolution at 1⁰ *×* 1⁰ spatial resolution and provides the carbon surface fluxes from the terrestrial biosphere, oceans, fossil fuels, wildfires, and atmospheric CO_2_ mole fractions (Jacobson et al., 2007, 2020). Additional details on the WRF‐Chem model setup and performance can be found elsewhere (Feng, Lauvaux, Keller, et al., 2019; Gerken et al., 2021; Samaddar et al., 2021).

WRF‐Chem was run at 27‐km covering most of North America from 1 June 2016 to 30 June 2019. In the entire simulation period, the meteorology was driven with ERA5 and run 5 days including a 12‐hr spin‐up; the first month is considered the CO_2_ spin up, and CO_2_ is carried over in each meteorological cold start. At the end, we concatenated the 4.5 days simulation in each simulation window, and all the analyses used here are based on them.

### 2.5 Computing XCO_2_ for Comparison to MFLL

Following Bell et al. (2020), simulated XCO_2_ was trimmed down and interpolated to match the MFLL observations along the C‐130 tracks during all the RFs so that XCO_2_ field from both WRF‐Chem (henceforth, WRF‐XCO_2_) and GMAO were available at a spatial resolution of 1,100 m corresponding to each XCO_2_ sample collected using MFLL at a temporal resolution of 10 s (i.e., equivalent to horizontal distance of 1,100 m with an average C‐130 aircraft speed of 110 m s^−1^). For intercomparison of XCO_2_ fields, we did not use GMAO‐XCO_2_ and WRF‐XCO_2_ when or where MFLL had data gaps to keep a similar number of XCO_2_ samples in simulation and observations.

The MFLL uses a pressure weighting function (PWF) which favors CO_2_ concentrations at higher altitudes in the observed column and which can affect the observed XCO_2_ in non‐negligible ways when compared to a “straight” pressure‐weighted calculation (Bell et al., 2020). The MFLL PWF was derived for each individual sounding along a flight track; we used the coincident MFLL PWFs to calculate XCO_2_ from the resampled GMAO and WRF‐Chem CO_2_ fields, ensuring that the vertical sensitivity is consistent between MFLL observations and model estimates. Following Bell et al. (2020), we sampled GMAO in situ “curtain” column up to the C‐130 height to produce a partial‐column value which uses a straight pressure weighting function.

## 3 Methods

### 3.1 Quality Control of MFLL‐XCO_2_ Measurements

The MFLL‐XCO_2_ had many anomalous data points not filtered by the correction approach outlined in Section 2.2. To remove the additional outliers, we developed a standard deviation filter method to eliminate these anomalous data points (Figure S1 in Supporting Information S1). Each case was analyzed individually to identify the appropriate standard deviation to be applied for the filter. The standard deviation filter was applied to both 10 s averaged and 1 s measurements of XCO_2_. The XCO_2_ differences between the filtered data at 1s and the averaged data with a filter taken at 10 s were analyzed, and the only samples with XCO_2_ differences between the two samples that were within ±1 ppm of XCO_2_ were kept for further analyses.

Before we applied the standard‐deviation based filter approach to remove outliers in XCO_2_ samples, we made an extensive amount of sensitivity tests for XCO_2_ measurements collected during both fair weather and frontal crossing RFs. Those tests were performed separately for each case and appropriate filter value was chosen. We applied the filters simultaneously in two stages, namely for 1 and 10‐s averaged XCO_2_ values. When the results of both averaged‐sets identified identical outliers (i.e., spike), we removed them for the reminder of the analyses. The histogram analyses for all the samples also yielded those identified samples (i.e., outliers) to be located far from the distributions as expected due to the application of the standard deviation approach. Being a conservative approach based on sensitivity tests for individual cases, we finally eliminated less than 0.01% of samples from the entire flight leg for each case, though it varied among the cases as evinced in the example results presented in Figure S1 in Supporting Information S1. Thus, we note the filtering technique did not change the frontal contrast results. In the past, a very similar standard‐deviation based approach (often called histogram analyses) was used in other lidar‐based studies to remove spikes from lidar measurements (e.g., Pal et al., 2010; Senff et al., 1994; Turner et al., 2014; Wulfmeyer et al., 2010).

This technique allowed for removing the outliers in all the cases, as illustrated in the example case of an MW flight on 30 October 2017 (Figure S1 in Supporting Information S1). The XCO_2_ (2.2 km MSL column) variability in a longitudinal plane across a cold front is presented with a direct comparison between the different filtered data and unfiltered data averaged to 10 s. As depicted by the yellow crosses, the anomalous data points are filtered out using the approach described above. In further analyses, we used only the measurements represented by the black stars. The spikes in the XCO_2_ field are most likely caused by low SNR in the presence of clouds.

However, we identified four frontal RFs (11 October 2017; 7 March 2017; 16 May 2018; 20 August 2016, Table S1 in Supporting Information S1) during which MFLL‐XCO_2_ measurements were extraordinally errroneous even after the application of the different corrections, calibration and the standard deviation filter. The MFLL overall accuracy for these cases was of lower quality most likely due to very high water vapor variability in the atmosphere that was not well represented in the reanalysis product used for retrieving XCO_2_. Consequently, we decided to eliminate the XCO_2_ measurments for those RFs for further analyses and for overall MDM explorations.

### 3.2 XCO_2_ Frontal Contrasts

The frontal boundary along the individual flight legs was determined based on the in situ measurements of thermodynamic variables, particularly temperature and dew point temperature, as described in Pal, Davis, Lauvaux, et al. (2020). As illustrated in Figure 1 (panels b, c, d, and e), in the vicinity of the frontal boundary (longitude of 93.5°W), the MFLL samples warm and cold sectors due to the natural slope or baroclinic feature of the frontal boundary. To avoid this region's inclusion in the XCO_2_ frontal contrast calculation, the flight track was divided into half‐degree segments of latitude or longitude on either side of the frontal boundary as indicated by the frontal flags. The frontal contrast (i.e., $\Delta {\text{XCO}}_{2}$) was calculated using

$$\Delta {\text{XCO}}_{2}=\;\bar{{\text{XCO}}_{2}^{\text{Warm}}}-\;\bar{{\text{XCO}}_{2}^{\text{Cold}}}\;$$

where $\bar{{\text{XCO}}_{2}^{\text{Warm}}}$ and $\bar{{\text{XCO}}_{2}^{\text{Cold}}}\;$ represent the average XCO_2_ in the furthest half‐degree segments of latitude or longitude depending on the frontal orientation along the flight track in the warm and cold sectors, respectively. Such selection far away from the frontal boundary provided the advantage that we were able to avoid selecting partial columns with mixture of warm and cold sector airmass (in vertical direction) in the vicinity of frontal boundaries (Figure 1a). For instance, if the C‐130 flight legs were oriented along the north to south plane crossing the frontal boundary orthogonally, we considered the farthest 0.5° latitude sectors in the north and south for cold and warm sectors, respectively. Thus, for the analyses of $\Delta XCO_{2}$ for all the RFs, we were able to exclude the XCO_2_ samples obtained in the “enhanced XCO_2_” region. Additionally, the lengths of the C‐130 flight legs in the warm and cold sector varied, with more sampling typically in the warm sector than in the cold sector for majority of the RFs. A similar approach was applied to calculate $\Delta {\text{XCO}}_{2}\;$ for both GMAO‐XCO_2_ and WRF‐XCO_2_.

**Figure 2:**
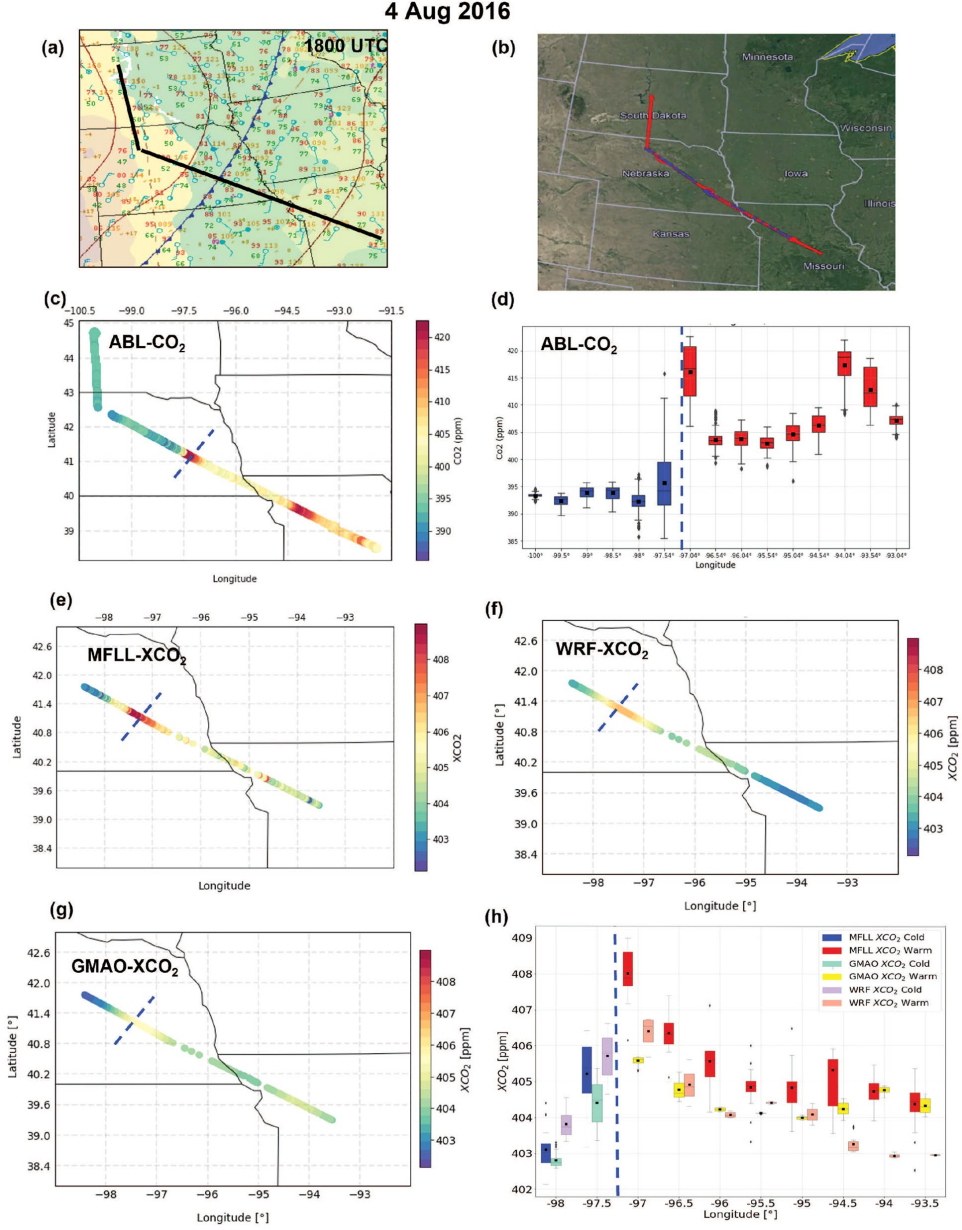
Model data comparison for an example frontal research flight (RF) in summer (4 August 2016, Figure 4) along with the flight tracks shown on a surface map (a) and on Google map; red: C‐130 and blue: B‐200 (b). ABL‐CO_2_ spatial variability (c); XCO_2_ (5.5 km MSL column) retrieved using MFLL (e), WRF‐Chem (f) and GMAO (g). The box and whisker plots for both ABL‐CO_2_ (d) and XCO_2_ from three products binned into 0.5⁰ longitude segments with XCO_2_ from the MFLL XCO_2_ in the warm (red) and cold (blue) sectors, WRF‐Chem in the warm (pink) and cold (purple) sectors, and GMAO in the warm (yellow) and cold (aqua) sectors. Blue dashed‐line on each panel marks the frontal boundary.

To illustrate the methodology for determining ΔXCO_2_, we presented MFLL‐XCO_2_ measurements obtained on 4 August 2016 over the MW region during the summer‐2016 field campaign (Figure 2). During this flight, the C‐130 flew a distance of 810 km across a frontal boundary located in central Missouri such that it was possible to obtain enough XCO_2_ samples in both warm and cold sectors. The MFLL measurements from an altitude of 5.5 km MSL suggested significant XCO_2_ spatial variability with higher XCO_2_ in the warm sector than in the cold sector. The frontal flags obtained along 5.5 km MSL leg clearly indicate the frontal boundary at the location of 94°W which was used as a reference for estimating ${\text{XCO}}_{2}$. The box and whisker analyses depict the average XCO_2_ at a resolution of 0.5°‐longitude segments (i.e., ∼50 km horizontal resolution, Figure 2h). The furthest average XCO_2_ (0.5°‐longitude segment) in the cold sector at −97.5⁰ was subtracted from the furthest average XCO_2_ (0.5°‐longitude segment) in the warm sector at −93.5⁰. From this, a ${\text{XCO}}_{2}$ of 2.1 ppm was found. Additionally, we found a very similar pattern in the spatial variability in ABL‐CO_2_ field and MFLL measured XCO_2_ field on this day although the sampling domains in longitudes were slightly different (Figures 2d and 2h).

### 3.3 XCO_2_ Enhancement Near the Frontal Boundary

Previously, while investigating frontal contrasts in CO_2_ concentrations in both ABL and FT for summer‐2016 field campaign, Pal, Davis, Lauvaux, et al. (2020) found a region of enhanced CO_2_ in the ABL in the vicinity of frontal boundaries. Using the MFLL measurements of the XCO_2_ field, a very similar feature was noted in some cases (Tables S1 and S2 in Supporting Information S1). For each case, the XCO_2_ enhancement feature was further investigated by analyzing the XCO_2_ average values in the enhanced region compared to the warm sector's average‐XCO_2_. The width of the enhanced XCO_2_ band was estimated by analyzing the mean XCO_2_ in the half‐degree longitude or latitude segments that were elevated near the frontal boundary compared to the warm‐sector average XCO_2_. The XCO_2_ enhancement was calculated by subtracting average warm sector XCO_2_ from the enhanced region's average XCO_2_.

### 3.4 Model Data Comparison

An intercomparison among the MFLL‐XCO_2_, GMAO‐XCO_2_, and WRF‐XCO_2_ was conducted to investigate the XCO_2_ spatial variability across frontal boundaries obtained via MFLL observations and two model fields. WRF‐Chem has not been optimized using the in situ airborne observations while GMAO‐simulations were used to create curtains using the ACT‐America in situ CO_2_ mole fraction observations. Two types of comparisons were conducted: a comparison of the $\Delta {\text{XCO}}_{2}$ and a comparison of the average XCO_2_ sampled in both warm and cold sectors.

**Figure 3:**
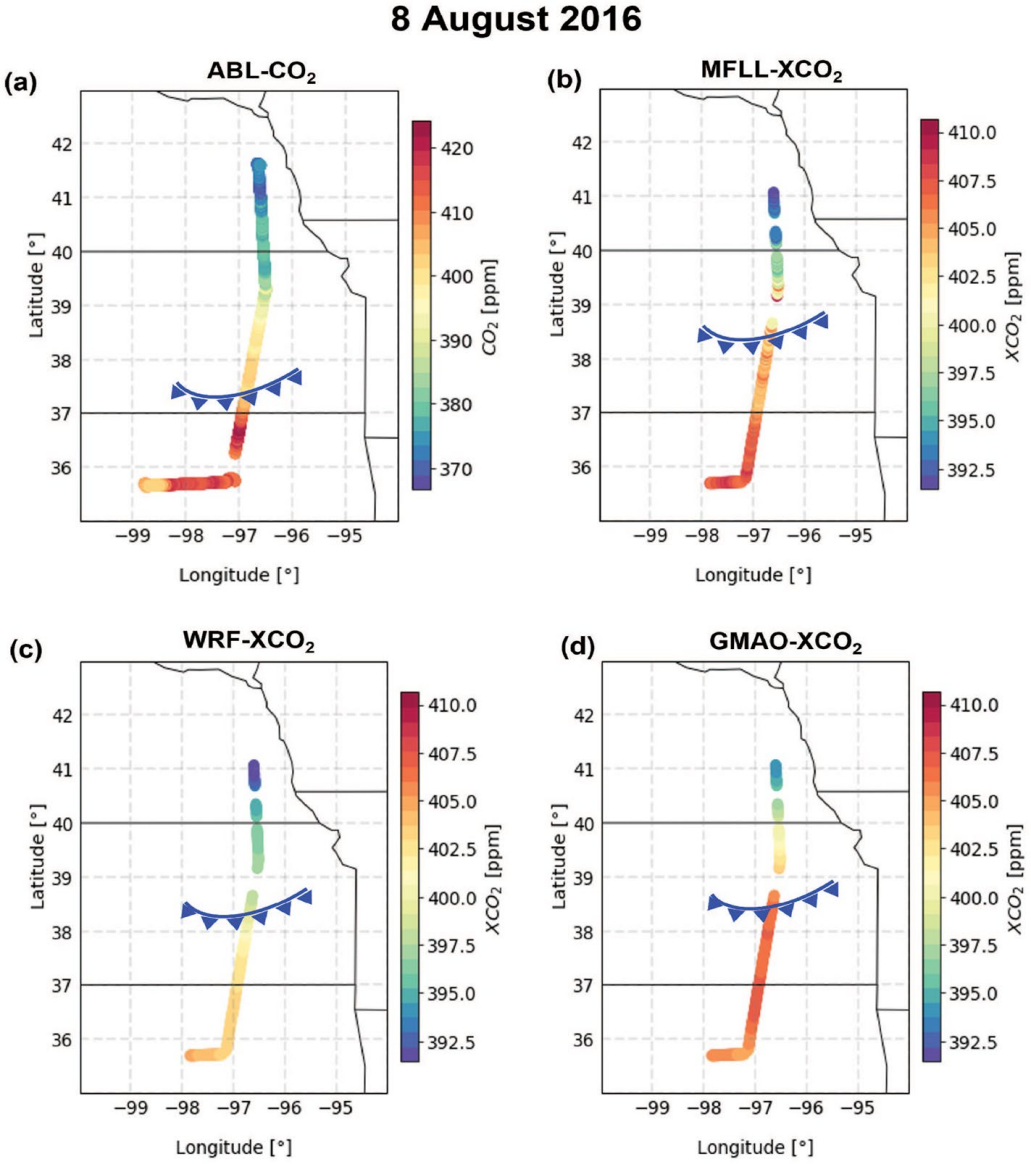
A multi‐parameter view of frontal crossing research flight on 8 August 2016 showing in situ measurements of CO_2_ mole fractions yielding ABL‐CO_2_ spatial variability (a), XCO_2_ derived using multifunctional fiber laser lidar (MFLL) on board C‐130 along the north‐south transect (b), WRF‐Chem simulation (c), and Global Modeling and Assimilation Office (GMAO) assimilated curtains (d). The MFLL XCO_2_ spatial variability across the frontal boundary at 3.5 km MSL was obtained after 0.5 hr of B‐200 sampled ABL‐CO_2_ along the same track. Note that the color bar scale limits for panel a (i.e., in situ CO_2_ measurements) differs from the other panels (XCO_2_ measurements). Location of frontal boundary on each panel is marked by a curved blue line with triangles.

Figure 3 shows the two different types of measurements (in situ measurements of ABL‐CO_2_ and MFLL‐XCO_2_), GMAO‐XCO_2_, and WRF‐XCO_2_ for an example frontal RF on 8 Aug 2016. This MW flight spanned across eastern Oklahoma, Kansas, and Nebraska, and the MFLL data was collected at around 3.5 km MSL. A cold front was located near the Oklahoma/Kansas border as shown on the ABL‐CO_2_ field (see frontal boundary in ABL at ∼37.3°N in Figure 3a). The MFLL measurements along the same track also shows very similar front‐relative features in XCO_2_ (Figure 3b) as was observed for the ABL‐CO_2_ field confirming higher values in both XCO_2_ and CO_2_ in the warm sector than the cold sector. The frontal boundary along the 3.5 km MSL flight leg (located ∼38.5°N) is also marked illustrating the frontal slope illustrated in Figure 1a. It can be seen that the overall XCO_2_ spatial variability observed by MFLL was captured well by both GMAO and WRF‐Chem (see Supporting Information S1 for similar analyses for all the frontal RFs). For brevity, a single case for the three other seasons is briefly described in Supporting Information (see Supporting Information S1) to exemplify the model data intercomparison framework.

## 4 Results

### 4.1 XCO_2_ Frontal Contrasts in Four Seasons

**Figure 4:**
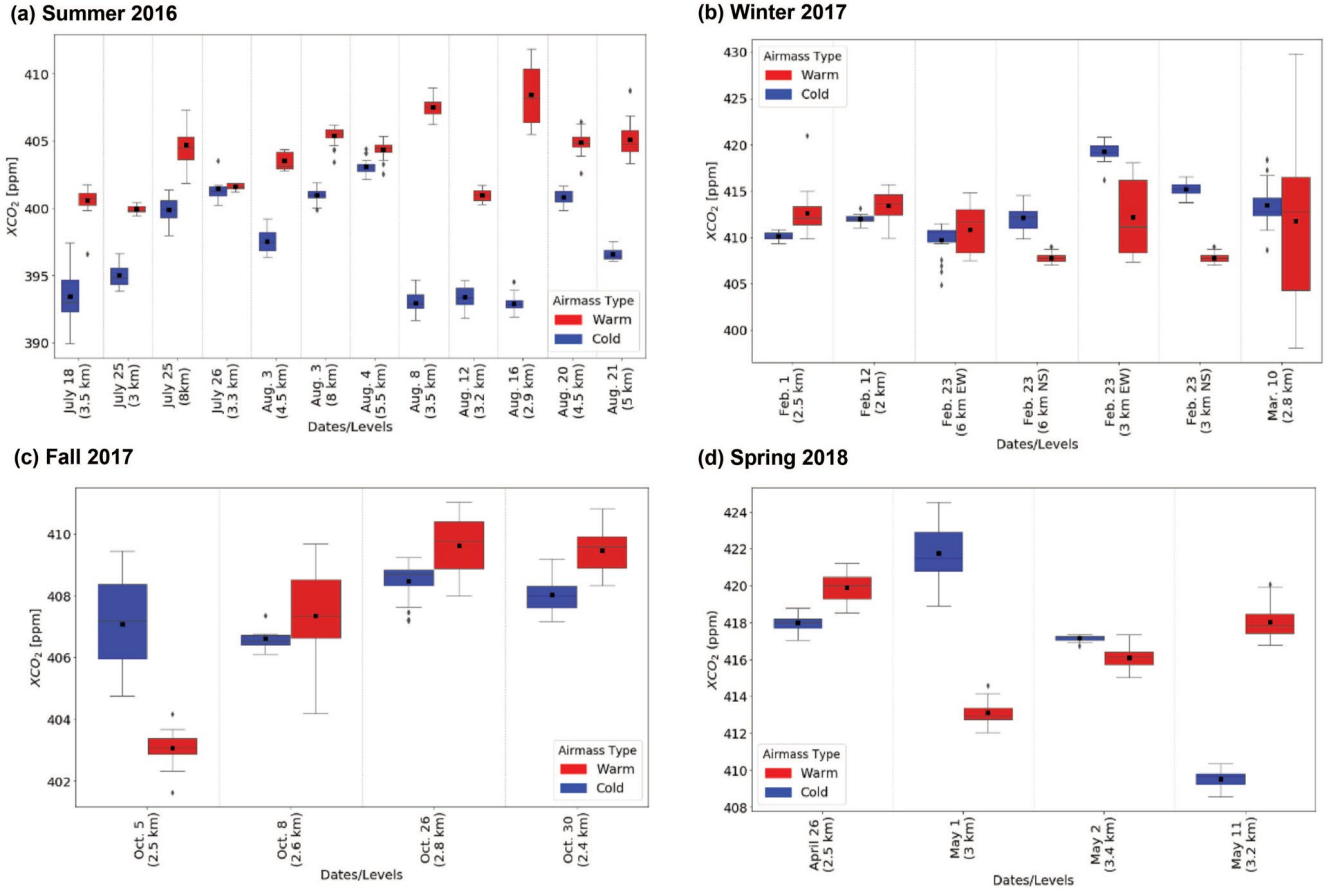
Seasonal summary of XCO_2_ variability in both warm and cold sectors of the frontal systems during the RFs of summer‐2016 (a), winter‐2017 (b), fall‐2017 (c), and spring‐2018 (d). Each red and blue pair of boxes corresponds to XCO_2_ in the furthest 0.5⁰ region of warm and cold sectors of the flight path, respectively. The box and whisker plots here and in other figures are represented in the same manner as Figure 1e.

Based on the method discussed in Section 3.2, a comprehensive summary of the XCO_2_ variability in the warm and cold sectors for all frontal RFs (Table S1 in Supporting Information S1) is first presented via box and whisker analyses (Figure 4). We note overview figures for all the frontal RFs (Table S1 in Supporting Information S1) during four field campaigns (summer‐2016: Figures S6–S18; winter 2017: Figures S19–S24; fall 2017: Figures S25–S28; and spring 2018: Figures S29–S31) are presented in Supporting Information S1. Also included is a box and whisker diagram that divides the flight track into 0.5⁰ latitude or longitude boxes and depicts the airmass type across the front. As reported in Supporting Information S1 (Table S1 and Figures S3–S31 in Supporting Information S1), the C‐130 altitudes (i.e., the MFLL platform) varied among different altitudes within a RF and among the RFs since the frontal RFs were designed in such a way that both remote sensing measurements of XCO_2_ and in situ measurements of CO_2_ were available in both warm and cold sectors in three different altitudes (e.g., ABL, lower free troposphere and upper free troposphere) over a wide region (400–800 km) while fulfilling the other aims of the RF missions of ACT‐America campaigns (e.g., the MFLL calibration, obtaining profiles of thermodynamic variables and GHGs and trace gases via en‐route ascent, descent, and targeted spirals).

However, while reporting XCO_2_ frontal contrasts, we compared the findings for the similar altitudes. Table S1 also provides some comprehensive information on each of the frontal crossing RFs including dates, regions, average flight altitudes for each level (red indicated the cases chosen for this work) including the rationale for the choice of the cases, weather conditions in the region of the flight, frontal flags, and comments if there was a region of enhanced XCO_2_ (where green represents the cases chosen for further XCO2 enhancement investigation), and finally, the reasoning for our choice of XCO_2_ enhancement cases.

These results (Figure 4 and Table S1 in Supporting Information S1) help explore the XCO_2_ spatial variability across frontal boundaries among different RFs in four seasons. One should note that the MFLL altitudes varied among the RFs, as shown in the *x*‐axis of Figure 4, so that case‐to‐case variability of XCO_2_ structures in both the warm and cold sectors should be interpreted with caution, as multiple altitudes are considered. As seen in Figure 4a, out of the 10 days, for a total of 12 cases, the average ΔXCO_2_ for summer was 8.5 ppm for 3‐km altitude flights, the largest average ΔXCO_2_ across all the seasons. The representative warm sector 0.5⁰ mean of XCO_2_ was always higher than that of the cold sector. In winter (see Figure 4b), there were a total of 7 cases, with several being the same day, just at different levels or sampling regions (Table S1 in Supporting Information S1). The average ΔXCO_2_ for 3‐km MSL MFLL observations in the winter was −7.4 ppm. In five of the seven cases, the warm sector XCO_2_ was less than that of the cold sector XCO_2_, while the rest of the cases mimicked summer findings.

The measurements of fall‐2017 and spring‐2018 provide a complex view into what happens to XCO_2_ during these transition seasons (Figures 4c and 4d). As broad latitudinal regions were sampled, different growth stages or dormancy in the plants depending on the region were observed. In the fall (see Figure 4c), all but 5 October 2017, out of the four MW and MA cases mimic the summer structure. The average ΔXCO_2_ for 2.8 km MSL altitude for fall‐2017 is 1.8 ppm. Again, this follows a pattern like summer but with a lesser ΔXCO_2_ magnitude. In spring‐2018, two out of the four in the MW and MA had higher values of XCO_2_ in the warm sector than in the cold sector. The average ΔXCO_2_ for spring‐2018 was 2.2 ppm. Thus, during these transition seasons (fall and spring), we did not find any clear signal in ΔXCO_2_. In general, there exist a spatial variability (south to north) in phenology in both fall and spring over the broad regions of the eastern US (Liu et al., 2021). For instance, the most productive regions are located in the north (Hilton et al., 2017; Miles et al., 2012). During the ACT‐America field campaigns, due to the fairly long north‐south transects (∼600 km) of the RFs. We indeed found phenology to be more variable in those two seasons. Thus, it is plausible that one portion of the frontal RF was more over a “spring like” atmosphere where plants are growing versus some areas that might have still been in dormancy leading to a more “winter like” atmosphere. Nevertheless, more analyses are required to determine the contribution of spatial variability of phenology to ΔXCO_2_, if any.

### 4.2 XCO_2_ Enhancements Near Fronts

A region of enhanced XCO_2_ near the frontal boundary was noted in several cases (Table S2 in Supporting Information S1). An example case illustrating the regions of ABL‐CO_2_ and XCO_2_ enhancements along flight track on 4 August 2016 is presented in Figure 2 (panels c and e). During this MW RF, aircraft transected a cold front in southwestern Nebraska at around 1800 UTC. A thin band of clouds was present in the region of the frontal boundary, with light precipitation. Results clearly indicate that both ABL‐CO_2_ and MFLL‐XCO_2_ were higher in the warm sector than the cold sector with varying degrees of ΔCO_2_ and ΔXCO_2_ due to the differences in their nature of sampling (in situ sampling within ABL only vs. columnar measurements of XCO_2_). The corresponding box‐and‐whisker analyses for both parameters are shown in Figures 2d and 2h. The XCO_2_ spatial variability in the warm sector also evinced an increasing trend from 94.5°W to 97°W toward the frontal boundary which we defined as an XCO_2_ enhancement and also confirms higher XCO_2_ spatial variability in the warm sector than the cold sector. Similar analyses were performed for all the frontal RFs which helped identify an enhancement in the MFLL‐XCO_2_ (Tables S1 and S2 in Supporting Information S1).

To explore the XCO_2_ enhancement feature, the region of enhancement was investigated using two metrics: the width of the XCO_2_ enhancement in the direction perpendicular to the front, and the magnitude of the XCO_2_ enhancement relative to the average XCO_2_ observed in the warm sector. Another example case (20 August 2016) was chosen to illustrate the XCO_2_ enhancement near the frontal boundary in the presence of an extensive cloud cover (Figure S2 in Supporting Information S1) and a summary of all the cases when such features were observed is presented in Table S2 in Supporting Information S1. A region of relatively higher XCO_2_ was noted in the vicinity of the front, whose location is identified using the Weather Prediction Center (WPC, 2022) surface analysis. The region of enhanced XCO_2_ extends between 33°N and 36.7°N (i.e., a horizontal distance of more than 300 km, marked by an oval shaped curve) along the flight track. The setup of the XCO_2_ enhancement along the track at 33°N was determined based on the box‐and‐whisker analyses when XCO_2_ value along the flight track in warm sector was at least 0.5 ppm higher than previous 0.5° box.

We also found that the warm sector XCO_2_ decreases gradually after the 36.7°N box (i.e., away from the front). When the XCO_2_ in this enhanced region was compared with the warm sector mean XCO_2_, the XCO_2_ enhancement was found to be around 2.7 ppm. One should note that frontal boundaries are often associated with the presence of optically thick clouds (e.g., Houze et al., 1981; Koch et al., 1995; Pal, Davis, Lauvaux, et al., 2020) yielding data gaps for airborne lidar measurements from an altitude above the clouds; the MFLL measurements during the frontal RFs of ACT‐America field campaigns were not an exception to this. Since this is the first time systematic MFLL measurements across frontal boundaries were performed, we were not aware of any algorithm to retrieve and fill the data gaps via interpolation in the XCO_2_ field. Consequently, we found it rather important to report the observed variability with data gaps. However, for all the analyses related to XCO_2_ enhancement presented here, we confirmed that a sufficient number of XCO_2_ samples (i.e., at least two box‐and‐whisker points) were available. Indeed, a large number of cases when sufficient number of XCO_2_ samples were not available to estimate XCO_2_ enhancement were excluded, although we observed XCO_2_ frontal contrasts in those cases (see Tables S1 and S2 in Supporting Information S1).

Thus, the estimated XCO_2_ enhancements reported here do suffer to some extent due to the missing observations in the regions of optically thick clouds along the track beneath the C‐130 platform. For a comprehensive description of the flagging method for the presence of clouds, readers are referred to Campbell et al. (2020). The average estimated width of the region of higher XCO_2_ is more than 300 km. We acknowledge that these measurement gaps will have an impact on the width and enhancement magnitude results presented. However, currently, we do not have enough quantitative information for an appropriate gap filling procedure and interpolation technique to obtain XCO_2_ at those locations.

Our analysis showed considerable case‐to‐case variability in the magnitude and extent of enhanced XCO_2_ features among the cases in addition to the cross‐frontal variability in XCO_2_ (Table S2 in Supporting Information S1). A common feature noted in all the cases was the presence of clouds at or near the flight. In all but two cases, precipitation was noted near the flight path. One should note that there were some frontal RF cases where we did not observe a clear signature of frontal enhancement, while for some other cases there exists some indication of enhancement in the vicinity of frontal boundaries, but we did not have an appropriate amount of XCO_2_ measurements in the warm sector to quantify the XCO_2_ enhancement features near the frontal boundary (see, Table S1 in Supporting Information S1). Thus, those were not reported in Table S2 in Supporting Information S1. Nevertheless, we believe that results pertaining to the unprecedented observations of both ΔXCO_2_ and the XCO_2_ enhancement features reported here will be valuable for both XCO_2_ remote sensing and modeling communities to resolve the issues related to “real” signal versus “noise” in XCO_2_ measurements in frontal environment.

### 4.3 Overall Model‐Data Intercomparison

**Figure 5:**
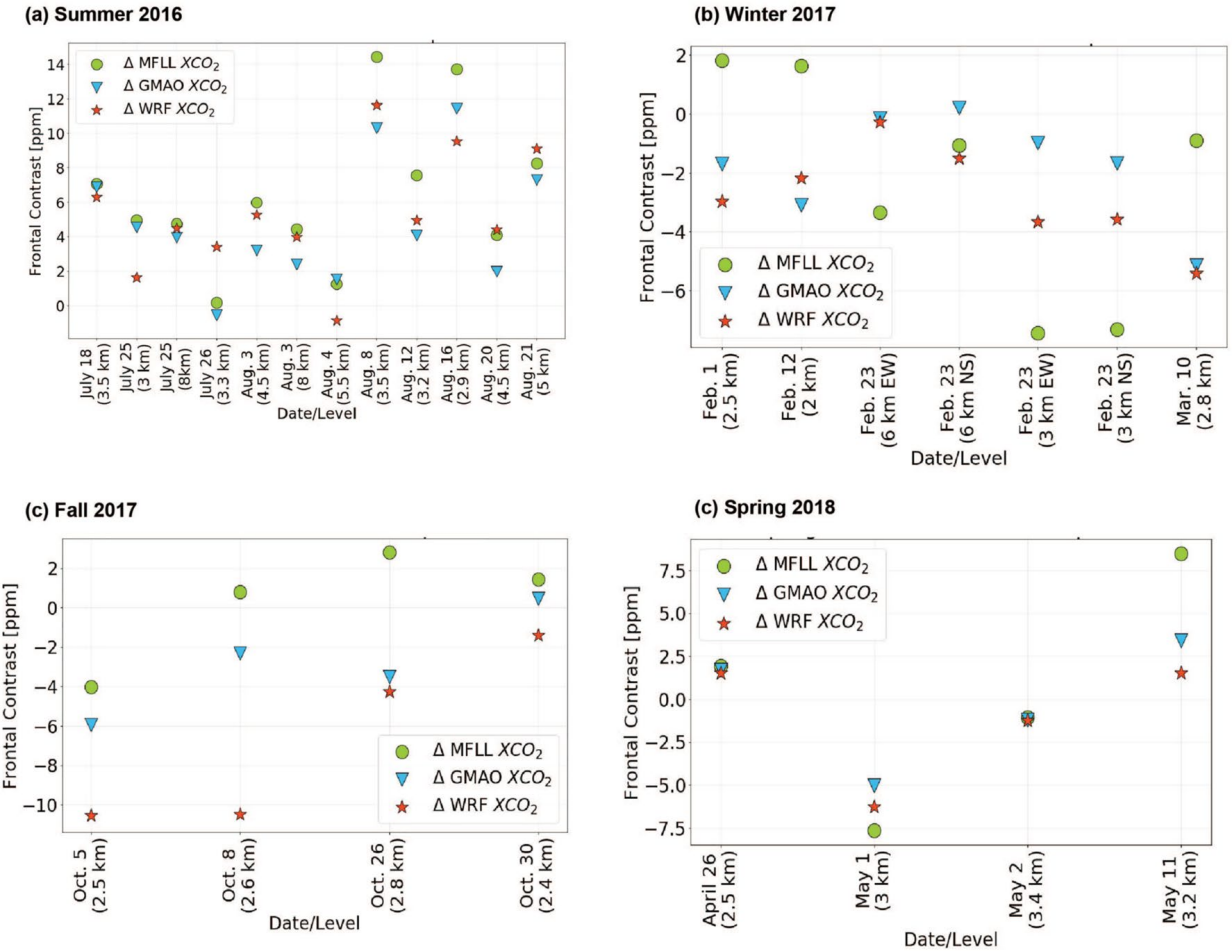
Summary of frontal contrast for each day calculated from the observed multifunctional fiber laser lidar (MFLL) XCO_2_ (green circles), and modeled XCO_2_ from the WRF‐Chem (red stars) and Global Modeling and Assimilation Office (GMAO) (blue triangles) for summer‐2016 (a), winter‐2017 (b), fall‐2017 (c), and spring‐2018 (d).

To better understand our ability, using models and observations for obtaining a consistent description of the impact of frontal passages on the XCO_2_ field, we investigated the three different estimates of ΔXCO_2_ (i.e., obtained via MFLL, GMAO, and WRF‐Chem) for all cases (Figure 5). There exists a tremendous amount of variability in the observed XCO_2_ frontal contrasts potentially due to (a) CO_2_ seasonal variability pertaining to the underlying fluxes associated with sources and sinks; (b) diverse nature of frontal passages and associated transport processes in both warm and cold sectors in four seasons; (c) XCO_2_ variability among different altitudes; (d) differences in regional scale CO_2_ fluxes within a region and among the three ACT‐America regions; (e) differences in meteorological characteristics associated with the frontal passages yielding myriad types of optically thick clouds, in particular, in the warm sectors within a RF and among the RFs.

**Figure 6:**
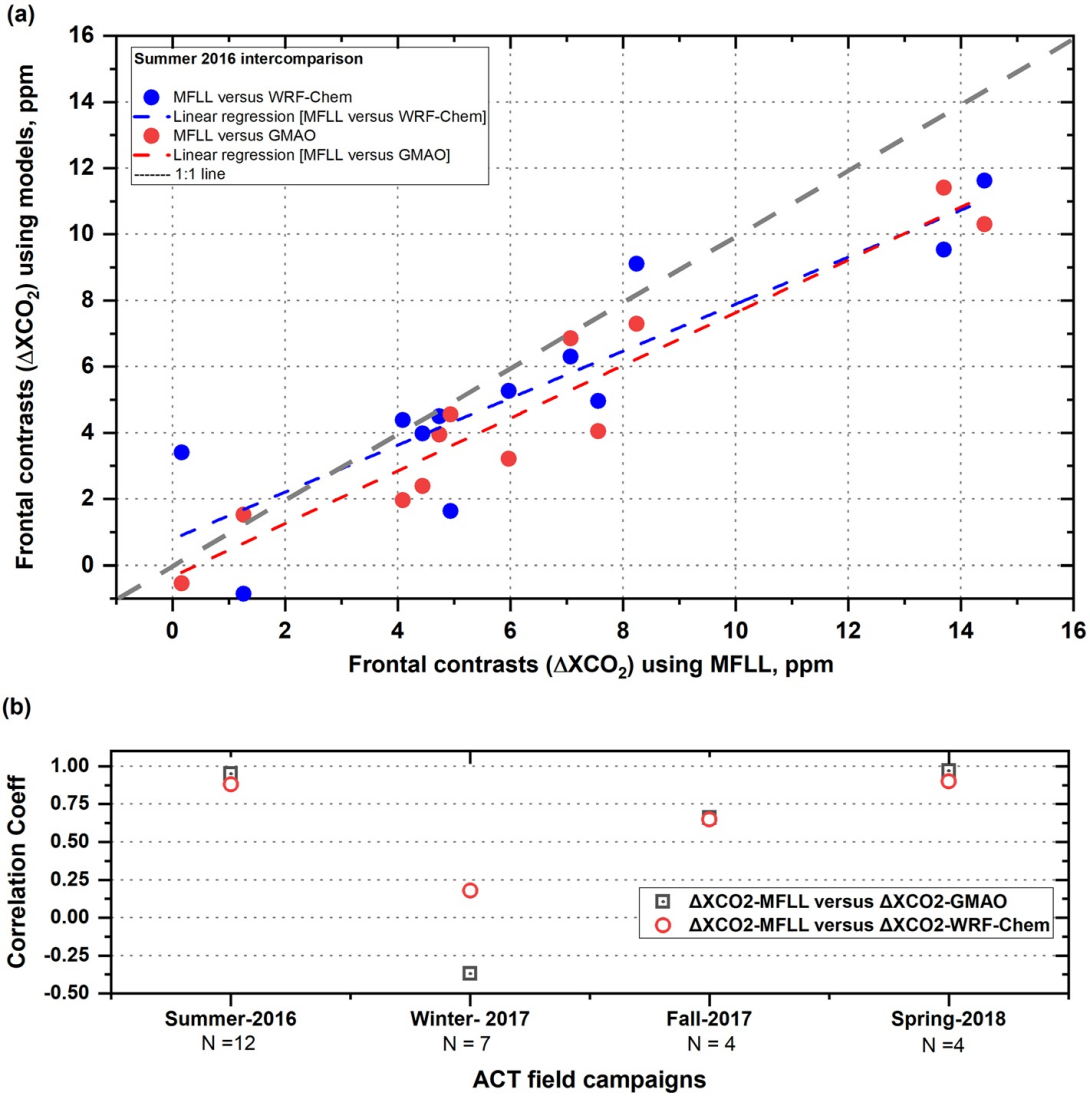
Linear regression analyses between ΔXCO_2_ obtained from multifunctional fiber laser lidar (MFLL) versus WRF‐Chem (blue solid circles and dotted line) and between MFLL versus Global Modeling and Assimilation Office (GMAO) (red solid circles and dotted line). Gray dotted line marks the 1:1 line (a). Correlation coefficients obtained from similar linear regression analyses between ΔXCO_2_ from MFLL and GMAO (gray squares) and between ΔXCO_2_ from MFLL and WRF‐Chem (red circles) for all four ACT‐America field campaigns. Number of frontal legs (N) are also indicated for each campaign along the *x*‐axis (b).

During summer‐2016, all three products of ΔXCO_2_ show similar sign (positive) though with varying magnitudes. However, clearly visible is that the case‐to‐case variability in ΔXCO_2_ was very similar in all three products. In particular, WRF‐XCO_2_ in summer yielded similar ΔXCO_2_ values as MFLL (though not identical) for the majority of the cases so that one could state that WRF‐Chem simulations reproduced the XCO_2_ frontal structures reasonably well and were able to “get the front right” without any ACT‐America observations except for a couple of instances. Additionally, ΔXCO_2_ variability among the cases in different seasons showed very similar tendency, except WRF‐Chem in fall‐2017. For instance, the linear regression analyses between ΔXCO_2_ obtained from MFLL versus GMAO, and MFLL versus WRF‐Chem for summer‐2016 cases yielded a correlation coefficient of 0.95 and 0.88, respectively (Figure 6a). Similar results for the other three seasons are presented in Figure 6b and Table S3 in Supporting Information S1.

Future work investigating the differences in XCO_2_ fields via assimilation versus no‐assimilation of ACT‐America data in the GMAO will help demonstrate the “value” in assimilating ACT‐America data. Similarly, exploring the impact of different transport on XCO_2_ field via comparing MFLL to CT will be another potential research topic but is beyond the scope of this work. We note that, recently, Weir et al. (2021) while illustrating the techniques for bias‐corrected surface fluxes derived from satellite observations and Peiro et al. (2022) while using ensembles of multiple atmospheric inversions characterized by different transport models, data assimilation algorithms, and prior fluxes, clearly found that the background for the curtain was of similar skill to CarbonTracker in reproducing independent evaluation data.

Additionally, as the signs of biological fluxes change across seasons, the ΔXCO_2_ variability also changed signs (Figure 5). For instance, during winter‐2017, all three products showed negative ΔXCO_2_ (i.e., lower XCO_2_ in the warm sector than the cold sector) except for the MFLL‐derived ΔXCO_2_ for first two cases. To investigate if there exist any seasonal biases among the three products, we explored seasonal means of ΔXCO_2_ obtained from MFLL, GMAO and WRF‐Chem (see Table S4 in Supporting Information S1). Clearly seen is that the ΔXCO_2_ values from all three products are similar with respect to magnitude and sign except WRF‐Chem in fall‐2017 when WRF‐ΔXCO_2_ was found to be much lower (−6.7 ppm) than both MFLL (0.3) and GMAO (−2.8). We note that winter‐2017 for MFLL is an outlier most likely due to the degradation of the telescope window coating. Correlation coefficients for both ΔXCO_2_ comparisons for winter‐2017 campaign (MFLL vs. GMAO and MFLL vs. WRF‐Chem) were found to be very low (0.2) and negative (−0.4), respectively (Figure 6b). Nevertheless, these results illustrate how challenging it is to observe and simulate these frontal differences in presence of significant case‐to‐case XCO_2_ variability within and among the seasons.

**Figure 7:**
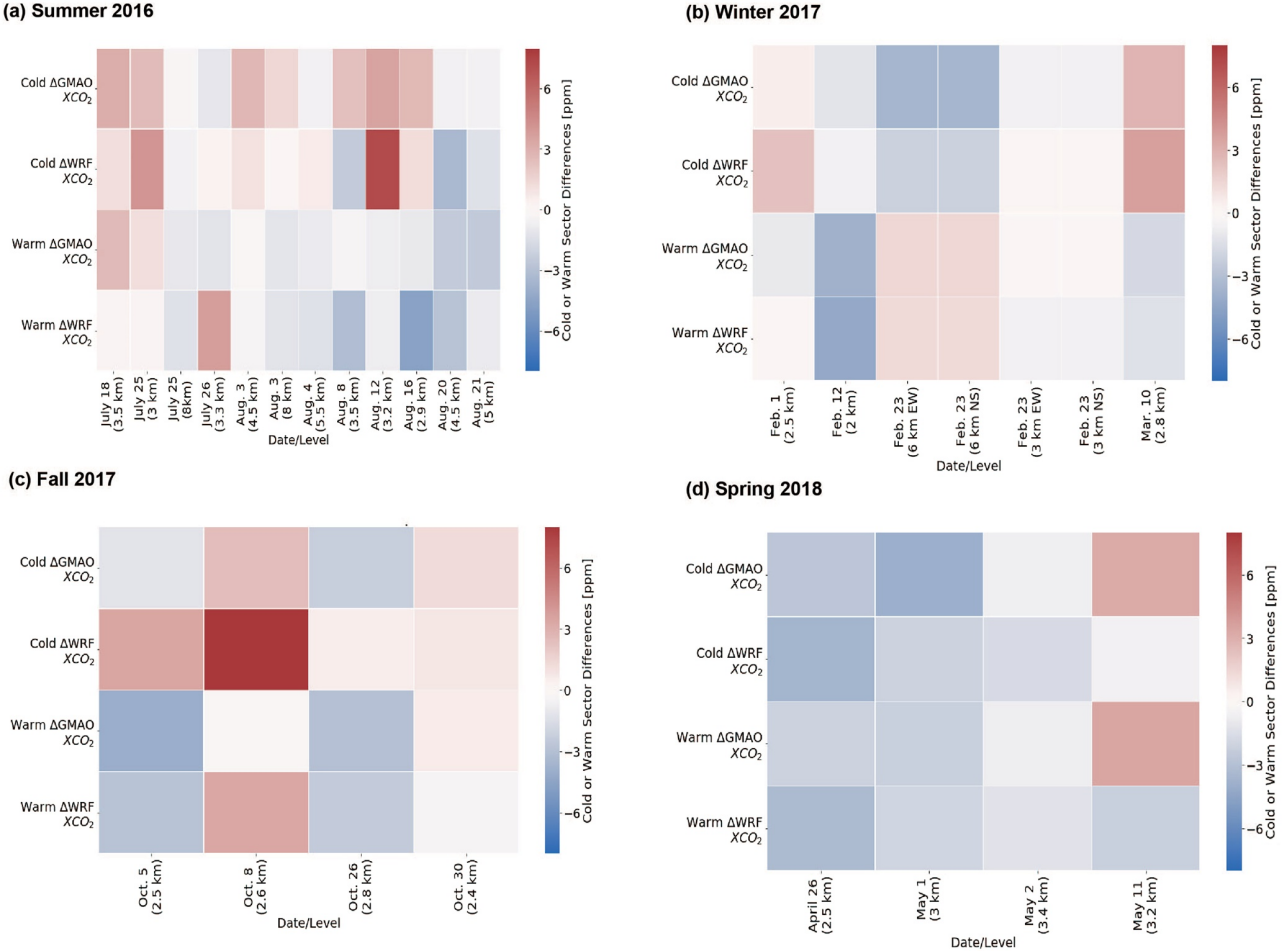
Summary of comparisons between multifunctional fiber laser lidar (MFLL) XCO_2_ with WRF‐Chem and GMAO XCO_2_ in both warm and cold sectors for summer‐2016 (a), winter‐2017 (b), fall‐2017 (c), and spring‐2018 (d). The red and blue colors represent positive and negative MDM, respectively. The lighter the colors, the smaller is the differences between in XCO_2_.

We also explored the MDM for XCO_2_ variability in both warm and cold sectors. For this purpose, the warm and cold sector XCO_2_ values for each of the three products were averaged, and then the averages were compared across products (Figure 7). During summer, for MFLL versus GMAO, average MDM for warm and cold sectors were −0.6 and 1.3 ppm, respectively while for MFLL versus WRF‐Chem average differences for warm and cold sectors were −1.0 and 0.7 ppm, respectively. Understanding how these differences compared to that of the cases in fair weather situations would allow for the identification of biases present in the models' ability to replicate XCO_2_ spatial variability in frontal environments.

Overall, we found broad similarities in XCO_2_ frontal contrast results obtained from MFLL, GMAO and WRF‐Chem though their degree of agreements varied among the cases and seasons. Previously, using ground‐based XCO_2_ measurements at TCCON site (Park Falls, Wisconsin), Keppel‐Aleks et al. (2012) found that XCO_2_ variability even at one location were primarily driven by combined impact of regional‐scale fluxes and atmospheric dynamics and also attributed the large‐scale gradients of XCO_2_ to synoptic‐scale meteorological processes. Using airborne in situ measurements of CO_2_ fields obtained during the ACT‐America RFs, Gerken et al. (2021) exploited the performance of both mesoscale (WRF‐Chem) and global scale (CT) models using identical surface fluxes and found reasonable agreement with observations in all four seasons. Additionally, for frontal RFs, Zhang et al. (2022) also found that OCO‐2 MIP models were capable of simulating observed CO_2_ frontal contrasts with varying degrees of success in summer and spring, and frequent underestimation of frontal contrasts in winter and autumn.

A straightforward conclusion cannot be made here regarding the potential of these models since the models were very different in those studies. In the context of MFLL measurements of XCO_2_ frontal contrasts and associated intercomparisons, several frontal RFs showed that three XCO_2_ products agree reasonably well in all seasons, in particular, potentially marks a great success of mesoscale models (here WRF‐Chem) which did not ingest any CO_2_ measurements. Nevertheless, we strongly believe that the reported MDMs could be cumulatively attributed to model resolution, assimilated data (for GMAO) and fluxes. One needs to perform more dedicated controlled experiments to understand the underlying processes and causes driving the differences which is beyond the scope of this work, but certainly remains an important future research topic.

### 4.4 Intercomparison Between MFLL and GMAO for Fair Weather Cases

To obtain a better understanding on the MDMs in XCO_2_, we performed an intercomparison analysis between the MFLL‐XCO_2_ and GMAO‐XCO_2_ for some selected fair weather RFs. Recently, Bell et al. (2020) also performed similar comparison but only for OCO‐2 underflight cases that were characterized with clear (<20% cloud coverage) and calm wind atmospheric conditions. One should note that within a series of recent ACT‐America research work, WRF‐Chem simulations were used to compare with aircraft observations for both fair weather and frontal RFs (e.g., Feng, Lauvaux, Davis, et al., 2019; Samaddar et al., 2021). Additionally, Bell et al. (2020) performed a comprehensive intercomparison between XCO_2_ fields obtained via MFLL and GMAO for extremely clear sky conditions (OCO‐2 underpass flights) and did not use WRF‐Chem simulations. Inclusion of additional results based on WRF‐Chem would definitely require many additional science questions to be addressed which remained the outside the scope of the present work. Thus, for brevity, we presented substantial new understanding of the earth's atmosphere using MFLL, WRF‐Chem and GMAO for frontal environment.

**Figure 8:**
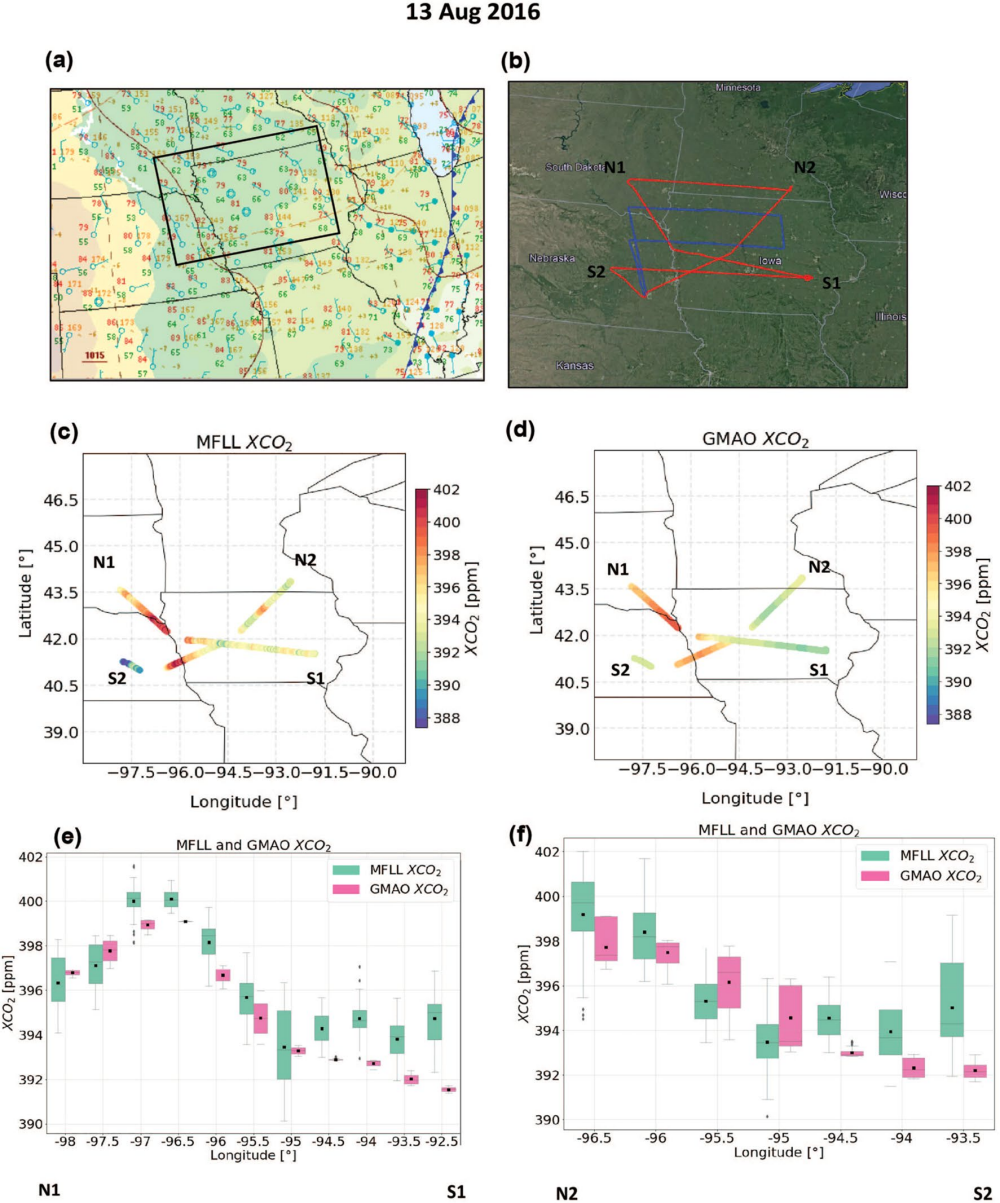
Intercomparison between MFLL‐XCO_2_ and GMAO‐XCO_2_ fields during a fair weather case in summer (13 August 2016). Surface map illustrating post‐frontal fair weather conditions (a) and flight tracks (red: C‐130, blue: B‐200, panel (b)) showing a typical ACT‐America fair weather research flight (here, box pattern under northerly flow as indicated on the surface map). MFLL measurements (3.3 km MSL column) along the diagonals (N1‐S1 and N2‐S2). XCO_2_ spatial variability obtained via MFLL (c) and GMAO (d) along with the box and whisker plot of the XCO_2_ along N1‐S1 binned into 0.5⁰ longitude segments for the MFLL‐XCO_2_ (green), and GMAO‐XCO_2_ (pink) (e); (f) same as (e) but along N2‐S2.

During a MW fair weather RF on 13 August 2016, the synoptic setup was characterized by post‐frontal condition with a relatively calm northerly flow (Figure 8a). The cloud physics lidar measurements also confirmed relatively clear sky conditions in the lower altitudes (Pal & Davis, 2021; Pal, Davis, Pauly, et al., 2020). The RF was designed as a box pattern with horizontal legs that sampled east to west and west to east in the boundary layer, as well as the diagonal legs (3.3 km MSL) across the box that sampled the FT when MFLL measurements were available (Figure 8b). These types of box pattern RFs were conducted during the ACT‐America campaigns to estimate CO_2_ and CH_4_ fluxes (Davis et al., 2021).

Figure 8 evinces that there exists XCO_2_ spatial variability though without a region of enhanced XCO_2_ as was observed during the frontal RFs. Both MFLL and GMAO retrievals show the presence of a clearly visible west‐to‐east XCO_2_ difference (∼4–5 ppm in 3.3 km MSL column with XCO_2_ values of 398–400 ppm and 394–395 and in the western and eastern parts of the RF tracks, respectively). The GMAO‐XCO_2_ and MFLL‐XCO_2_ along both tracks (N1‐S1 and N2‐S2) agrees reasonably well except the eastern most portion of the RF where MFLL‐XCO_2_ were 2 ppm higher than the GMAO‐XCO_2_. However, overall MDMs along N1‐S1 and N2‐S2 legs were found to be −1.2 and −0.8 ppm, respectively which are much lower than the MDMs obtained during the frontal RFs over the same region. For instance, for frontal RF on 12 August 2016 over the same region, we obtained MDMs (GMAO vs. MFLL) of more than 3.5 ppm (Figure 7a).

An overview of the MDMs for 3 selected fair weather cases in summer‐2016 are shown in Supporting Information S1 (see Table S5 and Figure S18 in Supporting Information S1). By comparing the MFLL‐XCO_2_ and GMAO‐XCO_2,_ the average difference was found to be ∼−0.1 ppm, though with some case‐to‐case variability. Overall, there remains some moderate XCO_2_ spatial variability during fair weather cases, but the differences between the GMAO‐XCO_2_ and the MFLL‐XCO_2_ remain much smaller to what was found for the frontal RFs (see, Table S5 and Figure 7a). We also found that XCO_2_ spatial variability during frontal cases were higher than during the fair weather cases. Cumulatively, these results suggest that higher XCO_2_ spatial variability yielded also higher MDMs in XCO_2_ and vice‐versa.

## 5 Discussion

### 5.1 XCO_2_ Frontal Contrasts

The 27 cases examined confirm that the XCO_2_ field is substantially affected by frontal passages. Based on all three products, a clear pattern was observed in the summer with higher XCO_2_ in the warm sector than in the cold sector. Using in situ measurements in the ABL and FT across frontal boundaries, Pal, Davis, Lauvaux, et al. (2020) also found the identical sign in the frontal contrasts in observed CO_2_ spatial variability (i.e., higher CO_2_ in the warm sector than the cold sector in both ABL and FT), which supports the assertion that the MFLL can identify these patterns. While segregating the frontal contrast results in summer by altitudes, we found that ΔXCO_2_ were in general larger in magnitude for low altitudes than for the higher altitudes. For instance, for summer cases, mean ΔXCO_2_ obtained for the altitude ranges of 2.5–3.5, 4.5–3.5, and 8.0 km were 8.0, 4.9 and 4.5 ppm, respectively. One should note that we did not have enough ΔXCO_2_ measurements in different altitudes across frontal boundaries in other seasons to determine such altitude dependence feature of ΔXCO_2_, if any (see Table S1 in Supporting Information S1).

The ΔXCO_2_ in fall were found to be of similar sign (i.e., positive) as was found for the summer cases except for 5 October 2017. However, the ΔXCO_2_ in summer were larger in magnitude compared to fall, most likely due to larger spatial variability in underlying fluxes in summer. One noticeable feature for the 5 October 2017 case was the presence of optically thick clouds in the cold sector unlike the other cases which might have yielded higher XCO_2_ in the cold sector than the warm sector yielding contrasting sign in ΔXCO_2_ (Figure S4 in Supporting Information S1). These cloud covers most likely yielded reduced photosynthetic uptake as was reported in a number of past studies (e.g., Chan et al., 2004; Hu et al., 2020; Lee et al., 2012, 2015). We also found a very high XCO_2_ enhancement (12.2 ppm over a span of around 55 km) in the vicinity of the frontal boundary for this case (Table S2 in Supporting Information S1). To estimate the XCO_2_ enhancement around the frontal boundary, we considered warm sector samples obtained between 38.5 and 39.0°N latitude range (i.e., as evinced in the far right corner of panel f) as a reference. Presence of an optically thick low‐level cloud band and associated precipitation field around the frontal boundary in central PA (see panels d and b, respectively) resulted in gaps in MFLL measurements. Notwithstanding, we obtained sufficient XCO_2_ measurements around the frontal boundary to estimate the XCO_2_ enhancement as reported in Table S2 in Supporting Information S1.

For winter cases, two of the seven RFs saw higher values of XCO_2_ in the warm sector, with the other five seeing higher XCO_2_ in the cold sector than in the warm sector indicating a contrasting feature to summer. The winter MW cases are all from 23 February 2017, from two levels, 3 and 6 km, and are centered around a low‐pressure center with the north‐south flights crossing the warm front and the east‐west flights crossing the cold front. In all these cases, the cold sector MFLL‐XCO_2_ are higher than the warm sector. The spring has several interesting cases, including observations made on 26 April 2018 and 11 May 2018 (see Supporting Information S1). The 26 April 2018 case potentially sampled two frontal boundaries. Two sharp enhancements are seen in the XCO_2_ field and the dewpoint field (see Figure S29 in Supporting Information S1). The additional panel in Figure S29 in Supporting Information S1 illustrate the locations of two frontal boundaries and associated results of XCO_2_ spatial variability. For this purpose, we analyzed dewpoint temperature spatial variability obtained along the C‐130 track marking the locations of frontal boundaries. The 11 May 2018 case has a sharp enhancement in XCO_2_ in the warm sector but was not further investigated due to its location with respect to the remaining warm sector. However, this case provides a clear distinction between the warm and cold sectors with a ΔXCO_2_ of 8.5 ppm. Radar measurements confirmed that there were no precipitation events in the northern VA region around the cold front boundary though there were some clouds as appeared in the GOES‐16 imagery (Figure S5 in Supporting Information S1). The surface analyses suggested that this was almost a stationary front.

During several RFs, some extraordinary variations in XCO_2_ were observed presumably due to prevailing meteorological conditions including location of frontal boundaries and upwind fluxes in the two sectors. The observed XCO_2_ fields in both warm and cold sectors were largely affected by the diverse fluxes in the upwind of both sectors while frontal lifting, cloudiness, and ABL depth variability near the frontal boundary contributed to the XCO_2_ enhancement frequently observed during the RFs. Two potential factors that become crucial in the MDMs in the XCO_2_ fields including XCO_2_ frontal contrasts are (a) uncertainties in XCO_2_ retrievals in highly variable water vapor environment as mentioned earlier, and (b) atmospheric variability including key meteorological conditions, cloudiness, radiation, horizontal wind, and ABL depths. Nevertheless, some example results reported here strictly confirmed that extraordinary variations in MFLL XCO_2_ field (e.g., spatial variability across frontal boundary, enhancement near the frontal boundary, etc.) were similar when compared to in situ CO_2_ variability in the ABL from similar environments.

The results reported have important implications for carbon cycle research toward establishing relationship among XCO_2_ and ABL‐CO_2_ fields and surface fluxes and north‐south hemispheric gradient in frontal environment which will further guide improved flux estimations and future satellite missions. We note Keppel‐Aleks et al. (2011) found that during synoptically active environments, XCO_2_ variability is mainly caused by large‐scale eddy‐driven disturbances of the meridional gradient. Recently, Cui et al. (2021) developed a source‐receptor relationship between net ecosystem exchange (NEE) and atmospheric CO_2_ variability along flight tracks using the Lagrangian particle dispersion models and confirmed upwind fluxes in frontal sectors mainly drives a major part of the CO_2_ frontal contrasts.

### 5.2 XCO_2_ Enhancements

The region of enhanced XCO_2_ at the frontal boundary is a complex feature, with several possible explanations for an occurrence. One common feature between all frontal boundary enhancement cases is the presence of extensive cloud cover in the vicinity, or at the region of XCO_2_ enhancement. Chan et al. (2004) noted that the cloud cover in the vicinity of a front reduces the photosynthetic uptake of the corn crops in the MW, resulting in CO_2_ gradients in frontal regions. This decrease in photosynthetic uptake would lead to an increase in CO_2_ near the given region of clouds. This would likely be most prominent in the summer when the photosynthetic uptake is already large. However, based on the region and season, this was also observed in other seasons. We note a comprehensive understanding on the impact of diverse cloud covers near frontal boundaries on photosynthesis uptake and XCO_2_ would definitely need further investigation on the time scale of response for flux into atmospheric concentrations. Using a weather‐biosphere‐online‐coupled model, Hu et al. (2021) investigated the development of CO_2_ bands in the ABL at the frontal boundary. Similar investigation for XCO_2_ enhancement at the frontal boundary using numerical models is a worthwhile topic. Within this work, we documented substantial, new observational findings on XCO_2_ spatial variability in frontal environment, and we defined metrics that can be used in future studies including numerical simulations of the transport of GHGs by midlatitude cyclones which is indeed required for accurate inverse CO_2_ flux estimation (e.g., Baker et al., 2022).

Using hourly averaged measurements of O_3_ from the Air Quality System database, Hegarty et al. (2007) reported that synoptically inactive regimes could lead to the collection of O_3_ in a region until a synoptic system transports the pooled O_3_ out of the region. Recently, Samaddar et al. (2021) attributed the elevated CO_2_ mole fractions along the frontal boundary to continental biogenic CO_2_ fluxes. Samaddar et al. (2021) noted that horizontal advection was most dominant near the frontal boundary and positively impacted the amount of CO_2_ in the warm sector. While horizontal advection appears to be the main driver in the XCO_2_ enhancement near the frontal boundary, the effects of clouds and precipitation need to be analyzed further to fully understand the effects these occurrences have on the variability of the XCO_2_ as was performed in Hu et al. (2021). Additionally, because all seven cases of frontal XCO_2_ enhancements were associated with the presence of clouds near the frontal boundaries, and four of the seven cases were associated with convection, this is a characteristic feature that warrants future investigations. We used available radar measurements to identify the frontal boundaries associated with presence of strong precipitation bands as shown in Supporting Information S1.

### 5.3 Comparison Among MFLL, GMAO and WRF

The objective behind comparing the MFLL‐XCO_2_ with the WRF‐XCO_2_ and GMAO‐XCO_2_ was to identify consistency and quantify differences among the three XCO_2_ products in frontal environment. The ΔXCO_2_ results obtained from GMAO retrievals were found to agree reasonably well with both sign and magnitudes of MFLL observations of ΔXCO_2_ for the frontal RFs presented here. Previously, Bell et al. (2020) also found good agreement between GMAO retrieval and MFLL observations of XCO_2_ spatial variability over the similar region during fair weather RFs of ACT‐America in four seasons. Thus, results reported here and in Bell et al. (2020) cumulatively demonstrate the potential of GMAO retrievals of XCO_2_ for both frontal and fair weather environments over land. Additionally, an error source not discussed by Campbell et al. (2020) is the potential for biases related to the increased humidity ahead of a frontal boundary, leading to errors in the spectral broadening due to water vapor. Sensitivities of the MFLL‐XCO_2_ retrieval to inputs like meteorology and spectroscopy were studied in some detail in Bell et al. (2020) though only for clear sky conditions. During frontal passages, a large amount of humidity changes occurred, in particular, within warm sector and high contrasts in humidity between the warm and cold sectors while model‐simulated water vapor fields were used for MFLL retrievals.

For all the MFLL measurements presented here, every effort was made to minimize bias due to water vapor. The position of the lasers on the absorption feature were selected to minimize influence from water vapor, but it is not possible to completely eliminate the influence of the continuum. The MFLL retrievals use the latest spectroscopy and state‐of‐the‐art reanalysis data, namely MERRA‐2, to correct for water vapor. Additionally, the instrument has been calibrated against in situ measurements and was shown to maintain the calibration to within 0.5 ppm over different seasons under different atmospheric conditions, indicating that the residual influence of water vapor bias is small compared to the features being discussed here. The largest biases would be seen where there is large variation of water vapor that is not well captured by the reanalysis products due to the coarser spatial resolution versus the lidar measurement. As spectroscopy and the reanalysis products continue to improve, we believe that XCO_2_ retrieval biases will be further reduced. Eventually, in the future, it will be feasible to combine direct lidar measurements of the water vapor with the CO_2_ measurements which would significantly reduce the potential for biases due to water vapor in highly variable cases.

Further research in this direction for those RFs would be very beneficial to examine the biases that might be introduced into MFLL by water vapor gradients in frontal environment. For instance, while exploring XCO_2_ spatial variability in fair‐weather conditions, Bell et al. (2020) investigated the dependence of XCO_2_ retrieval on variable water vapor spectroscopy and variable meteorological conditions. It was demonstrated that the MFLL retrieval of XCO_2_ field is particularly sensitive to water vapor via both the meteorology and spectroscopy. In general, XCO_2_ retrieval from MFLL measurements strongly depend on water vapor profile which is used to estimate differential optical depth of water vapor (i.e., Δ𝜏_H20_). For the MFLL measurements reported here, we used model‐derived (MERRA‐2) water vapor profiles. The water vapor absorption cross‐sectional profiles were obtained at the model grid points and times based on their meteorological profiles including height, temperature, pressure, and humidity. Additionally, during ACT‐America RFs, the MFLL used two OFF wavelengths at 50 pm to either side of the ON line (i.e., single CO_2_ absorption line centered at 1,571.112 nm, yielding two off lines at 1,571.062 and 1,571.162 nm, referred to as S and *L*, respectively). Since offline wavelength (*L*) has higher water vapor sensitivity, we only used the shorter wavelength for calculating differential optical depths (Lin et al., 2020). Previously, Refaat et al. (2015, 2020) though for 2‐μm IPDA lidar, and Dobler et al. (2013) and Bell et al. (2020) for MFLL, performed a number of sensitivity analyses to determine the impact of water vapor profiles on the XCO_2_ retrievals. However, there exist no systematic investigations reporting sensitivity of XCO_2_ retrievals on two contrasting water vapor environments as often present in warm and cold sectors of a frontal boundary, and under highly variable water vapor regimes in the boundary layer, in particular, in the warm sector.

Based on the past results reported in Campbell et al. (2020) and Bell et al. (2020), we found it to be plausible that highly variable water vapor regimes along the frontal RF tracks in four seasons would also impact XCO_2_ retrievals differently in two contrasting water vapor environments (i.e., warm, and cold sectors), consequently, on ΔXCO_2_. In particular, biases are primarily driven by uncertainty in the true column water vapor versus the reanalysis product column water vapor as interpolated for use in the retrieval as well as any uncertainties in the spectroscopy used. Although we are aware of potential for bias due to uncertainties in water vapor and spectroscopy and have made every effort to minimize that bias using the best in class reanalysis products and latest spectroscopy, it remains an extremely complex task to accurately quantify the bias.

For 26 April 2018, as seen in Figure 7d, WRF‐Chem can replicate the frontal contrast by 0.4 ppm difference from the MFLL. However, the WRF‐XCO_2_ was underestimated in warm sector by −3.3 ppm compared to MFLL‐XCO_2_ and the cold sector by −3.7 ppm. A table yielding the key results on frontal contrasts is reported in Supporting Information S1 (see Table S6 in Supporting Information S1). Thus, this case serves as an example of how WRF‐Chem simulations can accurately replicate how much the XCO_2_ field changed across the boundary but under or overestimated the magnitude of the entire XCO_2_ field. The differences in WRF‐XCO_2_ and GMAO‐XCO_2_ are likely due in part to the models' data ingested. The GMAO assimilates data from the in situ CO_2_ measurements collected during each flight. Notwithstanding, this study identified the MDMs with respect to observational findings on the XCO_2_ spatial variability across frontal systems in four seasons.

In general, XCO_2_ field in the warm sector for most of the cases was found to be more variable than that in the cold sector, in particular, in summer and winter (see the spread of the box and whiskers in Figures 4a and 4b and results presented in Figures S2c, S6a, S7a, S8a, and S13a in Supporting Information S1). We speculate that higher XCO_2_ variability in the warm sector than the cold sector could be attributed to the dominant variability in CO_2_ flux in warm sector than in the cold sector. For both spring and fall, such tendencies were not observed (Figures 4c and 4d). Future studies using either observations or model results (or both) of underlying CO_2_ fluxes will help demonstrating the differences in CO_2_ fluxes across the frontal boundary and their impact on XCO_2_ frontal contrasts, if any.

Besides the agreements of XCO_2_ variability using MFLL observations and model results, we also noted some differences among these products in all four seasons. For some cases, models tend to evince small spread in the box‐and‐whisker plots (see the boxes in Figure 2, S3, S4, and S5 in Supporting Information S1) while for some other cases we note that simulated XCO_2_ yielded larger variability than observations (see, Figures S3, S8, S9, and S16 in Supporting Information S1). As mentioned previously, for the results presented here, the spatial resolutions in models and observations vary significantly, it will be interesting to examine the impact of different model resolutions on the differences between the observed and simulated XCO_2_ variability. In a nutshell, how XCO_2_ variability and associated front‐relevant metrics are related to model spatial resolution coarseness needs more research, as there are examples of GMAO (with coarser resolution than WRF‐Chem) showing larger variability than WRF‐Chem as well as MFLL observations. Another notable difference was found in the linear regression analyses between ΔXCO_2_ from MFLL and GMAO and between ΔXCO_2_ from MFLL and WRF‐Chem (Figure 6). In particular, the winter correlation is low (MFLL vs. WRF‐Chem) and even negative (MFLL vs. GMAO) which most likely occurred due to both lower number of samples and poor performance of MFLL in winter‐2017 campaign due to a known window coating degradation.

Within this work, for the first time, we systematically used the GMAO‐XCO_2_ fields obtained during multiple frontal RFs in four seasons over land as we introduced a system that establishes calibration standards for OCO‐2 and lidar retrievals based on in situ data from the ACT‐America campaign. The system assimilates the in situ data into NASA's Goddard Earth Observing System (GEOS) to produce high‐resolution, two‐dimensional transects of CO_2_ along the flight path which we refer to as curtains. Excluding the ability to sample the entire atmosphere at once, any such analysis must make assumptions about the connection of measurements at different places and times to a given retrieval. We chose to use the GEOS general circulation model forced by meteorology from its data assimilation system because their scientific merits are extensively documented. Furthermore, in data‐rich environments, the assimilated curtains approach a field constrained by data alone (Bell et al., 2020).

### 5.4 Frontal Boundary

The frontal flags allowed for obtaining much more precise frontal boundary location rather than estimating the corresponding frontal boundary location with the flight time and location via surface map analysis. We used the flight level frontal flags for most of the analyses. However, when frontal flags were missing along the FT legs (i.e., at the MFLL altitudes), we used ABL frontal flags. While these flags have proven to be very useful to this research, there is some uncertainty associated with the frontal flag due to some erroneous measurements of meteorological variables (e.g., water vapor mixing ratio measurements on C‐130 platform in summer‐2016) or few gaps in measurements across the frontal boundaries during conditions like the presence of thick clouds or a gust front. Because of this uncertainty, for 9 out of the 27 cases, the frontal flag was not available for the given altitude or time of the flight. However, in these cases, the frontal boundary was investigated by analyzing the temperature and dewpoints along the flight track and at the given altitude and estimating the frontal boundary using the location of a significant change in those variables.

Due to the nature of the MFLL and the vertical slope of frontal boundaries, identifying the location of the frontal boundary at all altitudes is crucial for identifying the XCO_2_ field in each sector. For instance, as illustrated in the conceptual diagram (Figure 1) and exemplified in Figures 2 and 4 (see Supporting Information S1 for other cases), slope of the frontal boundary (in altitude) yielded a northward shift in the frontal boundary in height. However, in the vicinity of the frontal boundary itself, the MFLL samples both the warm and cold sectors due to the slanted nature of the frontal boundary. Thus, in those 9 cases where the frontal flag was not used, there may be more uncertainty with what sector is being sampled near the frontal boundary itself. However, the possible error from our frontal boundary estimation appears to be negligible, as ΔXCO_2_ were mainly estimated from the 0.5° sectors away from the frontal boundary.

## 6 Summary, Conclusions, and Outlook

We reported the XCO_2_ spatial variability due to the passages of frontal systems in all four seasons using the MFLL‐retrived ‐partial column of XCO_2_ measurements below the aircraft. The MFLL provides a unique perspective in investigating the varying patterns in XCO_2_. For instance, measurements and simulations suggested that XCO_2_ field was more heterogeneous in the warm sector compared to the cold sector in all four seasons, as was previously found for ABL‐CO_2_ fields in frontal sectors (Pal et al., 2020). We also explored how the differences of the three retrievals vary within and among the seasons. Overall, for the first time, we showed how the magnitude and sign of frontal contrasts (i.e., ΔXCO_2_) vary by season and illustrated how challenging it is to observe and simulate these frontal differences in presence of significant case‐to‐case ΔXCO_2_ variability.

We demonstrated the ability to use the MFLL to identify the varied structure of XCO_2_ across frontal boundaries. Based on the GMAO‐XCO_2_, we found that the XCO_2_ variability in summer (winter) had the most straightforward pattern, with the warm (cold) sector consistently higher than the cold (warm) sector XCO_2_. The two transition seasons (fall and spring) did not show a consistent pattern in XCO_2_ variability with respect to frontal boundaries. We hypothesized that XCO_2_ depends highly on fluxes and significant spatial variability in phenology over land present during these two transition seasons most likely caused the observed XCO_2_ frontal contrasts. Despite a lack of a clear pattern in fall, and spring, one clear conclusion is that all of the cases yielded the effect of a frontal passage in the warm sector versus cold sector XCO_2_ fields with consistent sign of ΔXCO_2_ in all three products.

While comparing the XCO_2_ retrievals from MFLL, GMAO and WRF‐Chem, and exploring how close those three products were to each other, we found that the MDMs were much smaller compared to the frontal contrasts (i.e., ΔXCO_2_) though with some variability among the cases (Figures 6 and 8). Additionally, all three products showed very similar tendency in XCO_2_ spatial variability across fronts as confirmed via the linear regression analyses and associated results of high correlation coefficients. Finally, the magnitude and sign of ΔXCO_2_ from the three products were found to be very similar in different seasons except WRF‐XCO_2_ in fall‐2017. For instance, for summer‐2016, average ΔXCO_2_ from MFLL, WRF‐Chem and GMAO were found to be 6.4, 5.3, and 4.7 ppm, respectively. All three products showed the presence of an enhanced region of XCO_2_ in the vicinity of frontal boundary though with varying magnitudes in different seasons. Cumulatively, the results confirmed that the models can interpret XCO_2_ variability across frontal boundaries in all seasons. Our results also illustrated the typical frontal signals in XCO_2_ spatial variability in four seasons so that it helps obtain some insights into the fact what satellite‐based sensors should be seeing for ΔXCO_2_.

Bell et al. (2020) showed the latitudinal gradients in the XCO_2_ using OCO‐2, MFLL, and GMAO‐curtains for some selected days under fair weather conditions. Our analyses provide similar analyses between MFLL‐XCO_2_ and GMAO‐XCO_2_ fields but for synoptically active environments. Our work indicates larger MDM of the XCO_2_ fields for synoptically active environments compared to fair weather cases. These results provide some information on the ΔXCO_2_ that could be expected from OCO‐2 measurements. Also, in general, XCO_2_ variability in fair weather is of high interests for the OCO‐2/3 communities to resolve the issue involved in XCO_2_ spatial variability versus instrument noise. In future, we will expand the research to understand better what causes the models' biases and the correlation between the clouds/precipitation and the region of enhanced XCO_2_ at the frontal boundary.

## Conflict of Interest

The authors declare no conflicts of interest relevant to this study.

## Supporting information

Supporting Information S1

## Data Availability

The latest version of ACT‐America data (both in situ and MFLL observations) is publicly available at the Oak Ridge National Laboratory (ORNL) Distributed Active Archive Center (https://daac.ornl.gov/actamerica). The MFLL weighting function and Lite files are available in Lin et al. (2022a, 2022b), respectively on ORNL data archive (https://doi.org/10.3334/ORNLDAAC/1891 and https://doi.org/10.3334/ORNLDAAC/1892, respectively). Users need to create an account and can download datasets without any fees. ACT‐America airborne and tower GHG observations are also integrated into the NOAA/GML ObsPack data products (Masarie et al., 2014, https://www.esrl.noaa.gov/gmd/ccgg/obspack/). A comprehensive description of the datasets is available in Wei et al. (2021). The surface charts were obtained from NOAA's WPC analyses available at https://www.wpc.ncep.noaa.gov/archives/web_pages/sfc/sfc_archive.php.
