# Supplementary material for: Airborne Lidar Measurements of XCO2 in Synoptically Active Environment and Associated Comparisons With Numerical Simulations
Source: J Geophys Res Atmos. 2022 Aug 17;127(16):e2021JD035664. doi: 10.1029/2021JD035664 (PMC9786724; doi:10.1029/2021JD035664)
Supplement: Supplementary file 1 — Supporting Information S1 [file JGRD-127-e2021JD035664-s001.docx]

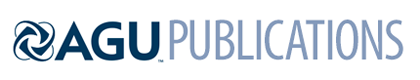


*Journal of Geophysical Research: Atmospheres*

Supporting Information for

**Airborne Lidar Measurements of XCO_2_ in Synoptically Active Environment and Associated Comparisons with Numerical Simulations**

*Samantha Walley^1^, Sandip Pal^1^, Joel F. Campbell^2^, Emily Bell^3^, Brad Weir^4,5^, Sha Feng^6,7^, Thomas Lauvaux^7,8^, David Baker^3^, Nathan Blume^9^, Jeremy Dobler^9^, Wayne Erxleben^10^, Tai-Fang Fan^11^, Bing Lin^2^, Doug McGregor^9^, Michael D. Obland^2^, Chris O'Dell^3^, Kenneth J. Davis^7,12^*

*^1^Department of Geosciences, Atmospheric Science Division, Texas Tech University, ^2^NASA Langley Research Center (LaRC), ^3^Colorado State University, ^4^Universities Space Research Association, ^5^NASA Goddard Space Flight Center, ^6^Atmospheric Sciences and Global Change Division, Pacific Northwest National Laboratory, ^7^Department of Meteorology and Atmospheric Science, The Pennsylvania State University, ^8^LSCE - IPSL, CEA Saclay, France, ^9^Spectral Sensor Solutions LLC, ^10^L3 Harris Technologies, ^11^Science System and Application, Inc, , ^12^Earth and Environmental Systems Institute, The Pennsylvania State University*

**Contents of this file**

Text S1-S2

Tables S1 to S6

Figures S1 to S30

**Introduction**

In this document, we presented observations of XCO_2_ across various frontal boundaries using the Multi-Functional Fiber Laser Lidar (MFLL) that was deployed during the ACT-America flight campaigns. Data were collected during four different seasons: summer 2016, fall 2017, winter 2017, and spring 2018. Three regions were also investigated in the eastern half of the US including: The Mid-West (MW), the Mid-Atlantic (MA), and the South (SO). To identify biases that models might have in replicating the spatial variability in XCO_2_ caused by the passage of a synoptic front, XCO_2_ simulations from WRF-Chem and Global Modeling and Assimilation Office (GMAO) were compared with the MFLL observed XCO_2_. an overview of the XCO­_2_ spatial variability obtained from all three retrievals (MFLL, WRF-Chem and GMAO) for all the cases is presented in chronological order in the SI (Figs. S2-S31).

**S1. Case studies for model data intercomparisons**

**Summer Case: 04 Aug 2016**

A case study from each season was chosen to illustrate the model-data intercomparison framework. For brevity, we have presented one case for summer-2016 campaign (4 Aug 2016) in Fig. 2 and other three cases are briefly outlined here (see Figs. S3-S5). For 4 Aug 2016 case, the MFLL XCO_2_ frontal contrast yielded higher XCO_2_ in the warm sector versus lower XCO_2_ in the cold sector, with an XCO_2_ frontal contrast of 1.3 ppm (Fig. 2). The enhancement near the frontal boundary was found to be ~ 1.6 ppm higher than the warm sector's mean. This enhancement was mainly observed in the region of the clouds and precipitation based on satellite imagery obtained during the flight. The enhanced XCO_2_ band spanned an estimated 1⁰ longitude or about 85 km around the frontal boundary (Figs. 5c-d). The in-situ measurement in the ABL also confirmed an enhanced region of CO_2_ around the cold front boundary (Figs. 2c-d).

When comparing the frontal contrasts among the three products we found that the MFLL- ΔXCO_2_, WRF-ΔXCO_2_ and GMAO-ΔXCO_2_ were ~ 1.3, -0.9, and 1.5 ppm, respectively (Fig. 2h). When comparing the entire warm and cold sectors for the MFLL-XCO_2_ and WRF-XCO_2_, the model slightly overestimated the XCO_2_ in the cold sector and slightly underestimated the XCO_2_ in the warm sector which most likely caused the different sign in WRF-ΔXCO_2_ than the other two products (MFLL and GMAO). The GMAO-XCO_2_ averages for the warm and cold sectors were lower than those observed by the MFLL, with an average difference of -0.9 ppm in the warm sector and -0.5 ppm in the cold sector when the MFLL-XCO_2_ was compared to GMAO-XCO_2_. Overall, the GMAO-XCO_2_ agreed better with the MFLL-XCO_2_ than the WRF-XCO_2_ did for this case.

**Winter Case: 12 Feb 2017**

During the frontal RF on 12 February 2017 (i.e., ACT-America winter campaign), the C-130 aircraft sampled a cold front boundary, as identified by the WPC surface map, and no precipitation was indicated by radar imagery (Fig. S3) along the Southeastern/Northeastern border of Texas and Oklahoma (Fig. S3). Satellite imagery indicates clouds presented across northern/central eastern Texas. An almost 30⁰C drop in dewpoint temperatures occurred slightly north of the Texas/Oklahoma border, with a significant drop in temperature closer to the border at 2 km MSL flight altitude.

The frontal contrast in the observed XCO_2_ was 1.6 ppm. A region of elevated XCO_2_ was observed near the frontal boundary, with an average value of around 5.5 ppm larger than the warm sector average. This regional enhancement spanned about 110 km in distance (Fig. S3). In situ observations of CO_2_ in the boundary layer also indicated a substantial enhancement in the region of the front (Pal et al., 2020). This region of enhanced XCO_2_ was collocated with the area of clouds based on satellite imagery. Across MFLL, WRF-Chem, and the GMAO curtain, there were significant differences in frontal contrast: the MFLL frontal contrast was 1.6 ppm, with the WRF-Chem and GMAO frontal contrast being -2.2 ppm and -3.1 ppm (Fig. S3). In the cold sector, the difference between the average WRF-Chem XCO_2_ and the average MFLL XCO_2_ was -0.6 ppm, while the difference between the average GMAO XCO_2_ and the average MFLL XCO_2_ was -1.2 ppm. In the warm sector, the difference between the average WRF-Chem XCO_2_ and the average MFLL XCO_2_ was -4.2 ppm, while the difference between the average GMAO XCO_2_ and the average MFLL XCO_2_ was -3.8 ppm.

**Fall Case: 5 Oct 2017**

For this case (RF on 5 October 2017) over the MA region, a stationary frontal boundary was observed across central PA (see Fig. S4). Widespread clouds and light precipitation dominated the flight region, which spanned from southern NY to central VA (Figs. 9b-d). The dewpoint measurements indicated a substantial decrease along the north-central border of PA and NY. The cold sector had larger values of XCO_2_, with the frontal contrast near -4.0 ppm. This case is in contrast with the rest of the Fall 2017 cases, being the only case where the cold sector XCO_2_ was higher than the warm sector. The region of enhancement near the frontal boundary, spanning only a half of a degree in latitude, resulted in an XCO_2_ value for the region averaging 12.2 ppm higher than that of the mean of the entire warm sector.

There were significant differences between the MFLL retrieved frontal contrast of -4.0 and the WRF-Chem simulated, and GMAO assimilated frontal contrasts of -10.5 ppm and -5.9 ppm, respectively. In the cold and warm sectors, the GMAO assimilated XCO_2_ differs from the MFLL XCO_2_ by -1.2 ppm and -4.0 ppm, respectively. WRF-Chem XCO­_2_ underestimated the XCO_2_ average in the warm sector by -2.8 ppm and overestimated the XCO_2_ average in the cold sector by 3.5 ppm.

**Spring Case:11 May 2018**

This flight sampled a cold front in central PA around 1600 UTC, as depicted by Fig. S5. Clouds were present northwest of VA, with precipitation occurring to the Northwest of the frontal boundary (Fig. S5). There was a sharp gradient in dewpoint temperature at the 2.5 km MSL level where the XCO_2_ measurements were collected. The frontal contrast in MFLL XCO_2_ was 8.5 ppm, with a region of higher XCO_2_ just to the south of the sharp drop in dewpoint temperatures. A region of higher XCO_2_ following the frontal boundary within the warm sector was also observed but not further investigated due to insufficient data in the warm sector of the frontal boundary that is not included in the area of the enhanced XCO_2_. The WRF-Chem simulated and GMAO assimilated XCO_2_ differed from the MFLL observed frontal contrast by a difference of 1.5 ppm and 3.4 ppm, respectively (Fig. S5). The WRF-Chem underestimated both by -2.2 ppm and -0.6 ppm when looking into the warm and cold sector XCO_2_ differences. The assimilated XCO_2_ from the GMAO differs from the MFLL XCO_2_ in both warm and cold sectors by 3.5 ppm and 3.2 ppm, respectively.

**S2. An overview for model data intercomparisons for all cases**

For brevity, in this section, we presented an *overview figure* for all the frontal research flights (Table S1) in chronological order (summer-2016: Figs. S6-S18; winter 2017: Figs. S19-S24; fall 2017: Figs. S25-S28; and spring 2018: Figs. S29-S31). Also included is a box and whisker diagram that divides the flight track into 0.5⁰ latitude or longitude boxes and depicts the airmass type across the front.

***Table S1:*** *Description of each of the frontal crossing RFs during summer 2016, fall 2017, winter 2017, and spring 2018 ACT-America campaigns, including dates, regions, average flight altitudes for each level (red indicated the cases chosen for this work) including the rationale for the choice of the cases, weather conditions in the region of the flight, and comments if there was a region of enhanced XCO_2_ (where green represents the cases chosen for further XCO_2_ enhancement investigation), and finally, the reasoning for our choice of XCO_2_ enhancement cases. Cold front is abbreviated by CF, stationary front by SF, warm front by WF, enhancement by ENH, frontal boundary by FB, Insufficient data by ID, sufficient data by SD, Not investigated by NI and frontal flag by FF. An overview figure for each case is also included here in the SI (see Figs. S2-S31). Summer cases: 18 Jul - 21 Aug 2016; Winter cases: 1 Feb – 10 Mar 2017; Fall cases: 5 Oct- 3 Nov 2017; and Spring cases: 22 Apr – 20 May 2018.*

| **Date** | **Region** | **Altitude, km MSL** | **Reason for case selection for this study** | **Weather Conditions** | **FB Determi-nation Method** | **Region of ENH** | **Reasons for ENH case choice** |
| --- | --- | --- | --- | --- | --- | --- | --- |
| 18 Jul 2016 | MA | 8.0 | ID | CF located south of Lake Erie; Partly Cloudy | NI | N/A | N/A |
|  |  | **3.2** | SD but with gaps |  | FF | No ENH | N/A |
| 19 Jul 2016 | MA | 8.0 | Complex flight pattern/ ID | SF located in E VA; few clouds | NI | N/A | N/A |
|  |  | 3.4 | ID |  | FF | N/A | N/A |
| 25 Jul 2016 | MA | **7.8** | SD but with gaps | CF through central OH; Scattered clouds and storms across the flight path | FF | No ENH | N/A |
|  |  | **3.0** | SD |  | FF | No ENH | N/A |
| 26 Jul 2016 | MA | **5.8** | SD | CF located in N VA; Scattered clouds at FB | FF | No ENH | N/A |
|  |  | 3.3 | Complex flight pattern and frontal location |  | NI | N/A | N/A |
| 3 Aug 2016 | MW | **8.0** | SD | CF in SE WY; Clouds and light precipitation at FB | FF | ENH in the warm sector. | Not at FB |
|  |  | **4.0** | SD |  | FF | Small ENH in warm sector. | Not at FB |
| 4 Aug 2016 | MW | 8.0 | ID | CF in central NE; Clouds and potential precipitation at the FB | NI | N/A | N/A |
|  |  | **5.5** | SD |  | ABL FF | Sharp ENH at FB | ENH at FB |
| 8 Aug 2016 | MW | 8.0 | ID | SF located in near N OK; Clouds and precipitation in the region | NI | N/A | N/A |
|  |  | **3.2** | SD |  | ABL FF | No ENH | N/A |
| 12 Aug 2016 | MW | 8.0 | ID | SF in NW MO; Precipitation and heavy clouds present | NI | N/A | N/A |
|  |  | **3.2** | SD but with small gaps |  | FF | ENH at the FB with brief drop | ENH at FB |
| 16 Aug 2016 | MW-SO | **3.0** | SD | CF in SE OK; Clouds and precipitation at FB | FF | Sharp ENH at FB | ID in the warm sector |
| 20 Aug 2016 | SO | **4.5** | SD but with small gaps | CF in N AR; Heavy clouds and precipitation in the region | FF | Sharp ENH at FB | ENH at FB |
|  |  | 2.5 | Large model data disagreement/ uncertainty source unidentified |  | FF | Sharp ENH at FB | Case eliminated from dataset |
| 21 Aug 2016 | SO | **5.2** | Limited but SD | SF in N LA; Clouds and precipitation in the region | Dewpoint | Small ENH in warm sector. | Not at FB |
|  |  | 2.5 | Complex flight pattern/ ID |  | NI | N/A | N/A |
| 1 Feb 2017 | SO | 5.0 | ID | CF across central AR; Limited clouds | NI | N/A | N/A |
|  |  | **2.3** | SD with small gaps |  | FT FF | ENH in both the cold and warm sector | No ENH at the FB |
| 12 Feb 2017 | SO | 6.3 | ID | CF located in Central E TX; Clouds located near FB | NI | N/A | N/A |
|  |  | **2.0** | SD |  | FF | ENH t at the FB | ENH at FB |
| 20 Feb 2017 | MW | N/A | C-130 Down | Strong N/S oriented cold front across NE | NI | N/A | N/A |
| 23 Feb 2017 | MW | **6.0** (N/S) | SD | CF in W OK (E/W Flights) and warm front in S KA (N/S Flight); Low pressure center in central CO limited clouds in the region | Dewpoint | No ENH | N/A |
|  |  | **6.0** (E/W) | SD |  | FF | No ENH | N/A |
|  |  | **3.5** (N/S) | SD |  | Dewpoint | Slight ENH in the cold sector | Not at FB |
|  |  | **3.5** (E/W) | SD |  | FF | No ENH | N/A |
| 7 Mar 2017 | MA | 2.5 | ID | Strong CF across S MI and N OH; widespread clouds and precipitation | NI | N/A | N/A |
|  |  | 1.0 | Altitude too low |  | NI | N/A | N/A |
| 10 Mar 2017 | MA | **2.8** | SD | CF extending off the N NC Coast; Clouds in the region | Dewpoint | No ENH | N/A |
|  |  | 1.0 | Altitude too low |  | NI | N/A | N/A |
| 5 Oct 2017 | MA | **2.5** | SD | SF in Central PA; Widespread clouds and precipitation | ABL FF | Strong ENH at the FB | ENH at FB |
| 8 Oct 2017 | MA | 7.0 | ID | CF in SW PA; widespread clouds | NI | N/A | N/A |
|  |  | **2.5** | SD |  | FF | ENH in the warm sector | Not at FB |
| 10 Oct 2017 | MA | 7.0 | Does not cross FB | CF to the south of the region, as well as to the north; Precipitation and clouds in region | NI | N/A | N/A |
| 11 Oct 2017 | MA | 2.4 | Large MDM and surface CO_2_ obs. | CF located in E KY and TN; Clouds and scattered showers associated behind FB throughout flight track | FF | ENH in the cold sector | Case eliminated from dataset |
| 21 Oct 2017 | MW | 7.0 | ID | CF extending S through N Plains; Storms located W of cold front with widespread clouds throughout region | NI | N/A | N/A |
|  |  | 3.8 | ID in cold sector |  | NI | N/A | N/A |
|  |  | 2.3 | ID |  | NI | N/A | N/A |
| 26 Oct 2017 | MW | 6.9 | ID | CF in NW MO; Limited clouds | NI | N/A | N/A |
|  |  | **2.5** | SD |  | FF | ENH at the FB | Bell structure in flight pattern across FB |
| 26 Oct 2017 | MW-SO | 6.2 | Does not cross FB | CF across SE OK; Clouds and potentially light precipitation in northern portion of flight track | NI | N/A | N/A |
|  |  | 4.0 | ID |  | NI | N/A | N/A |
|  |  | **2.2** | SD |  | FF | ENH in the warm sector | Not at FB |
| 2 Nov 2017 | SO | N/A | Complex flight pattern | SF located across the SE with a dryline located in central TX and OK; Widespread clouds along SF | NI | N/A | N/A |
| 3 Nov 2017 | SO | N/A | Complex flight pattern | Low pressure center near Shreveport, LA; C-130 crosses CF with widespread clouds | NI | N/A | N/A |
| 18 Apr 2018 | SO | N/A | C-130 Down | CF from the NW moves S | NI | N/A | N/A |
| 22 Apr 2018 | SO | N/A | C-130 Down | CF thru Shreveport, LA with eastern passage | NI | N/A | N/A |
| 26 Apr 2018 | MW | 6.8 | ID | CF across central KA; Limited precipitation and widespread clouds | NI | N/A | N/A |
|  |  | **2.5** | SD |  | FF | two ENHs in the warm sector | Complex frontal pattern. |
| 1 May 2018 | MW | 8.0 | ID | CF located in NE IA; Widespread clouds across flight track with precipitation south of FB | NI | N/A | N/A |
|  |  | **2.8** | SD |  | Dewpoint | No ENH | N/A |
| 2 May 2018 | MW | 6.8 | Does not cross FB | CF across NW MO; Precipitation and clouds to the NW of the FB | NI | N/A | N/A |
|  |  | **3.2** | SD |  | FF | ENH near FB with gaps | SD in the warm sector. |
| 4 May 2018 | MW-MA | N/A | Complex flight pattern | CF crossing in central IN | NI | N/A | N/A |
| 11 May 2018 | MA | 7.0 | ID | SF located in NE VA; limited clouds | NI | N/A | N/A |
|  |  | **3.1** | SD |  | FF | ENH in the warm sector | No ENH at FB |
| 16 May 2018 | MA | 2.5 | Large MDM/ID in cold sector | SF located in NE VA; widespread clouds and precipitation | NI | N/A | N/A |
| 18 May 2018 | MA | 6.8 | ID | SF in central VA | NI | N/A | N/A |
|  |  | 2.5 | ID |  | NI | N/A | N/A |
| 20 May 2018 | MA | N/A | Complex flight pattern | CF located along East Coast | NI | N/A | N/A |

***Table S2:*** *Summary of frontal enhancement cases with information regarding date, flight level, the difference between the enhanced region's mean XCO_2_ and the average of the warm sector XCO_2_, the distance of the enhanced region, and a summary of the meteorological condition present in each case.*

| **Date** | **Flight altiude (km MSL)** | **Region** | **XCO_2_ enhancement**  **(ppm)** | **Distance**  **(km)** | **Meteorological**  **Conditions** |
| --- | --- | --- | --- | --- | --- |
| 4 Aug 2016 | 5.5 | MW | 1.6 | 85 | Narrowband of clouds near- frontal boundary. Radar indicated precipitation occurring at the boundary. |
| 12 Aug 2016 | 3.0 | MW | 2.0 | 165 | Clouds and light precipitation were occurring in the region of the frontal boundary. |
| 20 Aug 2016 | 4.5 | SO | 2.7 | 320 | Clouds and precipitation occurring with some convection in central Texas. |
| 12 Feb 2017 | 2.0 | SO | 5.5 | 110 | Band of clouds located south of the frontal boundary, but no radar indicated precipitation occurring during the flight time. |
| 5 Oct 2017 | 2.5 | MA | 12.2 | 55 | A wide swath of clouds across the entire flight track with precipitation occurring in central Pennsylvania. |
| 2 May 2018 | 3.0 | MW | 1.2 | 55 | Clouds present to the north and south of the frontal boundary, but no precipitation occurring near the flight path. |

***Table S3****: Results of the linear regression among XCO_2_ frontal contrasts obtained with MFLL versus WRF-Chem and MFLL versus GMAO.*

| **Season** | **Correlation coefficient: ΔXCO_2_ obtained from MFLL versus GMAO** | **Correlation coefficient: ΔXCO_2_ obtained from MFLL versus WRF-Chem** | **Number of frontal legs** |
| --- | --- | --- | --- |
| Summer-2016 | 0.95 | 0.88 | 12 |
| Winter- 2017 | -0.37 | 0.18 | 7 |
| Fall-2017 | 0.66 | 0.65 | 4 |
| Spring-2018 | 0.97 | 0.90 | 4 |

***Table S4:*** *Summary of the seasonal means of XCO_2_ frontal contrasts obtained from three retrievals (MFLL, WRF-Chem and GMAO) during four field campaigns.*

| **Season** | **Seasonal means of ΔXCO_2_ (ppm) obtained from** | | |
| --- | --- | --- | --- |
|  | **MFLL** | **WRF-Chem** | **GMAO** |
| Summer-2016 | 6.4 | 5.3 | 4.7 |
| Winter- 2017 | -2.4 | -0.3 | -1.8 |
| Fall-2017 | 0.3 | -6.7 | -2.8 |
| Spring-2018 | 0.4 | -1.1 | -0.3 |

***Table S5****: An overview of the model-data differences in XCO_2_ (GMAO versus MFLL) obtained during three fair weather cases of summer 2016 ACT-America field campaign.*

| **Date** | **Flight altitude**  **(Km MSL)** | **GMAO and MFLL difference in XCO_2_, ppm** | | **Overall model-data difference in XCO_2_ (ppm)** | **Weather Conditions** |
| --- | --- | --- | --- | --- | --- |
|  |  | **NW to SE leg** | **SW to NE leg** | **Average of both legs** | **Over the entire flight domain** |
| 13 Aug 2016 | 3.3 | -1.2 | -0.8 | -1.0 | Located in the cold sector, behind a cold frontal passage with calm winds with clear to partly cloudy skies. |
| 14 Aug 2016 | 3.8 | 2.5 | 0.8 | 1.7 | Located near a high-pressure center, with clear skies and calm winds |
| 22 Aug 2016 | 3.3 | 0.1 | -0.6 | -0.3 | Located N of a stationary boundary with clear to cloudy skies and calm winds. |

***Table S6****: An overview of the f*rontal contrasts obtained from MFLL, WRF-Chem, and the GMAO retrievals for all cases. See Fig. 6 and related discussion in text.

| **Date (YYYY-MM-DD) and altitude, km MSL** | **MFLL ΔXCO_2_** | **WRF ΔXCO_2_** | **GMAO ΔXCO_2_** |
| --- | --- | --- | --- |
| **Summer 2016 Research Flights** |  |  |  |
| 2016-07-18 and 3.5 | 7.1 | 6.3 | 6.9 |
| 2016-07-25 and 3 | 4.9 | 1.6 | 4.6 |
| 2016-07-25 and 8 | 4.7 | 4.5 | 3.9 |
| 2016-07-26 and 3.3 | 0.2 | 3.4 | -0.5 |
| 2016-08-03 and 4.5 | 5.9 | 5.3 | 3.2 |
| 2016-08-03 and 8 | 4.4 | 3.9 | 2.4 |
| 2016-08-04 and 5.5 | 1.3 | -0.8 | 1.5 |
| 2016-08-08 and 3.5 | 14.4 | 11.6 | 10.3 |
| 2016-08-12 and 3.2 | 7.6 | 4.9 | 4.1 |
| 2016-08-16 and 2.9 | 13.7 | 9.5 | 11.4 |
| 2016-08-20 and 4.5 | 4.1 | 4.4 | 1.9 |
| 2016-08-21 and 5 | 8.2 | 9.1 | 7.3 |
| **Winter 2017 Research Flights** |  |  |  |
| 2017-02-01 and 2.5 | 1.8 | -2.9 | -1.7 |
| 2017-02-12 and 2 | 1.6 | -2.2 | -3.1 |
| 2017-02-23 and 6 (N-S) | -3.3 | -0.3 | -0.1 |
| 2017-02-23 and 6 (E-W) | -1.1 | -1.5 | 0.2 |
| 2017-02-23 and 3 (N-S) | -7.4 | -3.7 | -0.90 |
| 2017-02-23 and 3 (E-W) | -7.3 | -3.6 | -1.7 |
| 2017-03-10 and 2.1 | -0.9 | -5.4 | -5.1 |
| **Fall 2017 Research Flights** |  |  |  |
| 2017-10-05 and 2.5 | -4.0 | -10.5 | -5.9 |
| 2017-10-08 and 2.6 | 0.8 | -10.5 | -2.3 |
| 2017-10-26 and 2.8 | 2.8 | -4.3 | -3.5 |
| 2017-10-30 and 2.4 | 1.4 | -1.4 | 0.5 |
| **Spring 2018 Research Flights** |  |  |  |
| 2018-04-28 and 2.5 | 1.9 | 1.5 | 1.7 |
| 2018-05-01 and 3 | -7.7 | -6.3 | -5.0 |
| 2018-05-02 and 3.4 | -1.1 | -1.2 | -1.2 |
| 2018-05-11 and 3.2 | 8.5 | 1.5 | 3.4 |

**
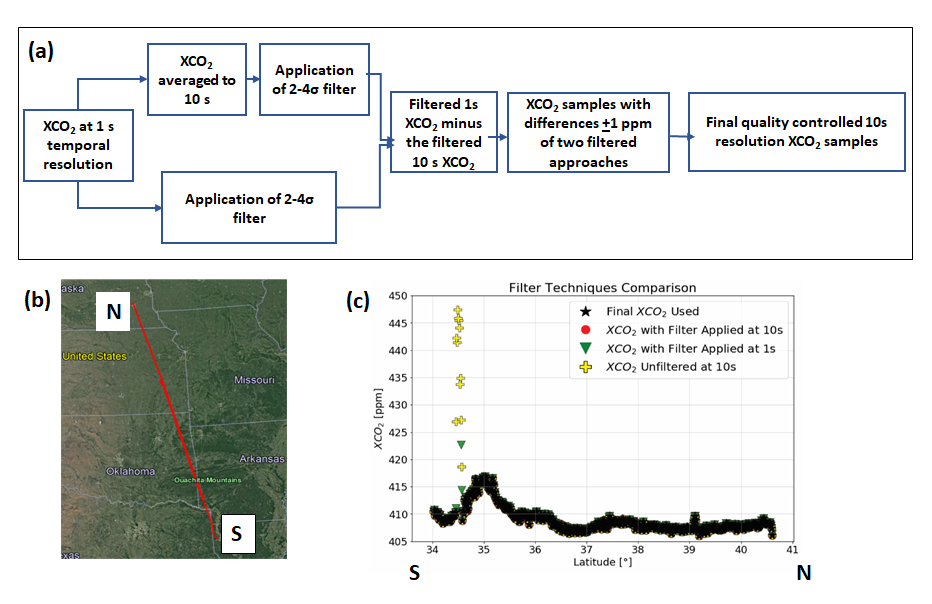
**

***Figure S1****: A flowchart illustrating the different steps involved in the additional quality control method applied to the MFLL dataset obtained from ORNL data archive (a). An example frontal crossing RF track in the MW region on 30 Oct 2017 showing the flight path of the C-130; N and S denote the northern and southern most point of the C-130 track, respectively (b) and example result illustrating the filtering technique used for the MFLL data before the sigma filter is applied (yellow plus) is compared with the filtered data at 1 s (green triangle), the 10 s averaged filtered data (red circles, mostly in the background of stars), and finally the filtered data used for analysis (black stars) (c).*

***
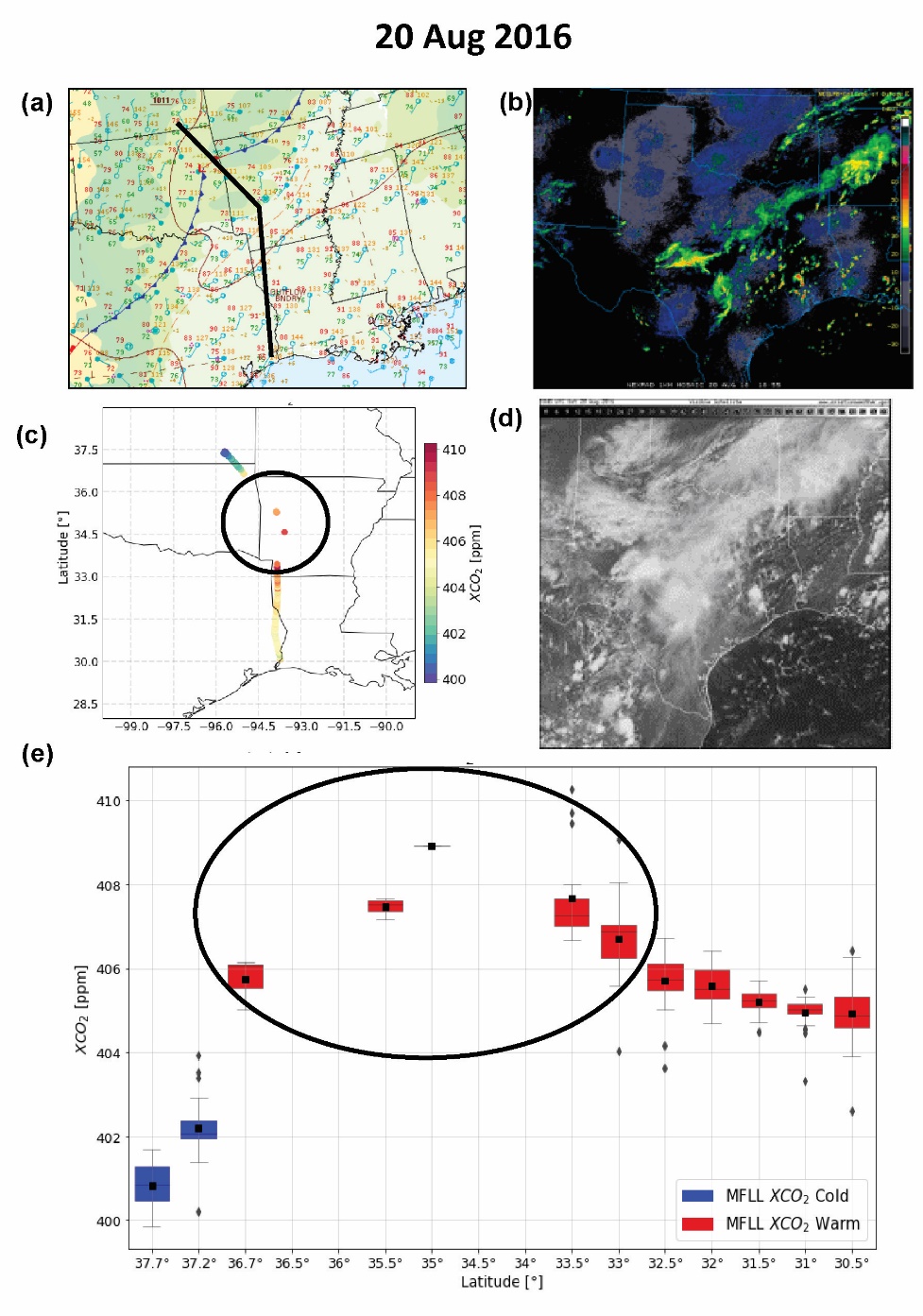
***

***Figure S2:*** *Spatial variability in the XCO_2_ field from an altitude of 4.5 km MSL observed on 20 Aug 2016 during a frontal RF in the South RF in northwestern Arkansas illustrating the frontal enhancement of XCO_2._ Surface synoptic map with the approximate C-130 flight track (black line) overlaid (a). Radar observations of precipitation field (b). The MFLL XCO_2_ along the flight track (c). Satellite imagery indicating the presence of extensive clouds (d). Box and whisker plot with 0.5° latitude average MFLL XCO_2_ boxes colored by respective warm (red) and cold (blue) sectors, with the marking associated with the boxes being the same as in Fig. 1e. A region of enhancement of XCO_2_ is indicated in the box and whisker plot by the black oval area in both panels (c) and (e).*

*
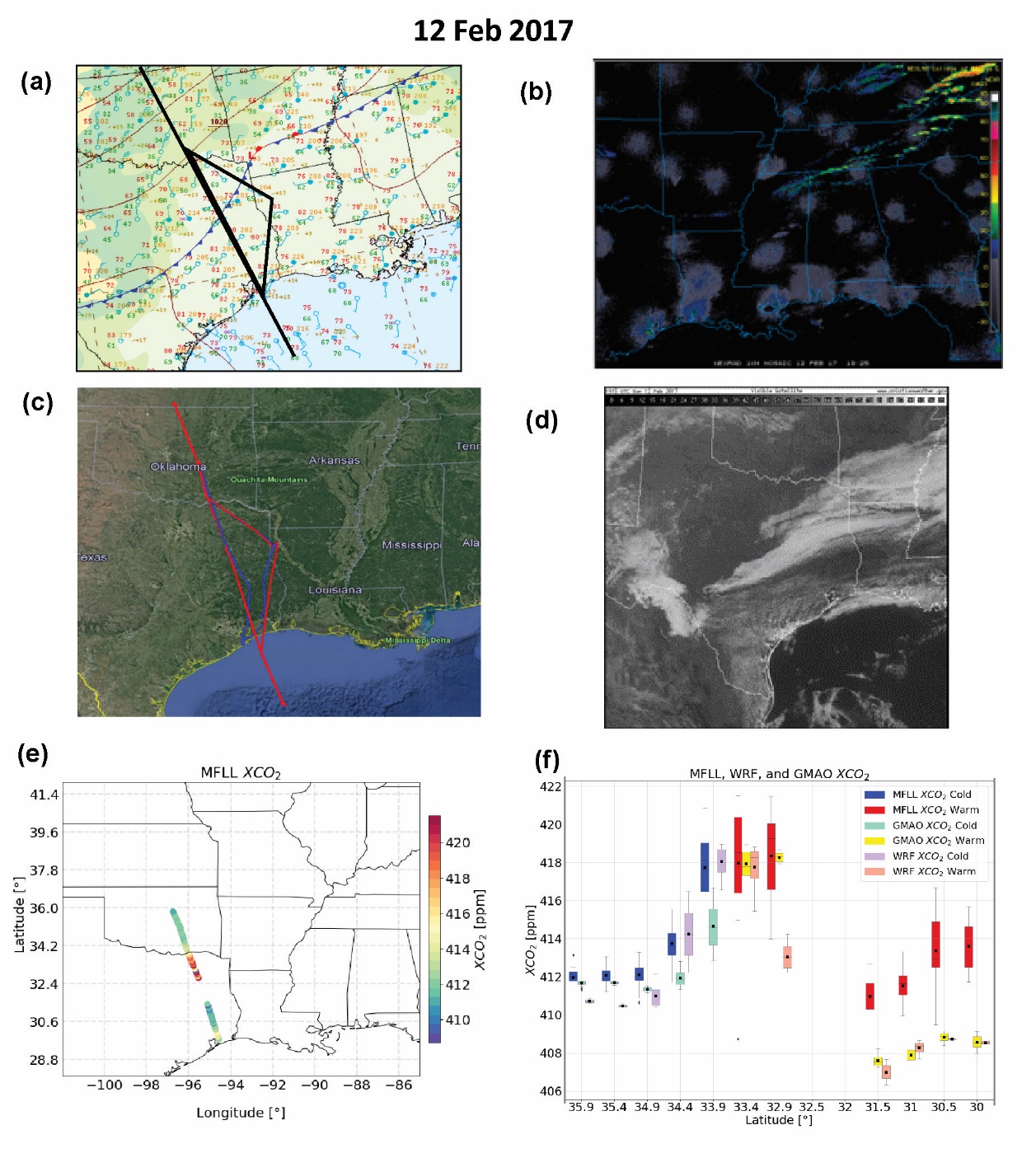
*

***Figure S3:*** *a) Frontal RF crossing through eastern Texas and Oklahoma with a cold front that was sampled in NE Texas on 12 Feb 2017. No precipitation was in the region as indicated by radar (b) The flight track is indicated by (c), with the red flight track being the C-130 and the blue tracks being the B-200. Satellite imagery (d) indicated clouds collocated with the region of enhancement. MFLL-derived XCO_2_ spatial variability from 2 km MSL altitude (e) and associated box-and-whisker analyses summarizing the XCO_2_ across the entire flight path binned into 0.5⁰ longitude segments for the MFLL, WRF-Chem, and GMAO XCO_2_ (f).*

*
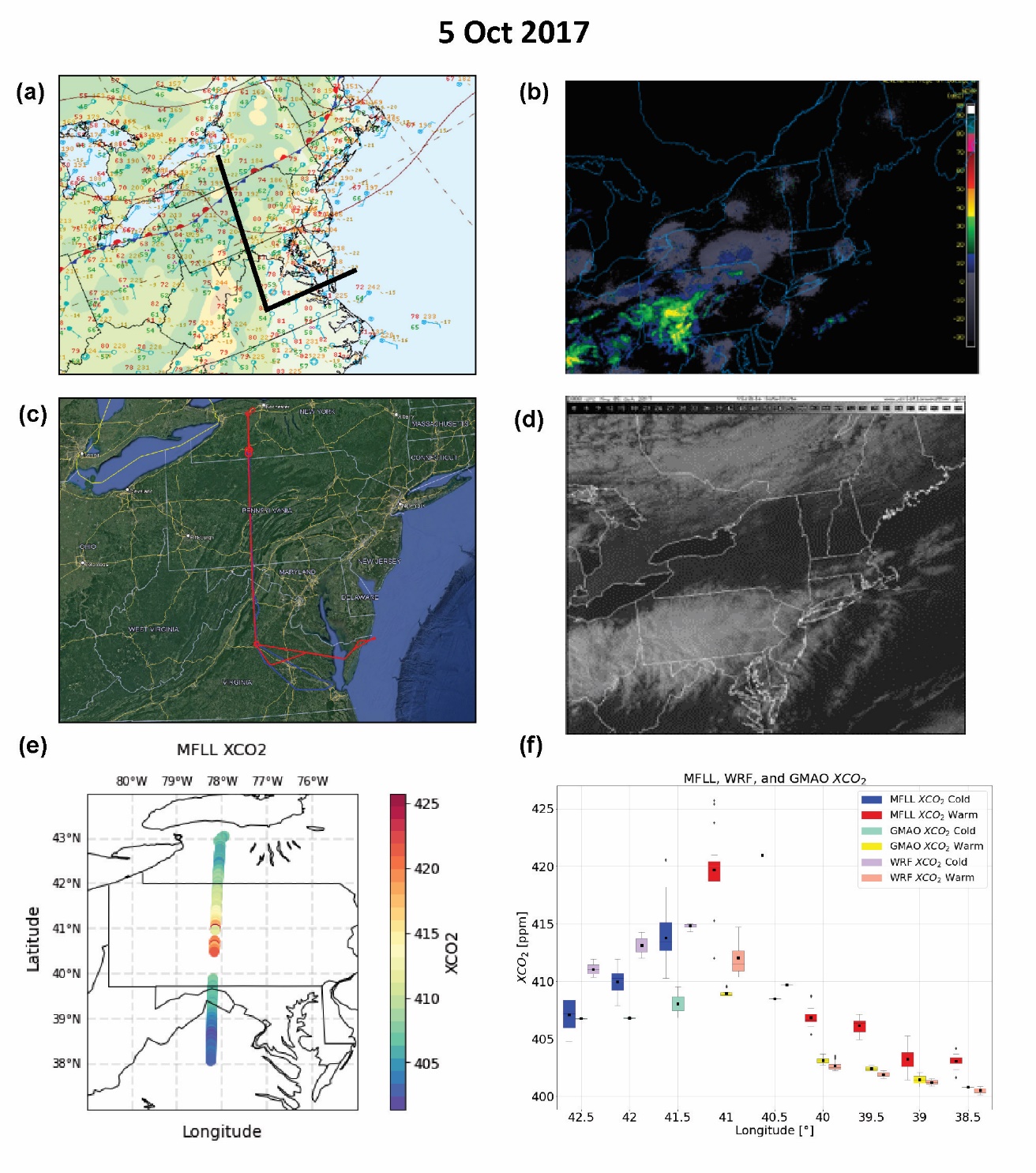
*

***Figure S4:*** *An example cold front crossing RF over the central PA region on 5 Oct 2017. Widespread precipitation was in the region as indicated by radar (b) The flight track is indicated by (c), with the red flight track being the C-130 and the two blue t tracks being the B-200. Satellite imagery (d) indicated clouds collocated with the region of enhancement. Spatial variability of XCO_2_ across the flight track obtained for 2.5 km MSL flight altitude (e). (f)* *Summary of the XCO_2_ across the entire flight path binned into 0.5⁰ longitude segments for the MFLL, WRF-Chem, and GMAO XCO_2_, and depicting the enhancement of XCO_2_ seen at the frontal boundary by both the observed MFLL XCO_2_ and the models (f).*

***
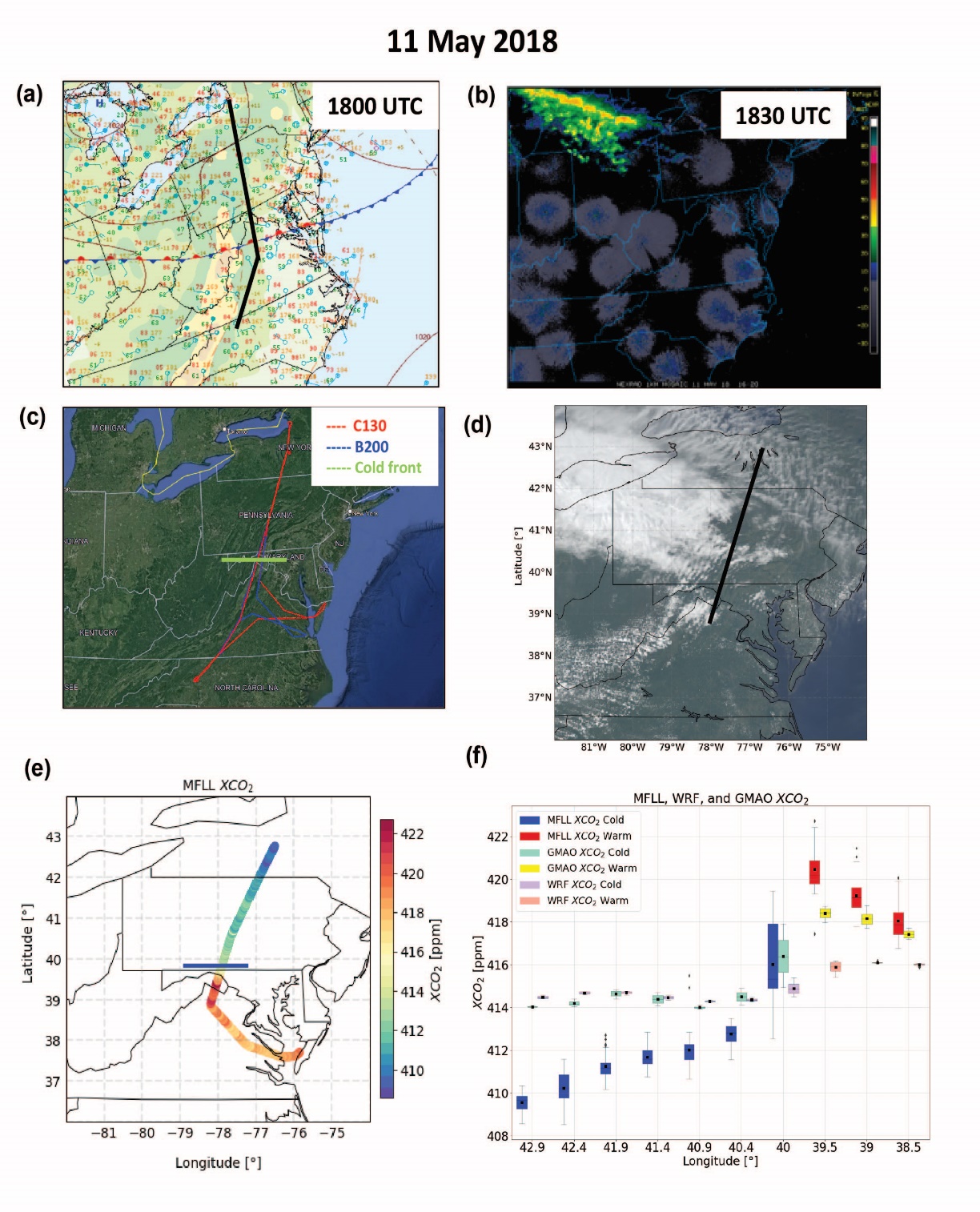
***

***Figure S5****: Frontal RF on 11 May 2018 during the ACT-America Spring field campaign showing the surface synoptic set up (a), and flight tracks crossing through the cold front in northern VA (c). No precipitation was in the region of the frontal crossing as indicated by radar (b). Satellite imagery (d) indicated limited clouds collocated with the region of XCO_2_ enhancement. Spatial variability in XCO_2_ field (e) and associated box-and-whisker analyses (f) based on the MFLL measurements.*

***
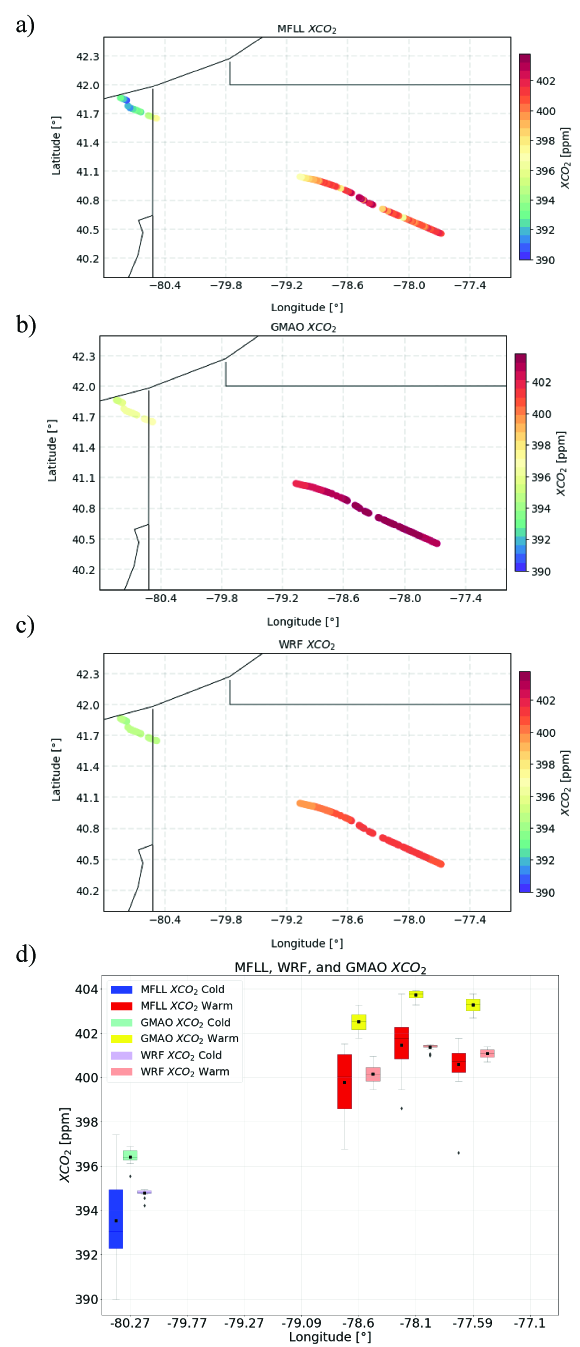
***

***Figure S6.***  *(a) Spatial variability in the XCO_2_ field obtained using MFLL (a), GMAO (b) and WRF-Chem (c) on 18 July 2016. Box and whisker diagram depicting XCO_2_ simulated by both the GMAO and WRF-Chem compared with the observed MFLL XCO_2_.*

***
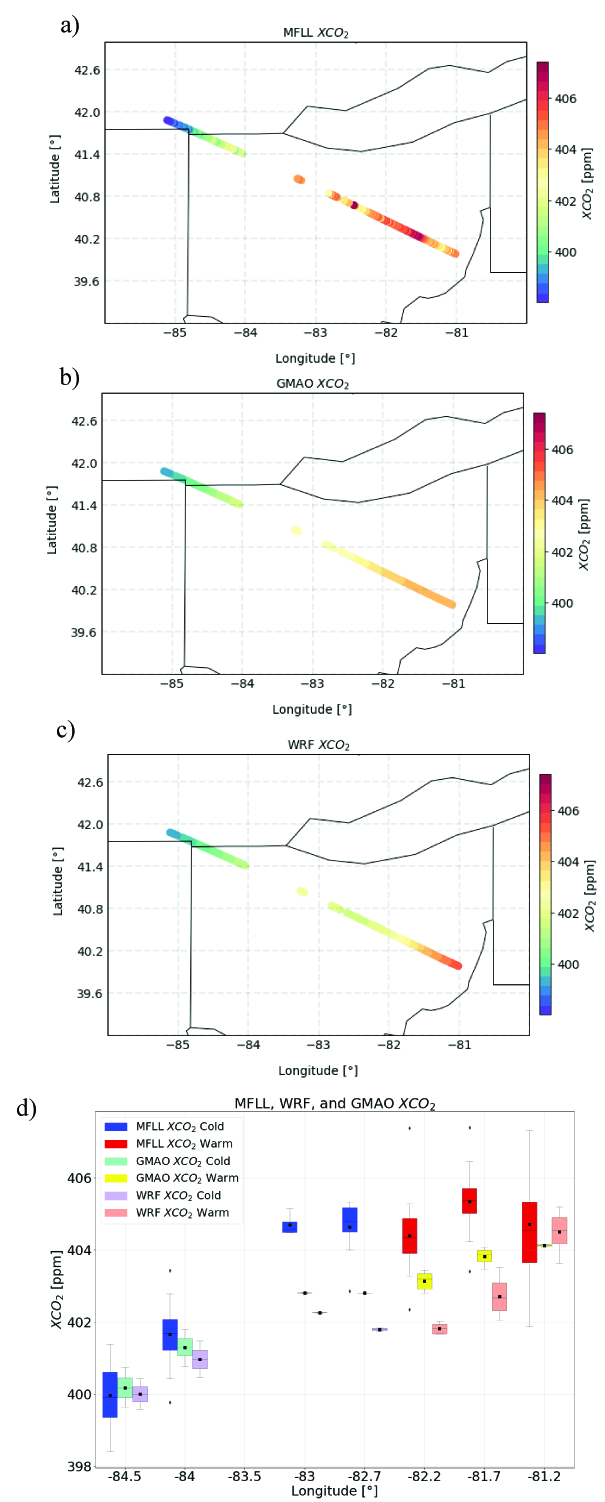
***

***Figure S7.*** *Same as Fig. S6 but for 25 July 2016 in the MA region at 8 km flight level.*

***
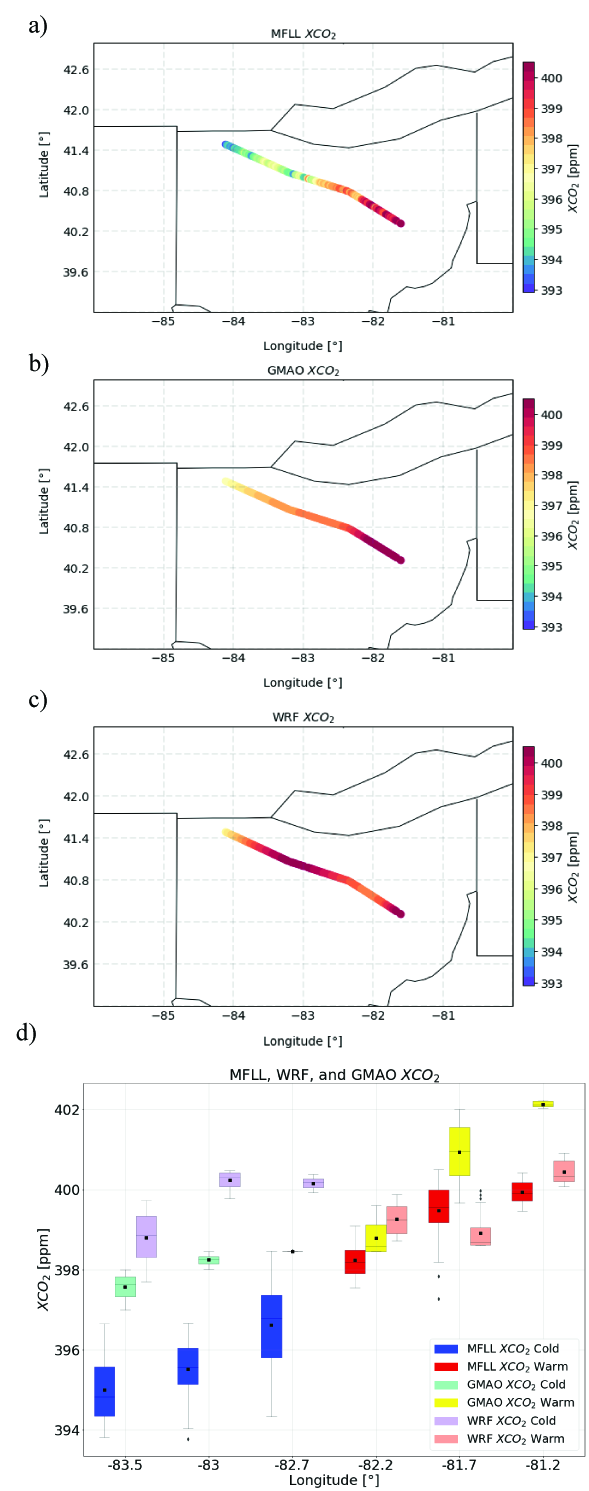
***

***Figure S8.*** *Same as Fig. S6 but for 25 July 2016 in the MA region at 3 km flight level.*

*
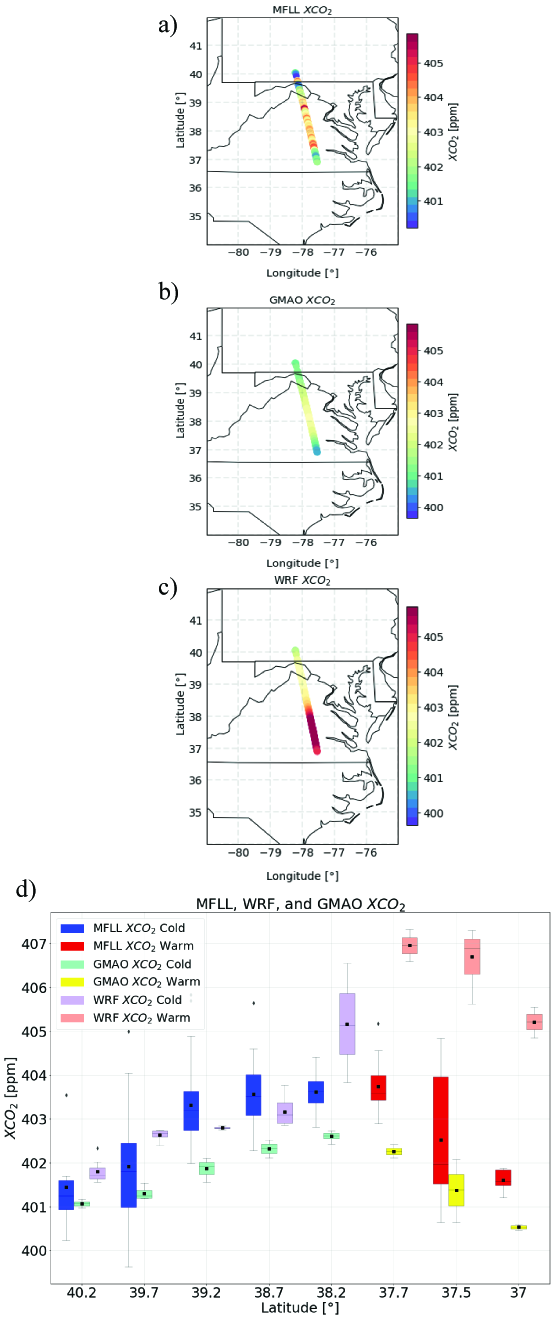
*

***Figure S9.*** *Same as Fig. S6 but for 26 July 2016 in the MA region.*

***
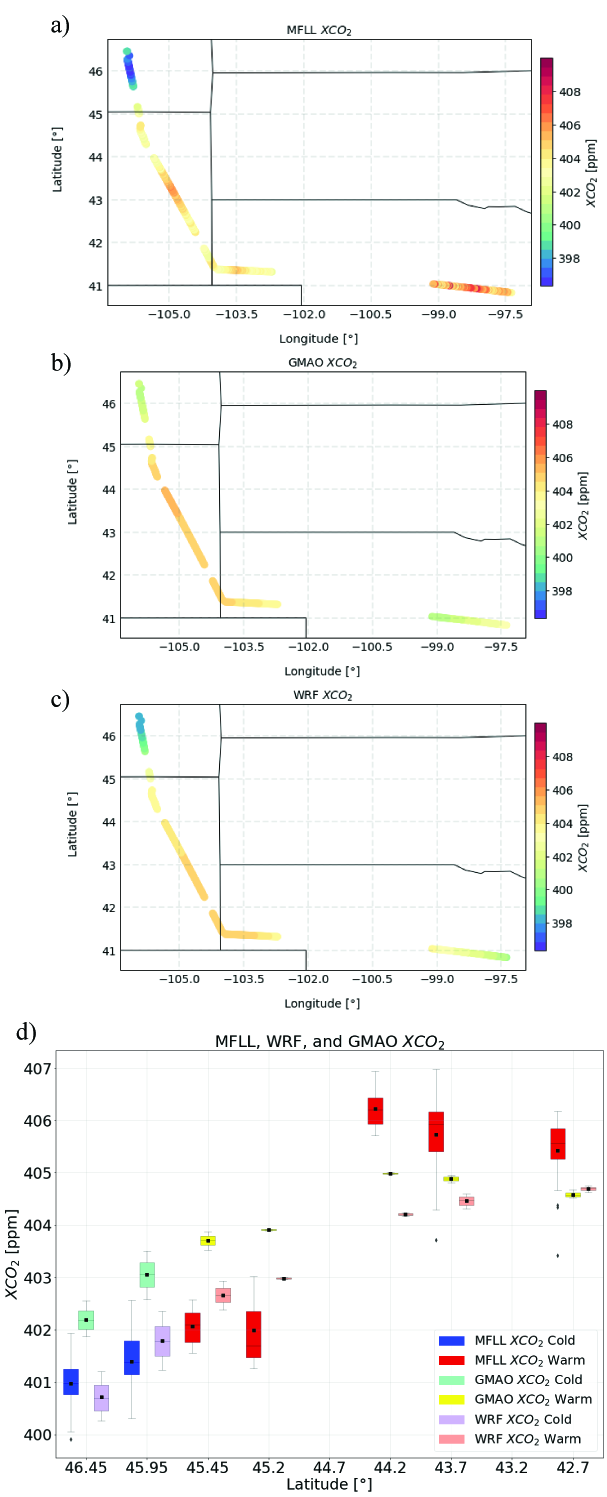
***

***Figure S10.*** *Same as Fig. S6 but for 3 August 2016 in the MW region at 8 km flight level.*

***
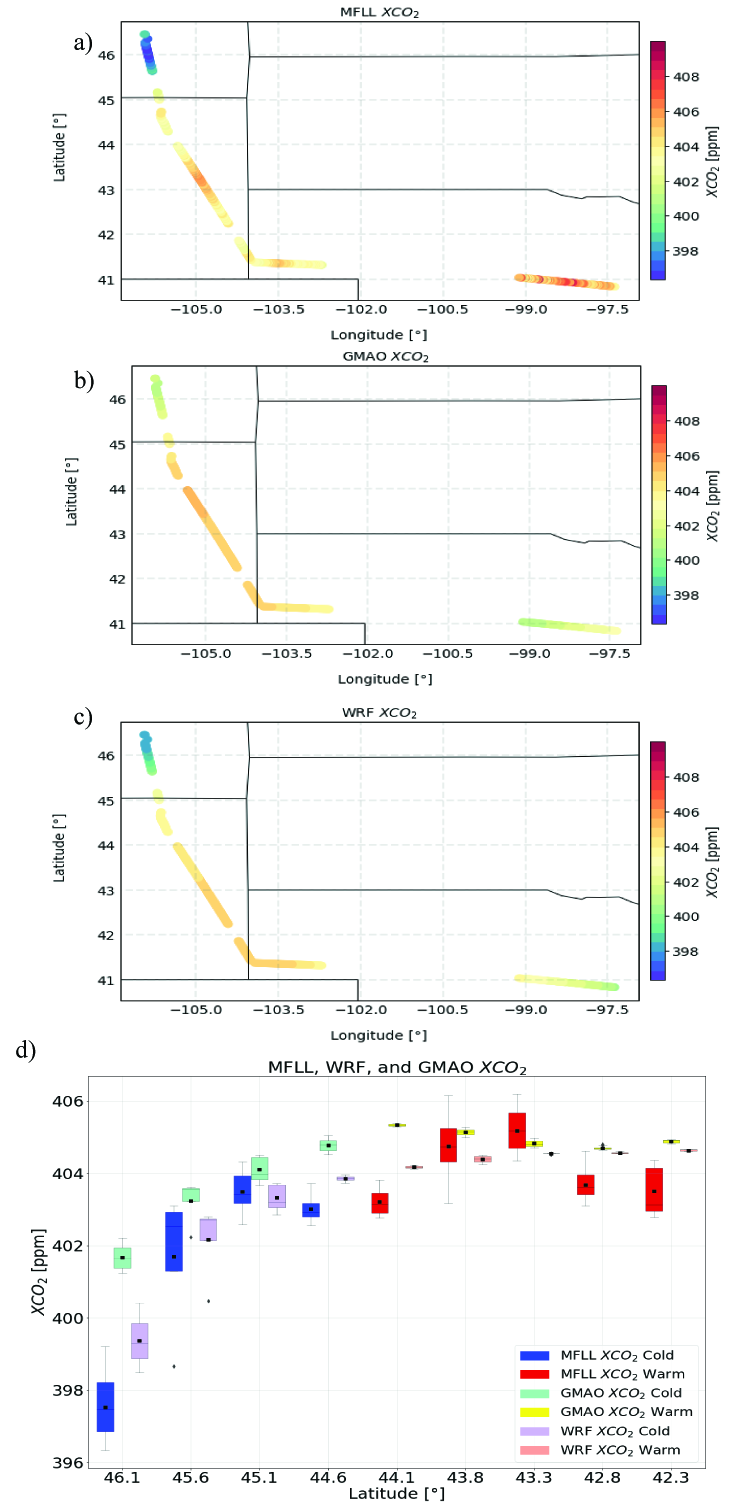
***

***Figure S11.*** *Same as Fig. S6 but for 3 August 2016 in the MW region at 4 km flight level.*

***
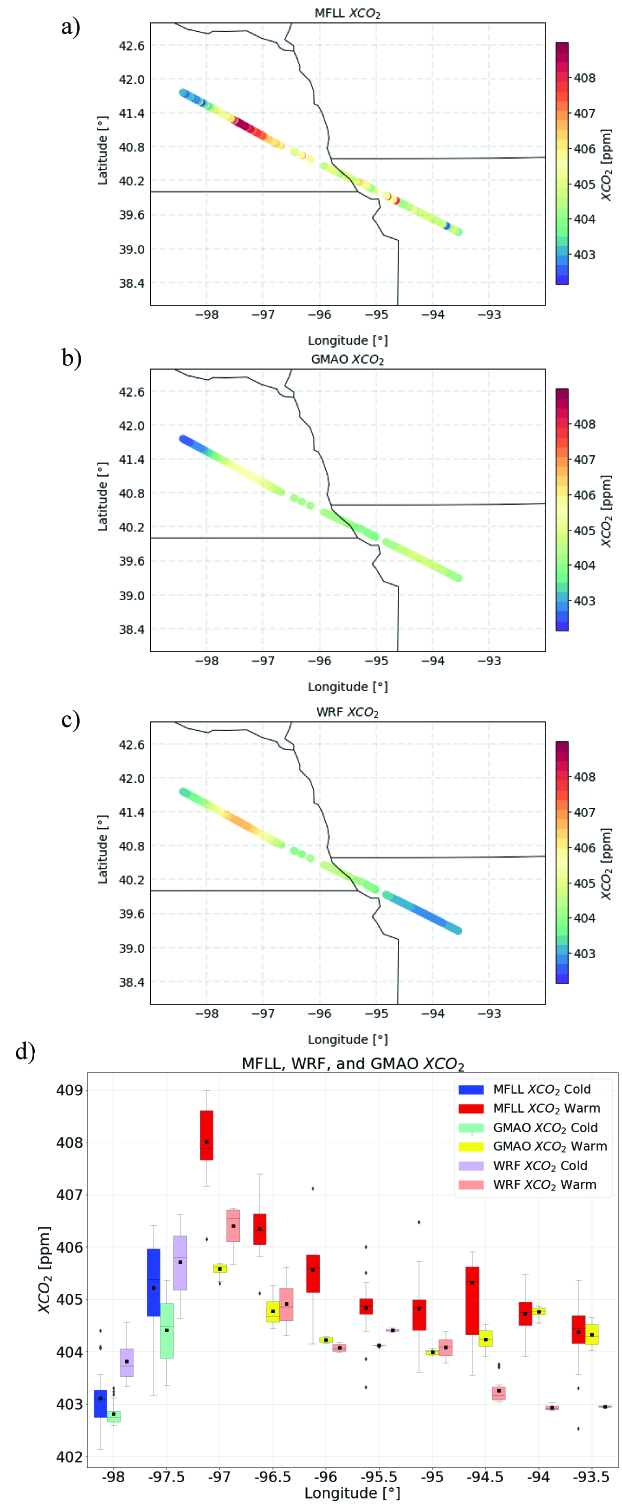
***

***Figure S12.*** *Same as Fig. S6 but for 4 August 2016 in the MW region.*

***
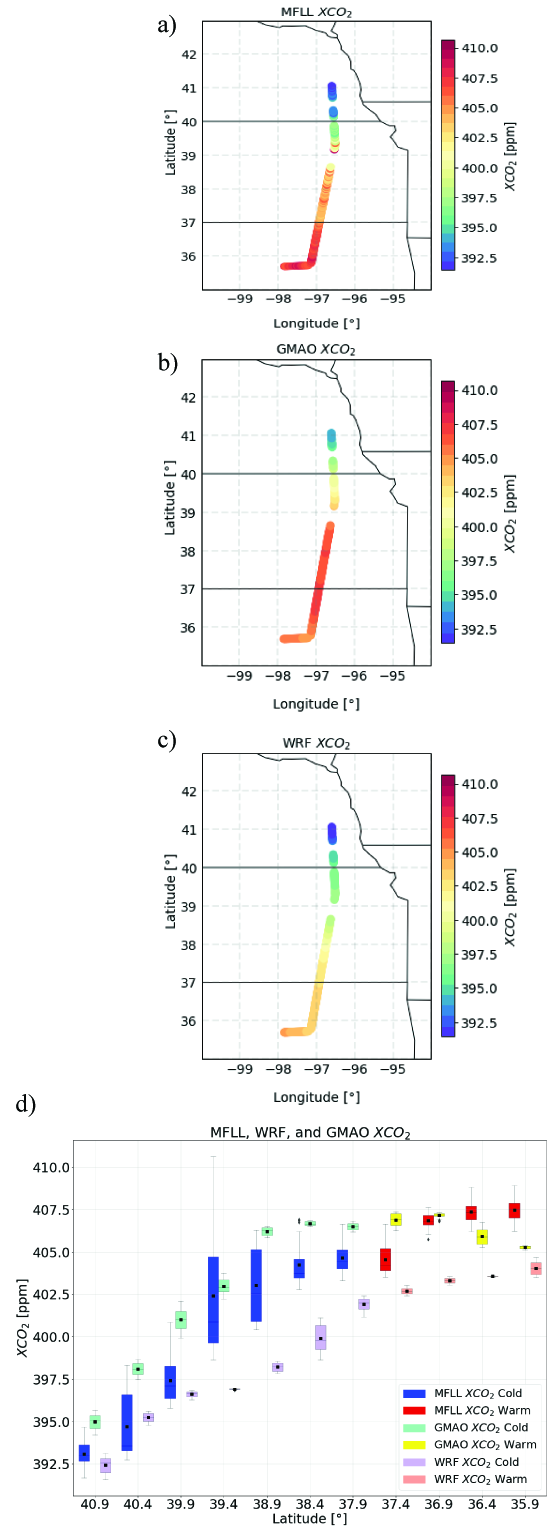
***

***Figure S13.*** *Same as Fig. S6 but for 8 August 2016 in the MW region.*

*
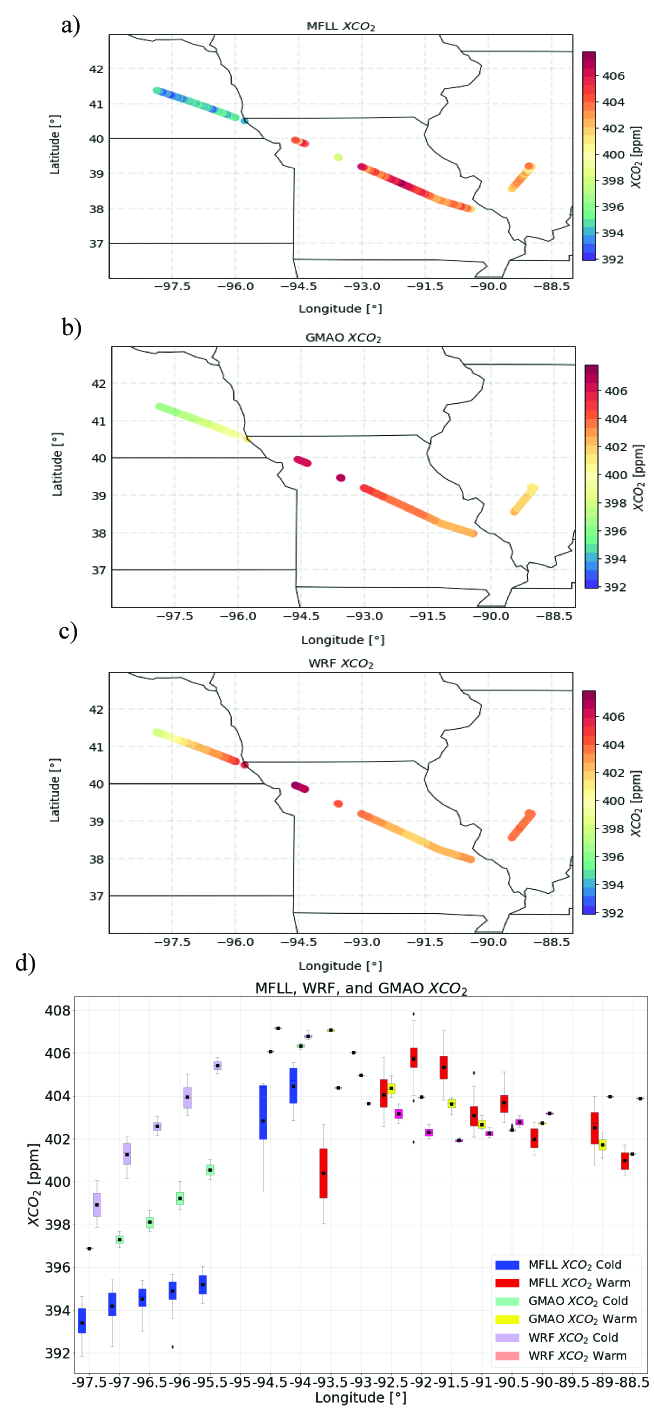
*

***Figure S14.*** *Same as Fig. S6 but for 12 August 2016 in the MW region.*

*
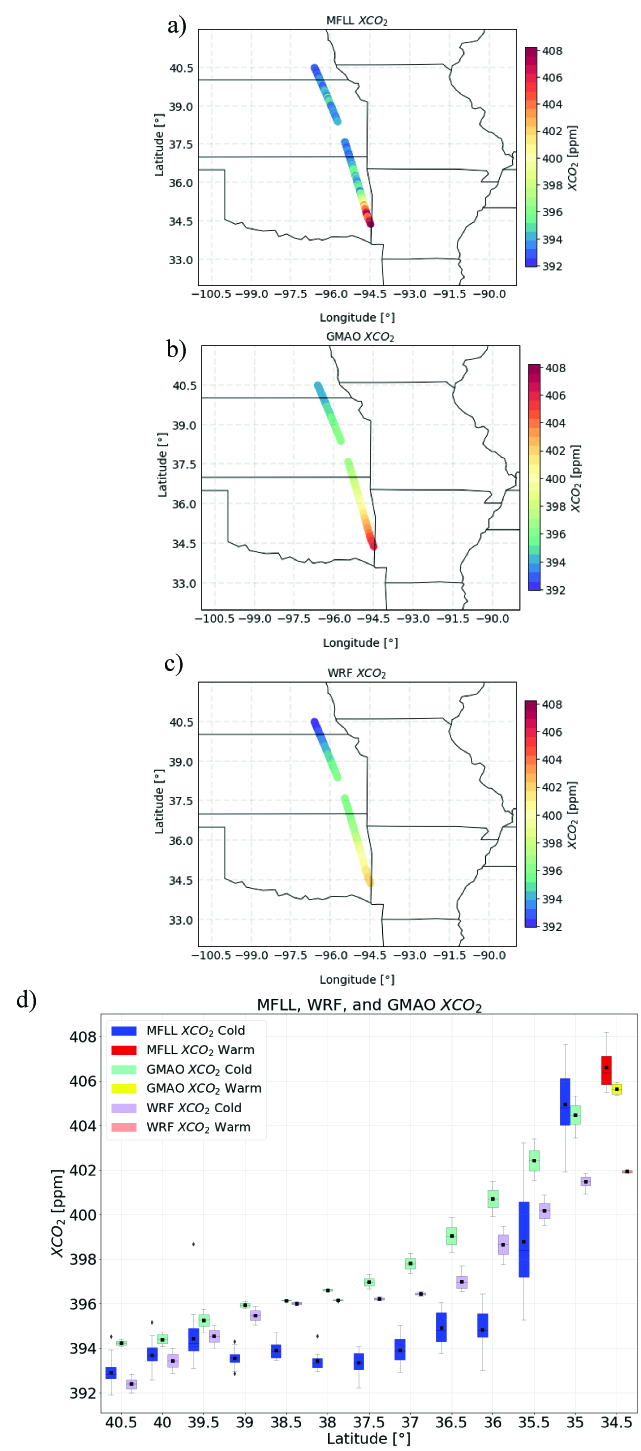
*

***Figure S15.*** *Same as Fig. S6 but for 16 August 2016 in the MW region.*

***
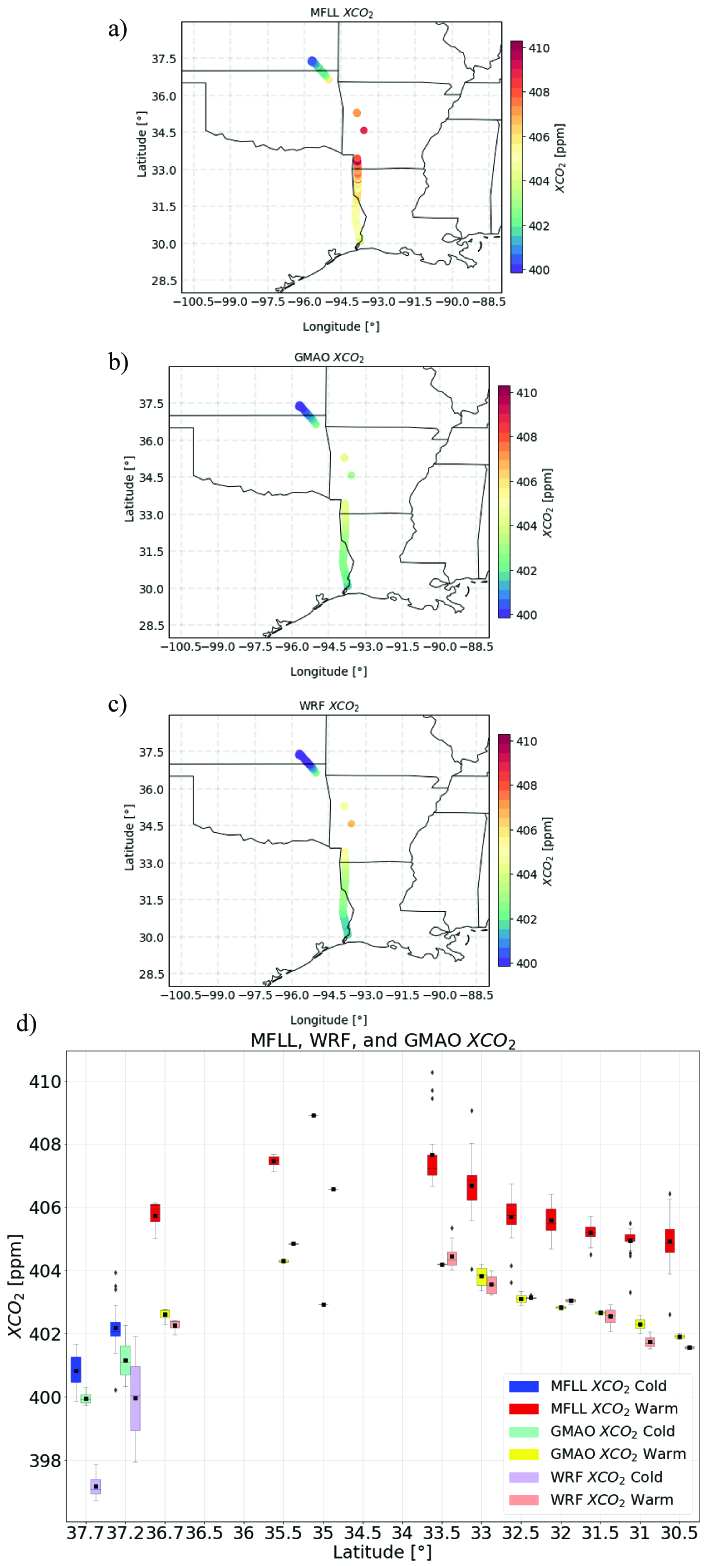
***

***Figure S16.*** *Same as Fig. S6 but for 20 August 2016 in the SO region at 4.5 km flight level.*

***
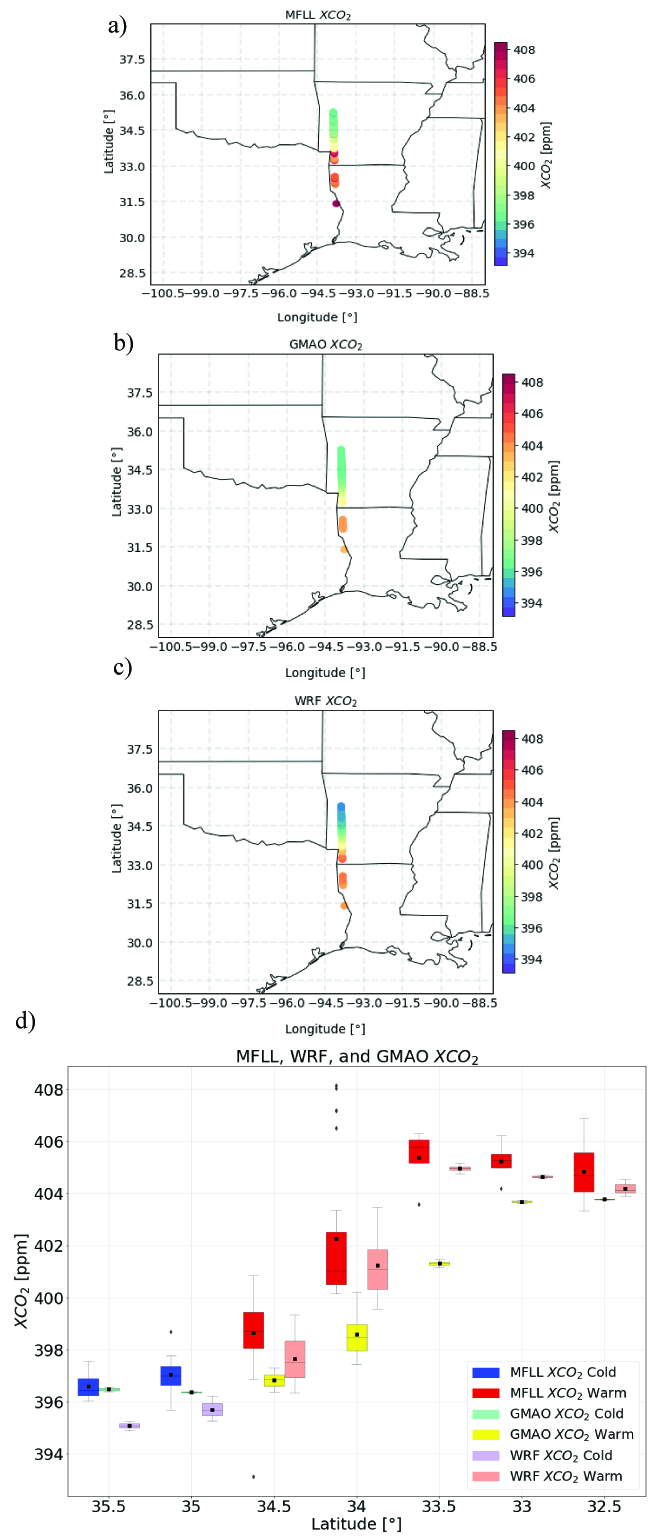
***

***Figure S17.*** *Same as Fig. S6 but for 21 Aug 2016 in the SO region.*

*
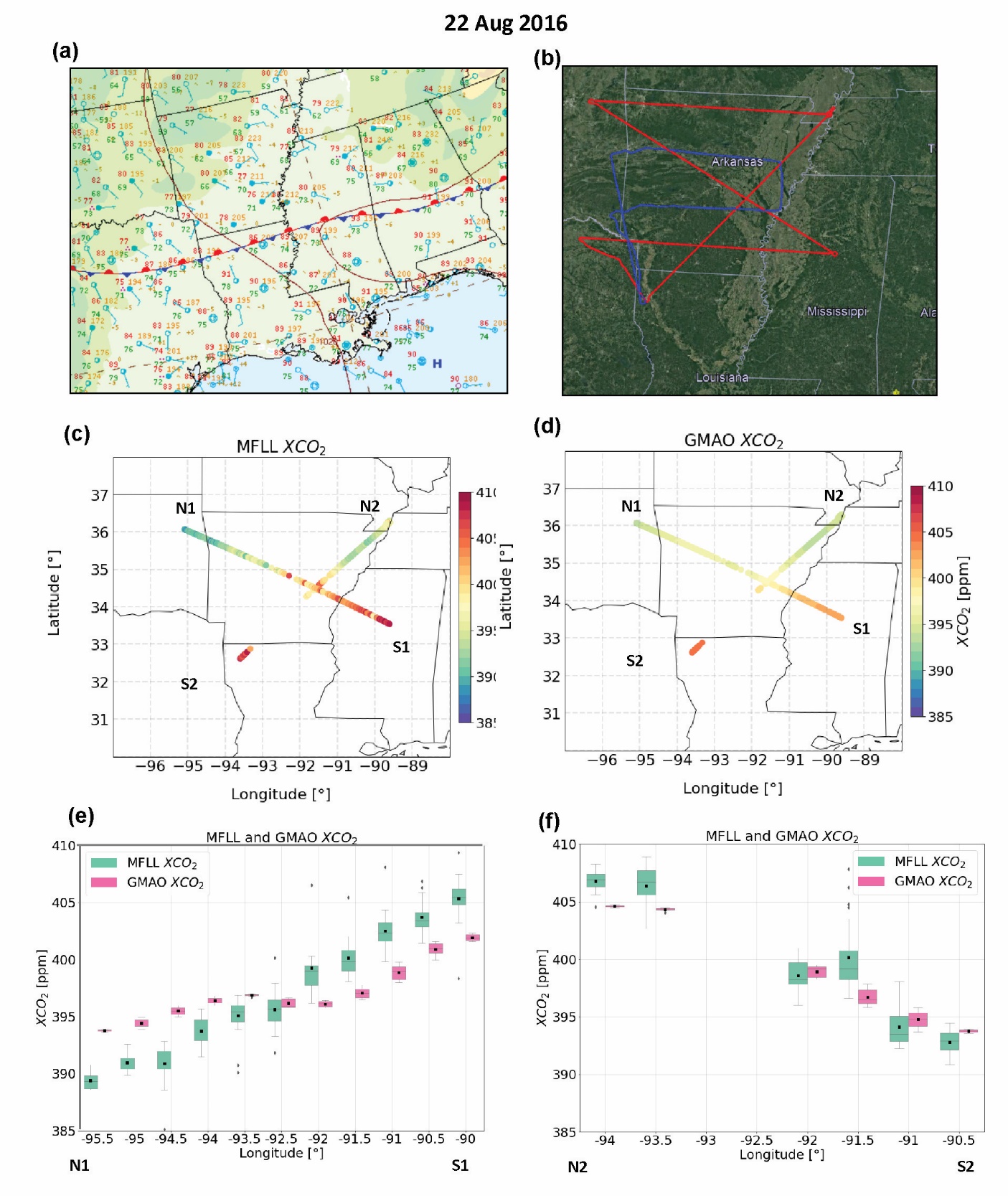
*

***Figure S18.*** *Model-data differences obtained for fair weather case on 22 Aug 2016 under a high-pressure condition (a). Spatial variability of XCO_2_ along the flight track (b) obtained by the retrievals from MFLL (c) and GMAO (d); Box and whisker plot of the XCO_2_ along N1-S1 (e) and N2-S2 (f) legs (XCO_2_ in 0.5⁰ longitude segments) for the MFLL (green), and GMAO (pink).*

***
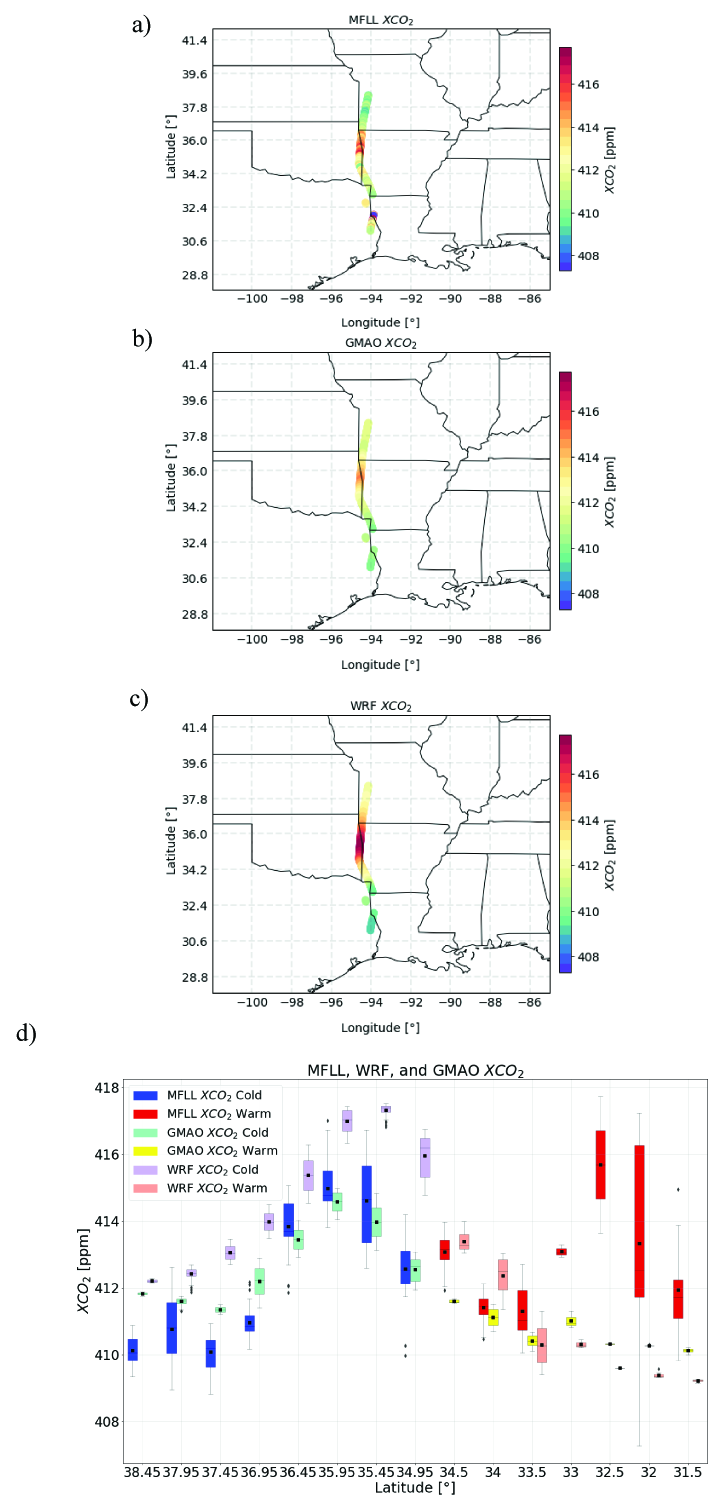
***

***Figure S19.*** *Same as Fig. S6 but for 1 Feb 2017 in the SO region.*

*
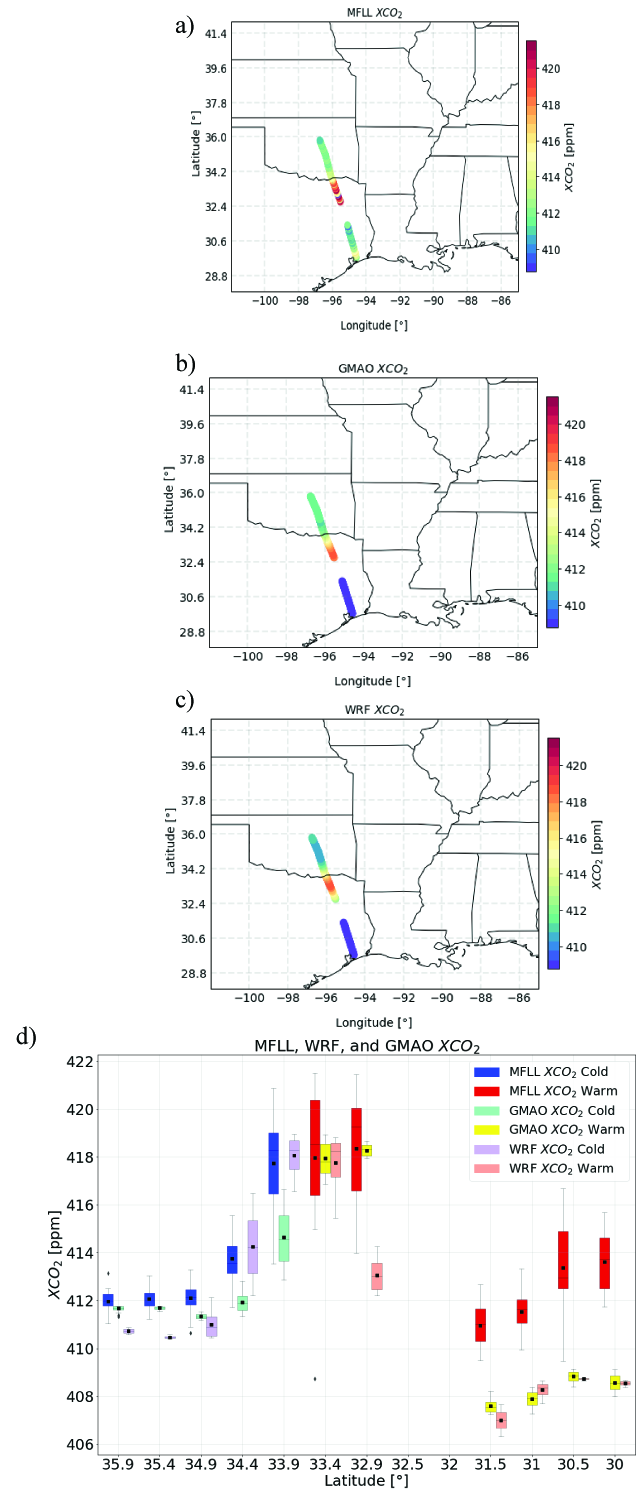
*

***Figure S20.*** *Same as Fig. S6 but for 12 Feb 2017 in the SO region.*

*
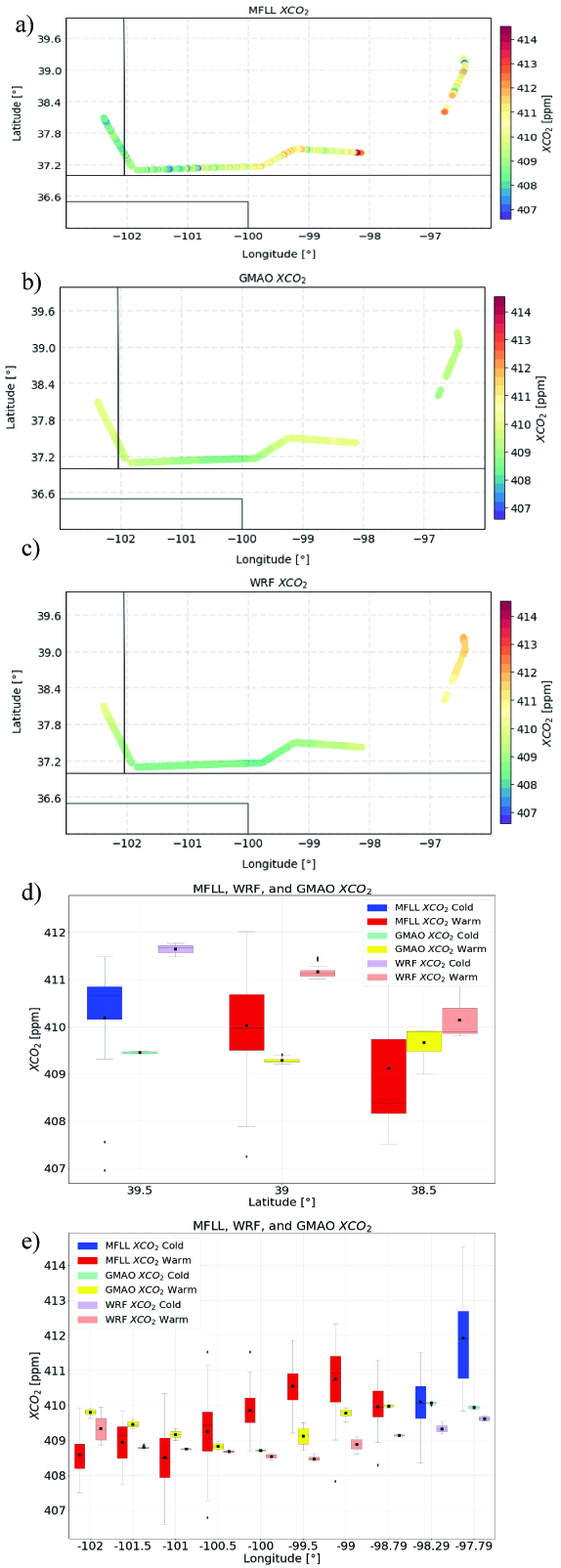
*

***Figure S21.*** *Same as Fig. S6 but for 23 February 2017 in the MW region at 6 km flight level. (d) is the longitudinal portion of the flight with (e) being the latitudinal portion of the flight.*

***
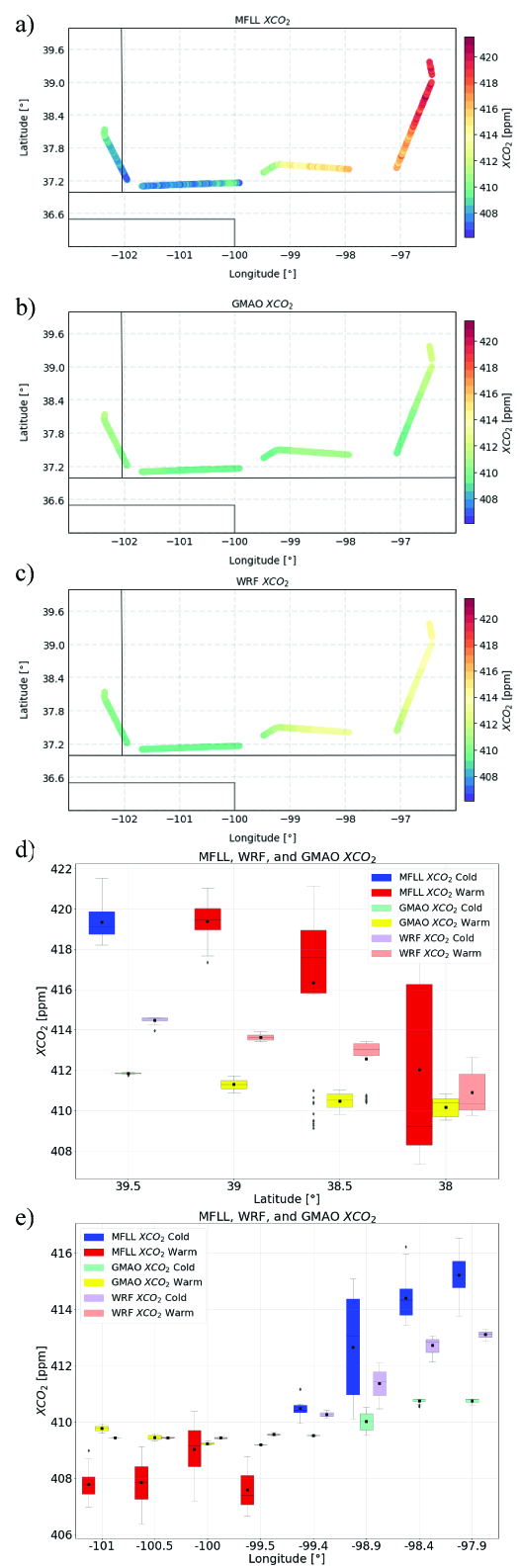
***

***Figure S22.*** *Same as Fig. S6 but for 23 February 2017 in the MW region at 3 km flight level. (d) is the longitudinal portion of the flight with (e) being the latitudinal portion of the flight.*

*
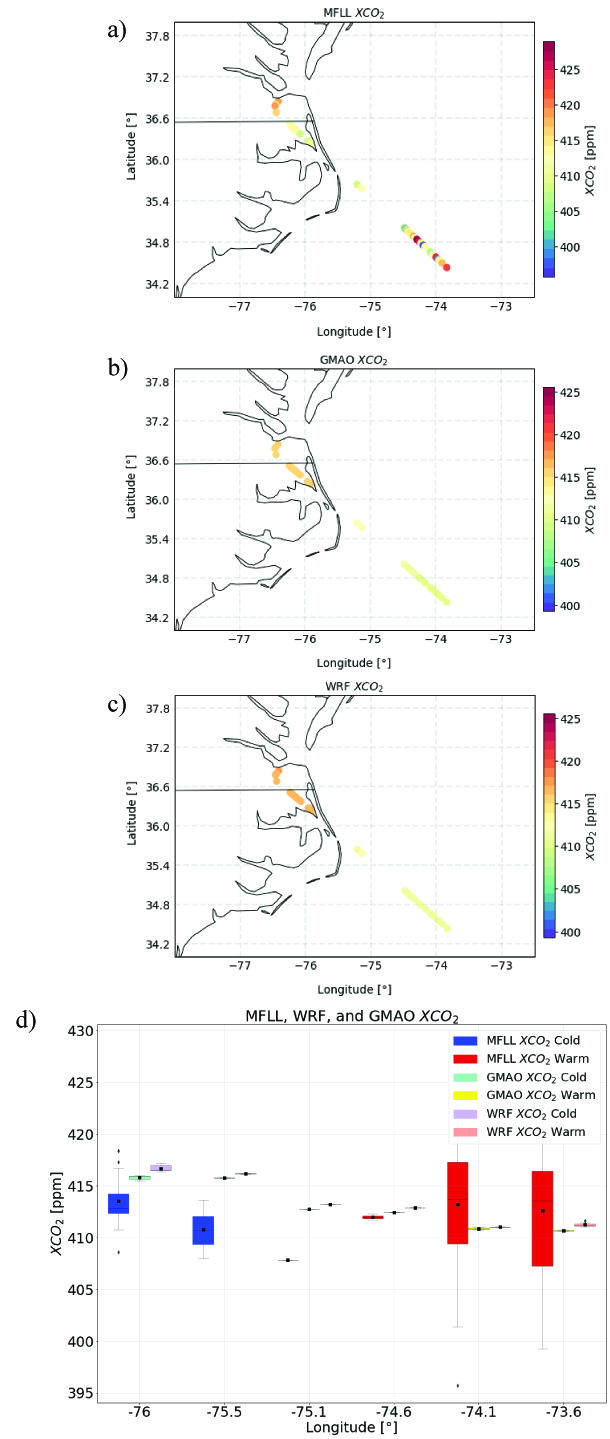
*

***Figure S23.*** *Same as Fig. S6 but for 10 Mar 2017 in the MA region at 2.5 km flight level.*

*
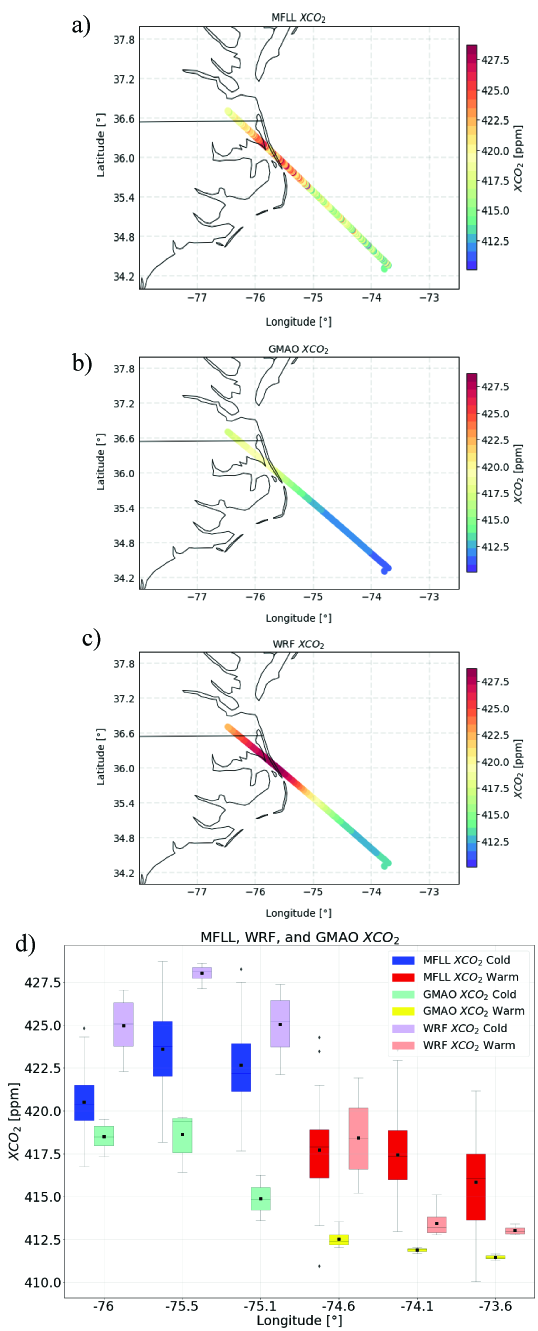
*

***Figure S24.*** *Same as Fig. S6 but for 10 Mar 2017 in the MA region at 1 km MSL flight level.*

***
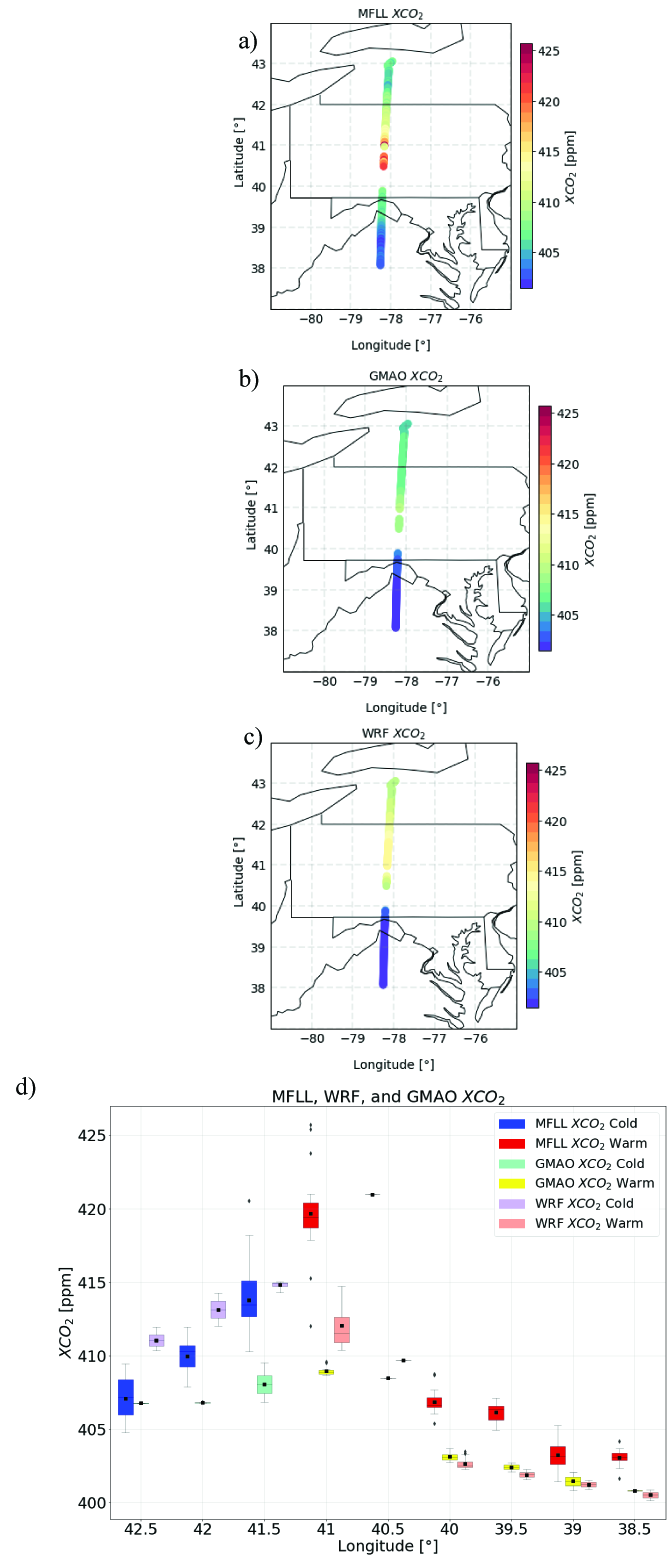
***

***Figure S25.*** *Same as Fig. S6 but for 5 Oct 2017 in the MA region.*

*
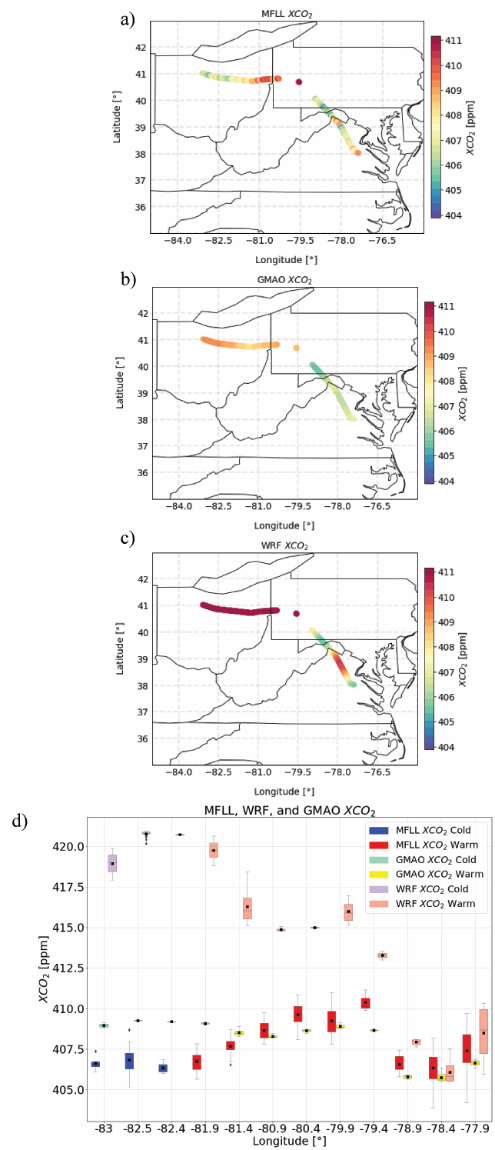
*

***Figure S26.*** *Same as Fig. S6 but for 8 Oct 2017 in the MA region.*

***
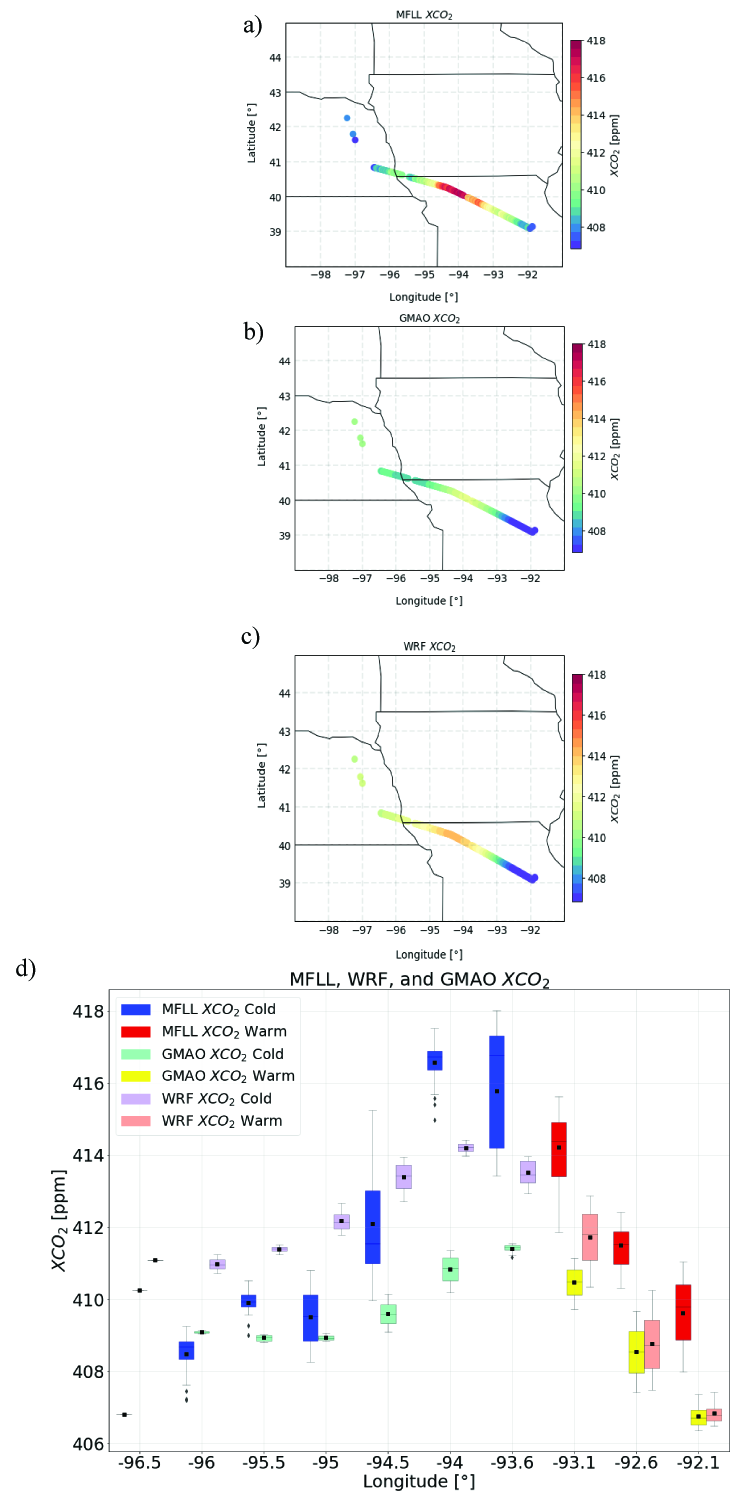
***

***Figure S27.*** *Same as Fig. S6 but for 26 Oct 2017 in the MW region.*

*
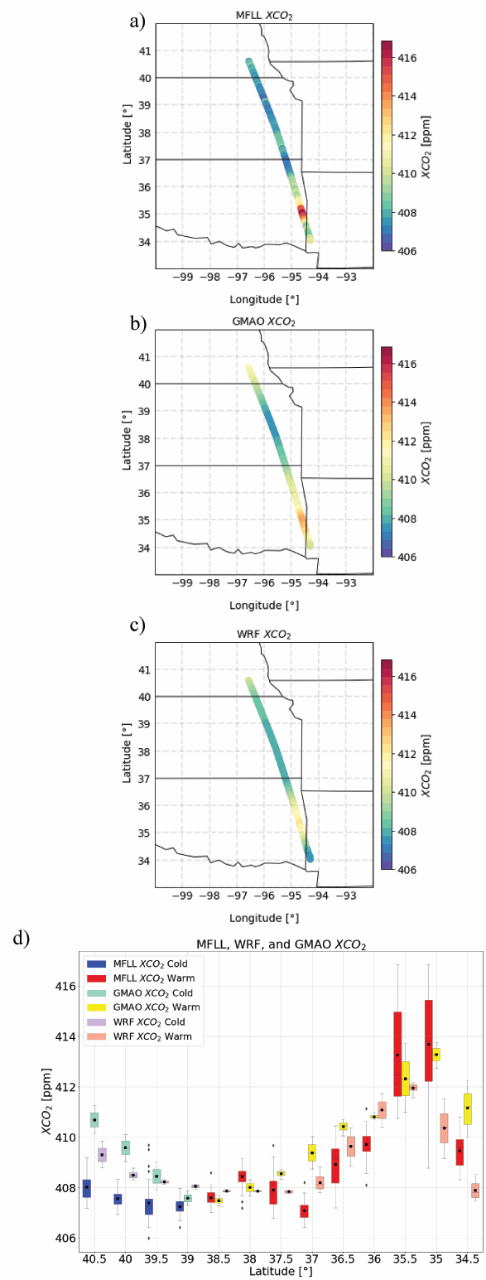
*

***Figure S28.*** *Same as Fig. S6 but for 30 Oct 2017 in the MW region.*

*
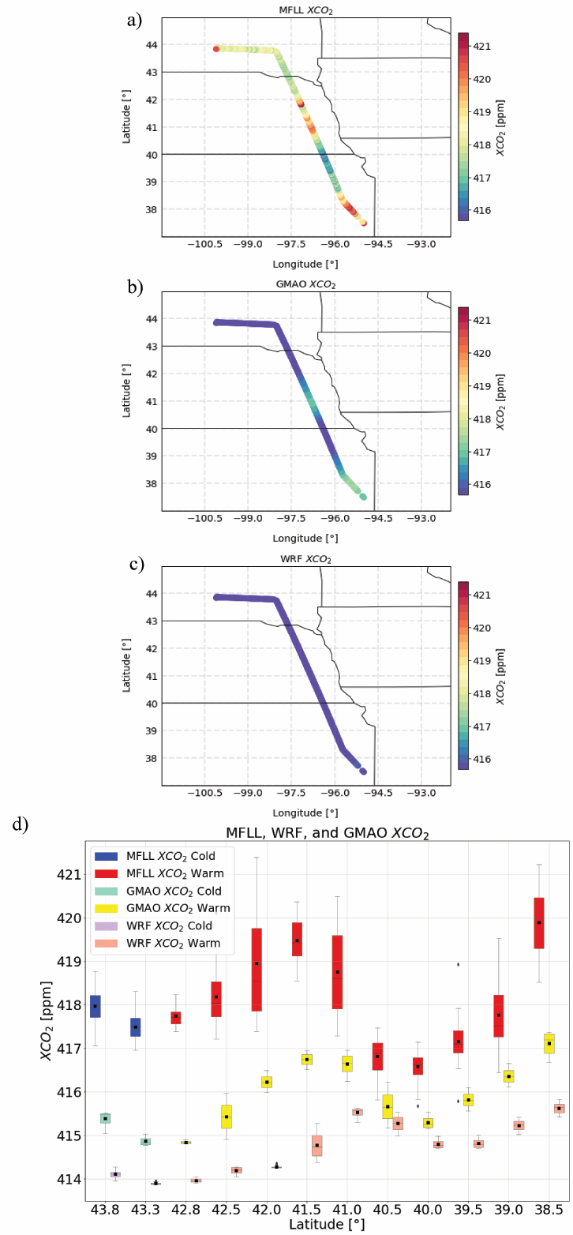
*

***Figure S29.*** *Same as Fig. S6 but for 26 April 2018 in the MW region.*

*
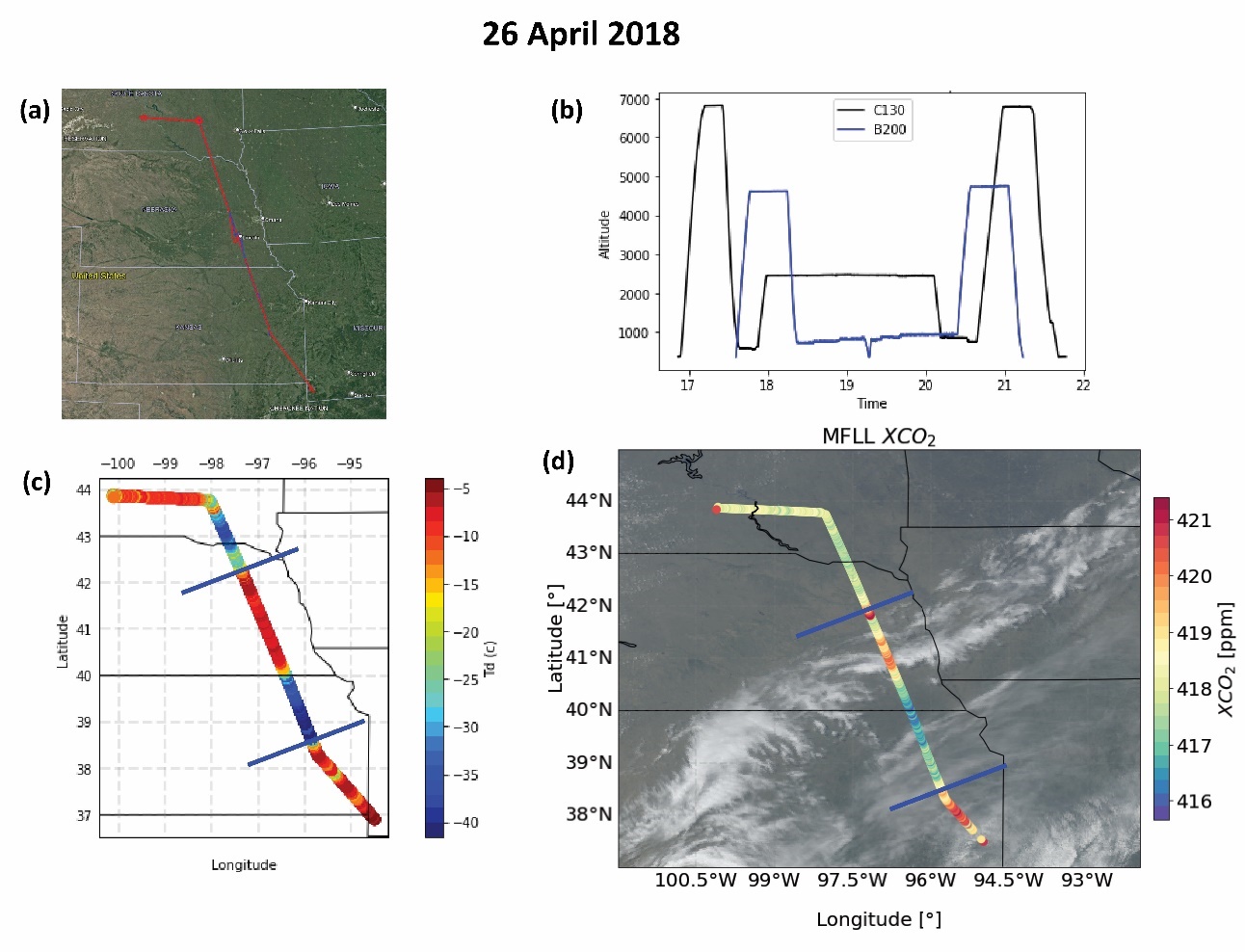
*

***Figure S29 Continued:*** *Measurements obtained on 26 April 2018. C130 flight track overlaid on Google map (a); Time-height display of the flight altitudes for both C130 and B200 flight tracks (b). Spatial variability in dewpoint temperature (T_d_ in ^o^C, see color bar) obtained on C130 platform flying at an altitude of 2.5 km MSL marking the locations of cold front boundaries at two locations in northern Nebraska and central Kansas regions (see blue solid lines overlaid). XCO_2_ spatial variability observed from an altitude of 2.5 km MSL illustrating the two XCO2 enhancements near the frontal boundaries.*

***
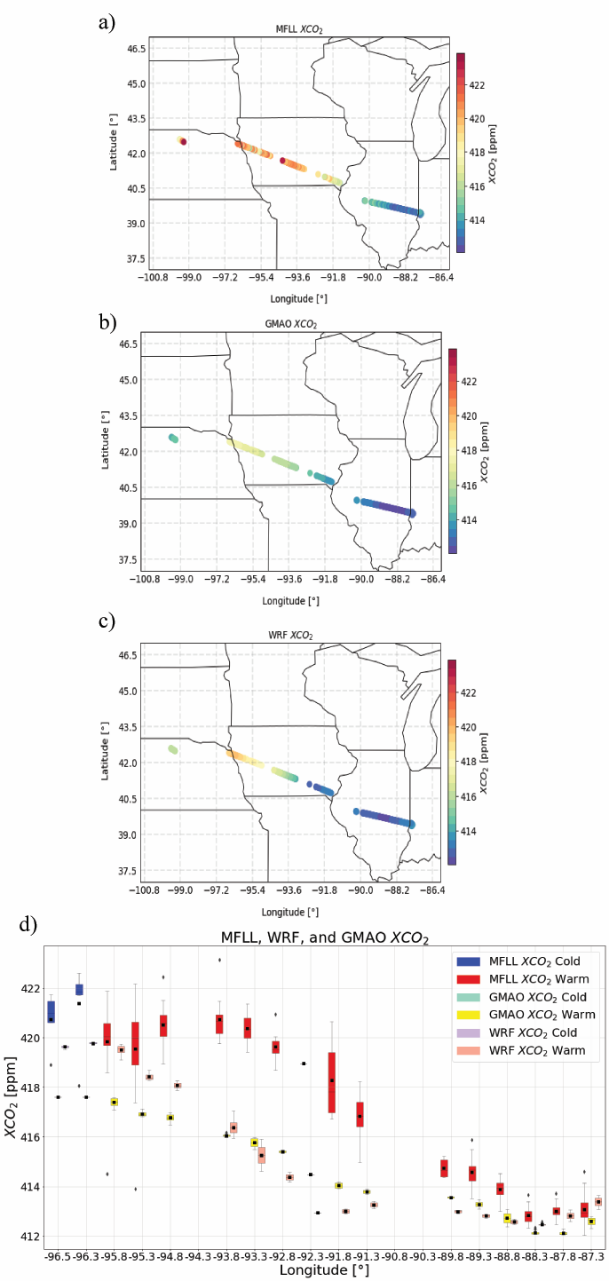
***

***Figure S30.*** *Same as Fig. S6 but for 1 May 2018 in the MW region.*

*
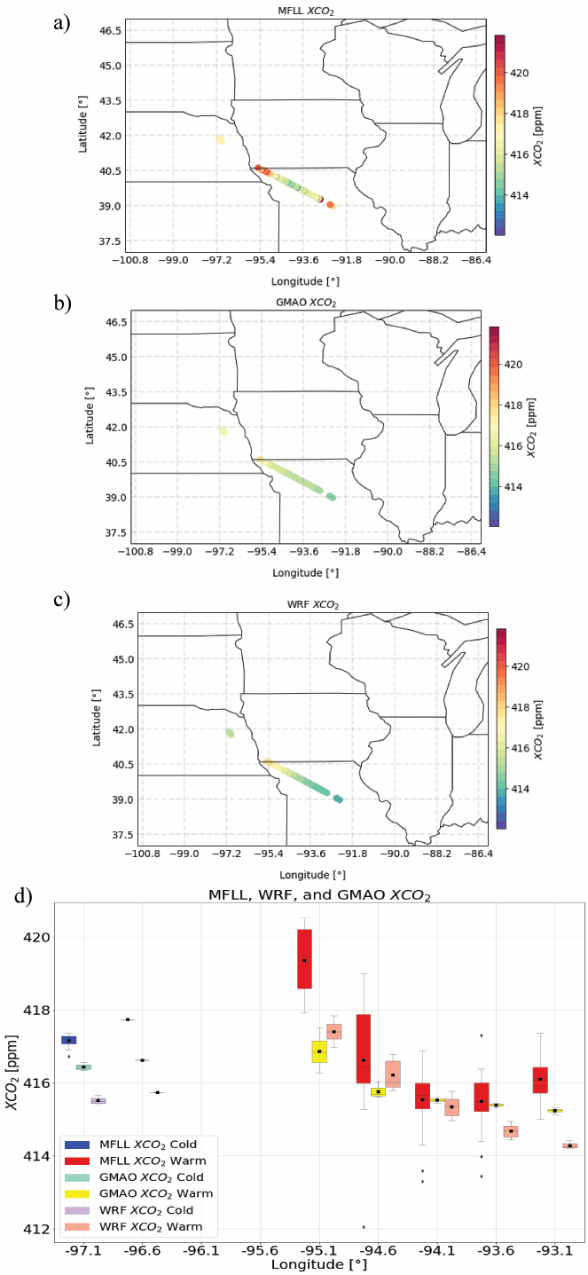
*

***Figure S31.*** *Same as Fig. S6 but for 2 May 2018 in the MW region.*
